# Mapping the Polar Neuro-Interactome of *Garcinia mangostana* Against the AD-PD-ALS Nexus

**DOI:** 10.3390/life16040580

**Published:** 2026-04-01

**Authors:** Rahni Hossain, Sirirat Surinkaew, Pradoldej Sompol, Nasmah K. Bastaki, Rifat Jafrin, Nazim Sekeroglu, Jitbanjong Tangpong

**Affiliations:** 1 Department of Medical Technology International, School of Allied Health Sciences, Walailak University, Nakhon Si Thammarat 80160, Thailand; rahni.hos@wu.ac.th (R.H.); sirirat.sr@wu.ac.th (S.S.); 2 Research Excellence Center for Innovation and Health Product (RECIHP), Walailak University, Nakhon Si Thammarat 80160, Thailand; 3 Department of Pharmacology & Nutritional Sciences, College of Medicine, University of Kentucky, Lexington, KY 40536, USA; pradoldej.sompol@uky.edu; 4 Department of Biological Science, Faculty of Science, Kuwait University, Kuwait City 13060, Kuwait; nasmah.bastaki@ku.edu.kw; 5 Institute of Biochemistry and Biology, Faculty of Science, University of Potsdam, 14476 Potsdam, Germany; jafrin@uni-potsdam.de; 6 Department of Biology, Faculty of Arts and Sciences, Gaziantep University, 27310 Gaziantep, Turkey; nsekeroglu@gmail.com

**Keywords:** *Garcinia mangostana*, neurodegenerative diseases, systems pharmacology, UHPLC–QTOF–MS metabolomics, blood–brain barrier permeability, Alzheimer’s disease, Parkinson’s disease, amyotrophic lateral sclerosis

## Abstract

**Background/Objectives**: Neurodegenerative diseases like Alzheimer’s, Parkinson’s, and Amyotrophic lateral sclerosis (ALS) share common molecular pathways, including neuroinflammation and oxidative stress, which complicate the effectiveness of single-target treatments. *Garcinia mangostana* L. (mangosteen) has shown neuroprotective properties, but previous studies focused on lipophilic xanthones, which have poor bioavailability and uncertain blood–brain barrier permeability. **Methods**: In the current study, polar metabolites from *G. mangostana* peel aqueous extract (GMPE) were assessed for potential multi-target interactions via UHPLC-QTOF-MS-based metabolomics, systems pharmacology, and molecular docking analysis. Further, in silico ADMET screening and network-based analyses assessed for overlap between GMPE compounds and genes associated with neurodegeneration (AD, PD, ALS). **Results**: Analysis of genes linked to AD, PD, and ALS revealed 121 common molecular targets influenced by GMPE metabolites. Network and enrichment analyses indicated that the compounds derived from GMPE may be involved in common pathways related to oxidative stress, neuroinflammation, and neuronal survival. Molecular docking analyses suggest that selected metabolites are likely to exhibit moderate binding affinities to their respective protein targets. **Conclusions**: The results presented in this study provide evidence that GMPE may possess potential multi-target interactions within common neurodegenerative pathways. However, since the data are based on computational and predictive approaches, these results should be considered hypothesis-generating and warrant further experimental validation.

## 1. Introduction

The trajectory of neurodegenerative pathology in the 21st century represents a significant challenge, as Alzheimer’s (AD), Parkinson’s (PD), and Amyotrophic Lateral Sclerosis (ALS) are increasingly recognized due to the ongoing deterioration of neuronal function [1]. While these conditions exhibit unique clinical characteristics, they share similar pathological processes, including persistent neuroinflammation, oxidative damage, mitochondrial impairment, disrupted proteostasis, synaptic dysfunction, and altered neuronal survival signaling [2]. These common processes indicate that neurodegeneration results from interconnected pathological networks, often called the neurodegenerative disease “nexus,” rather than from isolated molecular issues [3]. Despite many years of investigation, existing pharmacological treatments primarily alleviate symptoms and target single proteins or pathways [4]. Such simplistic approaches have consistently fallen short of stopping or reversing disease progression, highlighting the challenges of developing single-target drugs for intricate, multifactorial conditions [5]. As a result, there is increasing interest in therapeutic strategies that target multiple targets and adjust disease-relevant networks rather than individual molecular elements [6]. In this light, natural products and plant extracts have come back into focus as important sources of structurally diverse bioactive substances with inherent polypharmacological effects [7].

*Garcinia mangostana* L., known as mangosteen or the “Queen of Fruits,” has garnered considerable interest due to its extensive array of reported medicinal properties, including antioxidant, anti-inflammatory, anti-diabetic, cardioprotective, and neuroprotective effects [8]. Prior research has mainly attributed mangosteen’s bioactivity to its lipophilic xanthones, such as α-mangostin, which typically have poor water solubility and limited systemic absorption [9]. Numerous studies on α-mangostin highlight its potential, but its therapeutic efficacy is limited by poor pharmacokinetics. With a logP of 4.8–5.2 and low water solubility (<0.1 mg/mL), α-mangostin has low oral bioavailability (<10%) in rodents and does not cross the blood–brain barrier [10]. Research on mangosteen extracts in neurodegenerative disease models often focuses on lipophilic compounds, while the polar, water-soluble metabolite fraction of mangosteen peel remains poorly characterized and has been largely overlooked as a source of CNS-relevant bioactives [11]. Current studies typically examine isolated compounds or singular diseases, failing to reflect the complex interconnected nature of neurodegenerative disorders [12]. In contrast, polar metabolites with favorable CNS pharmacokinetics, such as certain fatty acid amides, coumarins, and water-soluble alkaloids, show promise due to their lower molecular weights and moderate lipophilicity, which facilitate passive diffusion across biological membranes, especially in older individuals at risk of neurodegenerative diseases [12]. Importantly, PHWE at 100 °C selectively enriches thermostable polar compounds while leaving highly lipophilic xanthones largely unextracted, yielding a chemically distinct, bioavailability-optimized fraction that has received almost no systematic characterization in the context of neurodegenerative disease research, representing a critical and unaddressed gap that the present study aims to fill [11]. This investigation highlights a significant lack of characterization of the fruit’s polar, water-soluble compounds, which might provide a better pharmacokinetic profile for central nervous system (CNS) uses [13]. The study employs Pressurized Hot Water Extraction (PHWE) to systematically capture highly bioavailable compounds while minimizing thermal degradation, thereby yielding a chemical profile that better reflects functional nutraceutical applications [14].

Recent advancements in systems pharmacology and network-based approaches provide robust tools for investigating the complex interactions between botanical metabolites, molecular targets, and disease pathways [15]. By utilizing metabolomics, target prediction, protein–protein interaction (PPI) networks, pathway enrichment analysis, and molecular docking, systems pharmacology enables a thorough evaluation of how multi-component extracts affect disease-relevant networks [16]. These methodologies are especially suitable for neurodegenerative conditions, where overlapping signaling pathways, such as PI3K–Akt, MAPK, autophagy, and inflammatory pathways, contribute to neuronal dysfunction and degeneration [17]. Importantly, AD, PD, and ALS show considerable overlap in dysregulation of genes and signaling pathways, including those related to neuroinflammation (e.g., TNF), oxidative stress (e.g., SOD1), protein aggregation (e.g., APP), and neuronal survival (e.g., AKT1) [18]. Therefore, targeting these shared molecular hubs may provide a strategic avenue for neuroprotection, delivering broad-spectrum defense instead of just symptomatic relief for individual diseases [19]. Nevertheless, no prior study has integrated PHWE-derived polar metabolite profiling with a multi-disease convergence framework spanning AD, PD, and ALS simultaneously, leaving a critical methodological gap in understanding how the water-soluble fraction of mangosteen peel may engage shared neurodegenerative mechanisms at a systems level.

**Figure 1:**
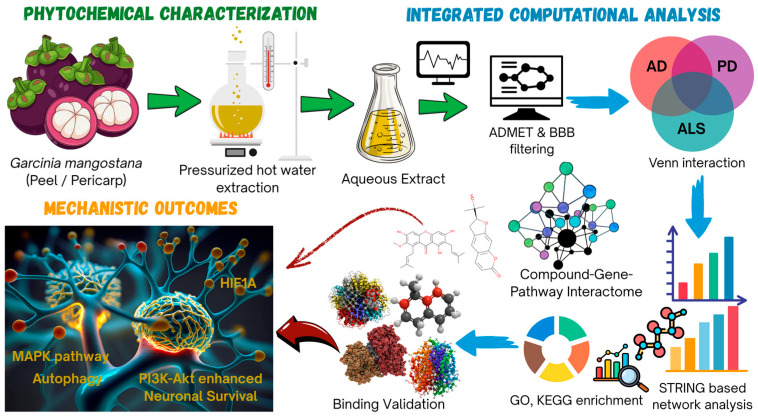
Integrated workflow illustrating the multi-target neuroprotective mechanism of *Garcinia mangostana* peel aqueous extract (GMPE). Mangosteen peel (pericarp) was subjected to pressurized hot water extraction (PHWE) to obtain a polar aqueous extract, which was chemically characterized using UHPLC–QTOF–MS. Identified metabolites were subsequently filtered through in silico ADMET and blood–brain barrier (BBB) permeability screening to prioritize central nervous system–relevant compounds. Disease-associated gene sets for Alzheimer’s disease (AD), Parkinson’s disease (PD), and amyotrophic lateral sclerosis (ALS) were integrated to identify shared molecular targets. Protein–protein interaction analysis and compound–gene–pathway network construction were performed using STRING, and functional enrichment analyses (GO and KEGG) were used to elucidate core regulatory pathways. Molecular docking was conducted to validate interactions between prioritized metabolites and hub targets. The integrated analysis highlights key signaling pathways, including PI3K–Akt, MAPK, autophagy, and hypoxia-related pathways, supporting a systems-level multi-target neuroprotective mechanism.

In this research, we aimed to explore the neuroprotective effects of the aqueous extract from *Garcinia mangostana* peel (GMPE) using an integrated methodology combining metabolomics, systems pharmacology, and molecular docking. Throughout this study, the term ‘polar neuro-interactome’ refers to the ensemble of molecular targets predicted to interact with the water-soluble polar metabolite fraction of GMPE within the context of neurodegenerative disease-associated signaling networks. To begin, the phytochemical composition of GMPE was thoroughly analyzed by UHPLC-QTOF-MS, with particular emphasis on key polar metabolites present in notable amounts. Following this, in silico ADMET and blood–brain barrier (BBB) permeability evaluations were performed to prioritize CNS-relevant compounds. Subsequently, the identified molecular targets of GMPE metabolites were cross-referenced with genes linked to AD, PD, and ALS to identify common therapeutic targets. PPI analyses and functional enrichment analyses were then performed to identify central regulatory hubs and associated signaling pathways. Ultimately, molecular docking was conducted to validate interactions between the compounds and their targets, providing evidence for the proposed multi-target neuroprotective mechanisms (Figure 1).

By shifting the focus from lipophilic xanthones to more bioavailable polar metabolites and utilizing a systems biology approach that emphasizes disease convergence, this study uncovers new insights into the neuroprotective properties of mangosteen peel. The findings not only deepen understanding of GMPE as a multi-target neuroprotective agent but also offer a reproducible methodological approach for evaluating complex botanical extracts associated with interconnected neurodegenerative diseases.

## 2. Materials and Methods

### 2.1. Chemicals and Reagents

Ultra-high-performance liquid chromatography (UHPLC)-grade acetonitrile and purified water were used for extraction and as the mobile phase. To enhance ionization efficiency, formic acid (0.1% *v*/*v*) was added to the mobile phase. Internal Reference Mass (IRM) calibration solutions were employed to ensure precise mass accuracy during the quadrupole time-of-flight (Q-TOF) analysis. All standard chemical structures and SMILES strings were sourced from the PubChem database.

### 2.2. Sample Collection and Pressurized Hot Water Extraction

Fresh fruits of *Garcinia mangostana* L. (characterized by black-purple peels) were obtained from an organic farm in Phrom Khiri, Nakhon Si Thammarat, Thailand. A voucher specimen (number 01552-4) was deposited at the Walailak University Herbarium. The peels were meticulously washed, air-dried, and ground into a fine powder.

For extraction, 100 g of the powdered peel was subjected to PHWE with distilled water at a solvent-to-solid ratio of 1:10 (*w*/*v*) in Duran glass bottles, then autoclaved at 100 °C and 15 psi (gauge pressure) for 15 min. This method enhances solute desorption while minimizing thermal exposure, improving mass transfer, and reducing degradation of bioactive compounds [20]. The resulting mixture was cooled, filtered through Whatman No. 1 paper, and centrifuged at 1500 rpm for 10 min. The supernatant was subsequently freeze-dried using an EYELA Model FDU-1200 (Tokyo, Japan) and stored in airtight containers at −20 °C.

### 2.3. UHPLC-QTOF-MS Phytochemical Elucidation

The chemical profile of GMPE was determined using a high-resolution Agilent 6200/6500 series quadrupole time-of-flight mass spectrometry (UHPLC-QTOF-MS) (Agilent Technologies, Santa Clara, CA, USA). Reconstituted GMPE (10 mg/mL in water) was vortexed, filtered through a 0.2 µm filter, and then injected with 2 µL. Chromatographic separation was performed on a Zorbax Eclipse Plus C18 column with a gradient elution of 0.1% formic acid in water (A) and acetonitrile (B). The gradient began at 100% A (0 min), shifted to 10% A and 90% B by 35 min, then returned to 100% A at 37 min, at a flow rate of 0.2 mL/min. The Agilent Jet Stream ESI source operated in both positive and negative Auto-MS/MS modes, covering an m/z range of 100–1500. Source parameters included a capillary voltage of 4000 V, a gas temperature of 350 °C, and a fragmentor voltage of 175 V. Data processing and metabolite identification were carried out using MassHunter Workstation (B.08.00) with the Metlin Metabolites PCDL library. A summary of all chromatographic separation and instrument parameters is provided in Supplementary File S1 Table S1.

### 2.4. ADMET Screening and CNS Prioritization

Metabolites identified were evaluated for drug-likeness and pharmacokinetic traits using the SwissADME server [21] (http://www.swissadme.ch/index.php), accessed on 1 February 2026. Metabolites meeting prioritization criteria are hereafter referred to as ‘bioactives’, defined as compounds selected based on quantitative abundance, predicted Blood–Brain Barrier (BBB) permeability, and drug-likeness parameters. To meet high-impact research standards, candidates were prioritized based on two criteria: quantitative abundance (Height Sum %) and BBB permeability, as shown in the BOILED-Egg plot. Only metabolites within the “Yellow Yolk” (BBB permeant) and “White” (high GI absorption) zones were selected for further systems pharmacology analysis, ensuring relevance to central nervous system (CNS) disorders. Toxicity classes and LD_50_ values were predicted with the ProTox platform [22] (https://tox.charite.de/protox3/), accessed on 1 February 2026, to exclude those with mutagenic or carcinogenic potential.

### 2.5. Systems Pharmacology and the AD-PD-ALS Interactome

To identify the shared molecular drivers of neurodegeneration, disease-associated genes for Alzheimer’s (AD), Parkinson’s (PD), and Amyotrophic Lateral Sclerosis (ALS) were retrieved from GeneCards (https://www.genecards.org/), DisGeNET (https://www.disgenet.org/), and OMIM (https://omim.org/) [23,24] accessed on 1 February 2026. For AD, PD, and ALS genes, the following keywords were used in GeneCards and OMIM: “Alzheimer’s disease”, “Parkinson’s disease”, and “Amyotrophic Lateral Sclerosis”. Entries with OMIM and GeneCards Inferred Functionality Score (GIFtS) ≥ 60 were retained to ensure high-confidence gene selection. In DisGeNET, targets associated with AD (Concept ID: C0002395), PD (Concept ID: C0030567), ALS (Concept ID: C0002736) were retrieved, and genes with a gene–disease association (GDA) score ≤ 0.4 were excluded to improve dataset reliability. A three-way Venn diagram was generated using InterActiVenn to identify the ‘core genes’ shared by all three conditions. A two-way Venn diagram was generated using a Venn diagram tool [25] (https://bioinformatics.psb.ugent.be/webtools/Venn/), accessed on 1 February 2026, to identify the “Common Core” genes shared among all three neurodegenerative conditions. The intersection between compound targets and the common disease genome defines the potential therapeutic targets. A summary of all sequential pipeline inputs, processes, and outputs is provided in Supplementary File S1 Table S2.

### 2.6. Network Analysis and Hub Gene Identification

Potential therapeutic targets were identified using the Swiss Target Prediction tool [21] (http://swisstargetprediction.ch/), accessed on 1 February 2026, with *Homo sapiens* selected as the species, based on the analysis of the SMILES strings of the most promising mangosteen compounds. A Compound–Target–Disease (CTD) network was constructed in Cytoscape 3.4.0 [26] (http://chianti.ucsd.edu/cytoscape-3.4.0/), accessed on 1 February 2026, to illustrate the polypharmacological interactions. Furthermore, a Protein–Protein Interaction (PPI) network was developed using the STRING 11.0 database [27] (https://string-db.org/), accessed on 1 February 2026, with a confidence score above 0.4. Hub genes within this network were identified and ranked by “Degree” of connectivity using the CytoHubba plugin. Target proteins were classified with the Panther Classification System [28] (https://pantherdb.org/), accessed on 1 February 2026.

### 2.7. Functional Enrichment Analysis

Gene Ontology (GO) and Kyoto Encyclopedia of Genes and Genomes (KEGG) pathway enrichment analyses for Annotation, Visualization, and Integrated Discovery were performed using ShinyGO 0.85.1 [29] (http://bioinformatics.sdstate.edu/), accessed on 1 February 2026. The analysis was conducted using *Homo sapiens* as the species, applying a False Discovery Rate (FDR) filter of <0.05 to identify key biological processes, including autophagy and the PI3K-Akt/MAPK signaling pathways. To further explore the multi-level mechanisms of plant components in treating neurodegenerative disorders, various networks, including plant-constituent, gene-pathway, constituent-target gene, and constituent-gene-pathway networks, were constructed in Cytoscape 3.4.0, accessed on 1 February 2026.

### 2.8. Prediction of Binding Affinity Between Active Components and Potential Targets of GMPE Through Molecular Docking

Molecular docking was utilized within the network pharmacology approach to confirm interactions between compounds and targets, as well as to forecast the binding modes and affinities of bioactive molecules with key protein targets, a method commonly used in modern drug discovery [30]. The three-dimensional structures of seven key ligands were sourced from the PubChem database [31] (https://pubchem.ncbi.nlm.nih.gov/), whereas the three-dimensional crystal structures of central target proteins were obtained from the Protein Data Bank (PDB) [32] (http://www.rcsb.org). The preparation of ligands and receptors was conducted using AutoDock Vina 1.2.0 [33] (https://autodock.scripps.edu/), which included energy optimization, assignment of force field parameters, elimination of extraneous atoms, and structural refinement to reach low-energy conformations. Docking simulations were carried out using the PyRx Virtual Screening platform in conjunction with AutoDock Vina. The grid center coordinates and dimensions for each protein were specified according to their specific requirements, e.g., (size x = −4.045, y = −13.44, z = 35.207) for (PDB: 4EY7) grid spacing of 1.0 Å, and dimensions of 72 × 110 × 80 points to sufficiently cover the active pocket, (size x = 18.808, y = 0.896, z = 15.802) for (PDB: 3QKK), (size x = 44.07, y = 47.262, z = −0.548) for (PDB: 1W51), (size x = 29.255, y = 24.952, z = 0.769) for (PDB: 2W3L), (size x = 58.358, y = 5.585, z = 18.577) for (PDB: 1JPW), (size x = 9.177, y = 5.585, z = 40.981) for (PDB: 1M17), (size x = 29.255, y = 6.362, z = 36.908) for (PDB: 1PYX), and (size x = −16.311, y = 46.837, z = 30.13) for (PDB: 2AZ5). Ligands and targets were docked to assess binding affinity, shedding light on potential interactions between GMPE compounds and AD, PD, and ALS-related proteins. Binding affinities were assessed based on computed binding energies (kcal/mol), with lower values indicating stronger, more stable interactions between ligands and receptors. The best docking poses were chosen based on binding energy and interaction geometry, and molecular interactions were visualized with Discovery Studio Visualizer 2020.

## 3. Results

### 3.1. Phytochemical Characterization of GMPE via UHPLC-QTOF-MS

**Figure 2:**
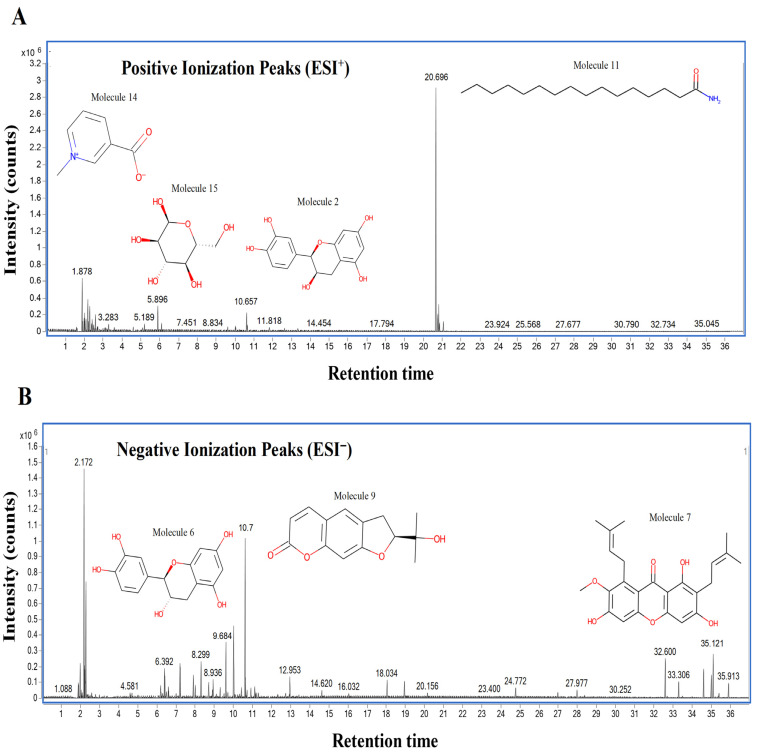
UHPLC-QTOF-MS base peak chromatograms of *G. mangostana* peel aqueous extract (GMPE) in (**A**) positive and (**B**) negative electrospray ionization modes show the varied distribution of polar metabolites over retention times.

The chemical profile of the GMPE was thoroughly characterized using high-resolution UHPLC-QTOF-MS in both negative and positive electrospray ionization (ESI) modes. The total ion chromatograms (TICs) showed a complex array of bioactive compounds, characterized by high-intensity peaks across a broad retention-time range (Figure 2) (Supplementary File S2). In positive mode (ESI^+^), the extract was dominated by Palmitic amide, which accounted for 18.61% of the combined area at RT 20.696 min (Table 1). Other notable polar metabolites included 6-(alpha-D-glucosaminyl)-1D-myo-inositol (15.29%), Trigonelline (4.79%), and Choline (3.88%) (Table 1). The negative mode (ESI^−^) provided complementary insights, identifying Quinic acid as the major component with an area of 16.38% at RT 2.305 min. Importantly, despite the extract’s aqueous nature, key xanthones and flavonoids such as α-Mangostin (2.61%), Epicatechin (6.32%), Procyanidin B2 (5.06%), and Marmesin (1.35%) were detected, revealing the diversity of polar and mid-polar markers that confirm PHWE’s capacity to extract bio-accessible fractions.

### 3.2. Strategic Bioavailability Screening and ADMET Profiling

**Figure 3:**
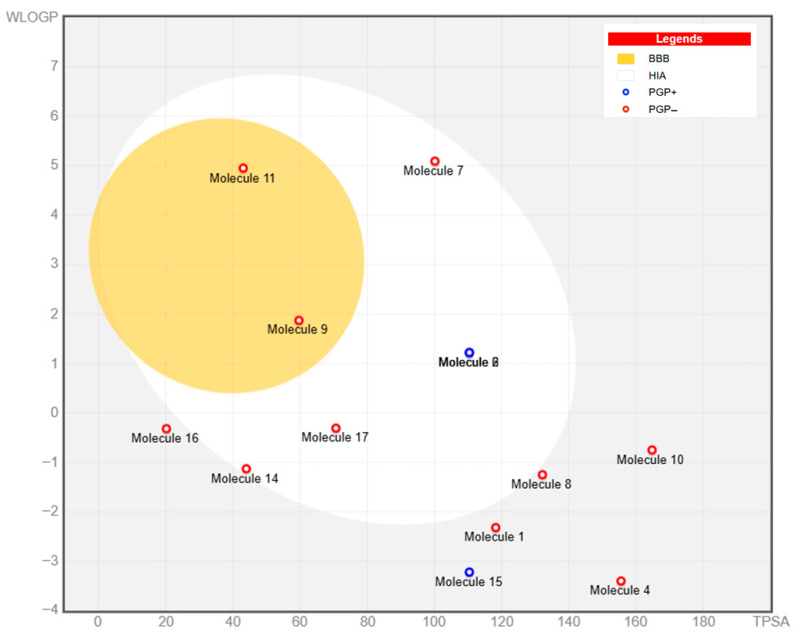
BOILED-Egg model illustrating predicted gastrointestinal absorption and blood–brain barrier permeability of GMPE metabolites, with classification of P-glycoprotein substrates and non-substrates.

To ensure translational relevance for CNS therapeutic applications, the identified metabolites were systematically assessed using SwissADME-based pharmacokinetic filtering. The BOILED-Egg model was utilized as a key strategic screening tool to forecast gastrointestinal (GI) absorption and blood–brain barrier (BBB) permeability (Figure 3) among the 17 highest-ranked metabolites (Table 2) (Supplementary File S3). Compound selection followed a prioritization framework. Primary CNS leads comprised metabolites falling within the ‘Yellow Yolk’ zone (BBB = Yes) with high GI absorption and zero Lipinski violations (Palmitic amide or Molecule 11 and Marmesin or Molecule 9). Several compounds that exhibited high GI absorption but limited BBB penetration were retained as secondary benchmarks for molecular docking, particularly against enzymatic targets like acetylcholinesterase (AChE) [34]. Secondary candidates were retained from the ‘White’ zone (high GI absorption, BBB = No) exclusively when supported by independent experimental evidence of neuroactive properties in the published literature or by high quantitative abundance in GMPE (>3% area sum), including α-Mangostin, Epicatechin, (±)-Catechin, Trigonelline, and Kojic acid. α-D-Glucose was included as an endogenous CNS reference compound, as its BBB permeation occurs via active GLUT1/GLUT3 transport, not captured by passive diffusion-based computational models. Compounds outside both zones or with ≥2 Lipinski violations were excluded from further analysis. Full compound selection criteria, prioritization rationale, and exclusion list are provided in Supplementary File S3.

Toxicological risk assessment conducted using the ProTox-II platform categorized the prioritized BBB-permeable leads, Palmitic amide and Marmesin, as toxicity Class IV (predicted LD_50_ ≈ 1000 mg/kg), indicating a favorable safety margin for further investigation (Table 3). In summary, this comprehensive multi-layered ADMET filtering approach effectively reduced the chemical space to CNS-relevant, bioavailable, and low-toxicity metabolites, establishing a solid groundwork for subsequent network pharmacology and molecular docking analyses.

### 3.3. Identification of the AD-PD-ALS Pathological Interactome

**Figure 4:**
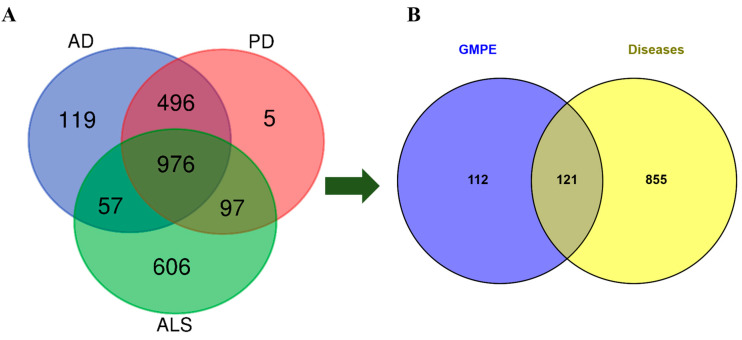
Venn diagram analysis showing overlapping gene targets associated with neurodegenerative diseases, including (**A**) shared disease-related genes among Alzheimer’s disease (AD), Parkinson’s disease (PD), and amyotrophic lateral sclerosis (ALS), and (**B**) intersection between GMPE-predicted targets and common AD-PD-ALS-associated genes.

Systems pharmacology was utilized to delineate the interconnected genetic links among AD, PD, and ALS. A Venn diagram illustrating the three diseases uncovered a significant common pathological core comprising 976 genes shared by all three conditions (Figure 4A). To identify targets related to AD, PD, and ALS, we gathered targets from the GeneCards, DisGeNET, and OMIM databases. The genes associated with AD, PD, and ALS were obtained after removing duplicates, and we selected proteins present in all databases (Supplementary File S4).

To assess the potential therapeutic applications of the extract, the combined predicted targets of the prioritized GMPE bioactives were compared with the disease core. This analysis identified 121 prospective therapeutic targets through which GMPE may influence the shared mechanisms underlying neurodegeneration (Figure 4B, Supplementary File S1 Table S3).

### 3.4. PPI Network Topology, Hub Gene Identification, and Protein Classification

To clarify biological communication and the centralized signaling hubs within the common AD-PD-ALS interactome, a PPI network was constructed using the STRING 11.0 database. The network was built using 121 potential therapeutic targets and refined with a highest confidence threshold of 0.900 to balance sensitivity and specificity in depicting protein interactions.

#### 3.4.1. Topological Analysis of the Disease Nexus

**Figure 5:**
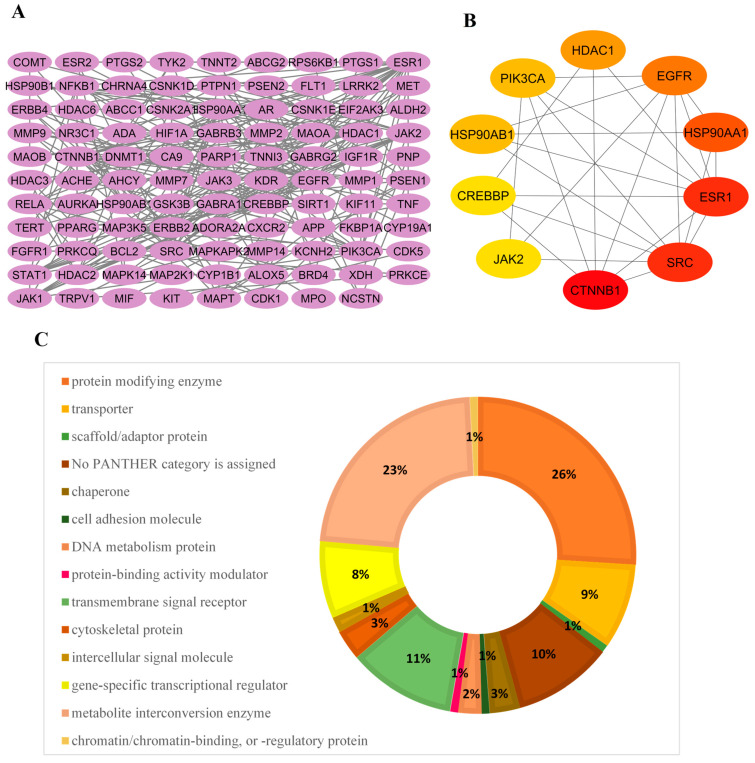
(**A**) Topological analysis of the protein–protein interaction (PPI) network of shared AD-PD-ALS target genes, (**B**) Top 10 hub genes ranked by degree centrality, and (**C**) Functional classification of Neuro diseases (AD, PD, ALS) genes and GMPE-associated targets based on the Panther Classification System.

The resulting PPI network included 121 nodes and 250 edges, with each node representing a GMPE-associated therapeutic target and each edge indicating a high-confidence protein–protein interaction. A connected interaction network comprising 98 proteins and 250 edges was established; the remaining 23 proteins were excluded from topological analysis because they were isolated nodes lacking high-confidence interaction partners within the target set (Figure 5A). The average node degree was 4.13, suggesting that each target protein interacts with about four others, indicative of a moderately connected interactome. The average local clustering coefficient was 0.487, indicating that target proteins tend to form interconnected clusters, reflecting functional modularity. PPI enrichment analysis yielded a *p*-value of <1.0 × 10^−16^, indicating that these target proteins have significantly more interactions than expected in a random protein set, supporting their meaningful functional interconnectedness.

#### 3.4.2. Identification of Central Hub Genes

Using the CytoHubba plugin, we ranked the 10 most similar target proteins in the PPI network based on their topological scores [35]. All 11 algorithms were used together: MCC, DMNC, MNC, Degree, EPC, BottleNeck, Eccentricity, Closeness, Radiality, Betweenness, and Stress (Supplementary File S1 Table S4). The top 10 hub proteins, ranked by degree, were identified (Figure 5B). These proteins are believed to provide critical pathways for biological signals within the NDD nexus.

CTNNB1, the main effector of Wnt/β-catenin signaling, had the highest centrality scores across multiple metrics (Degree: 22) and was found to be suppressed in AD, PD, and ALS [36,37]. SRC also ranked second overall (top-3 in MCC: 1323; Degree: 21) and was identified as a key mediator of neuroinflammation and synaptic signaling [38]. ESR1 exhibited the most central values, with MNC (21) and EPC (26.81), indicating the neuroprotective role of estrogen receptor-α by mediating inflammatory responses, mitochondrial function, and the clearance of amyloid precursors [39]. EGFR also showed a significant centrality value of 1560 in MCC, suggesting it forms denser and wider protein communities that support neuronal survival during neuroinflammatory processes [40]. Additionally, HSP90AA1 and HSP90AB1 ranked fourth and eighth, respectively, with the lowest eccentricity (0.15136), highlighting their roles as key chaperones that stabilize client proteins under proteotoxic stress and in the pathological accumulation of hyperphosphorylated tau protein [41]. Furthermore, HDAC1 had Degree (16) and Betweenness (561) scores, suggesting a role in the epigenetic regulation of tau, SOD1, and α-synuclein acetylation; studies suggest that inhibiting HDAC1 may offer neuroprotection [42]. PIK3CA showed DMNC (0.524) and MCC (1293) scores, indicating its vital role in driving the PI3K-Akt/mTOR-autophagy pathway that maintains proteostasis. CREBBP had MCC (370) and EPC (23.94) scores, demonstrating its early neurogenic coactivator activity for CREB-regulated neurotrophic genes and its role in catalyzing tau acetylation [43]. Lastly, JAK2 exhibited the highest DMNC (0.541) and clustering (0.474) scores, underscoring its key role in IL-6/IFN-γ-mediated glial neuroinflammatory processes [44,45].

#### 3.4.3. Functional Protein Classification

The 121 identified target genes were subsequently classified using the PANTHER (Protein Analysis Through Evolutionary Relationships) Classification System to analyze the distribution of protein classes involved in the extract’s mechanism (Figure 5C). The functional profile showed a predominance of Protein-modifying enzymes (26%) and Metabolite-interconversion enzymes (23%), which together account for nearly half of the therapeutic target spectrum. This underscores the extract’s capability to influence post-translational modifications and maintain metabolic balance in neurons. Other notable classes included Transmembrane signal receptors (11%), Transporters (9%), and Gene-specific transcriptional regulators (8%). The diversity of classes, such as Chaperones (3%) and Cytoskeletal proteins (3%), highlights the comprehensive synergistic strategy of GMPE bioactives in restoring cellular proteostasis and structural integrity in AD, PD, and ALS.

### 3.5. Functional Enrichment and Pathway Modulation Analysis

To unravel the complex biological mechanisms underlying the neuroprotective effects of the 121 core targets, we performed detailed Gene Ontology (GO) and Kyoto Encyclopedia of Genes and Genomes (KEGG) enrichment analyses. This comprehensive approach connects the phytochemical characteristics of GMPE with its physiological influence on the AD-PD-ALS nexus.

#### 3.5.1. GO Enrichment

**Figure 6:**
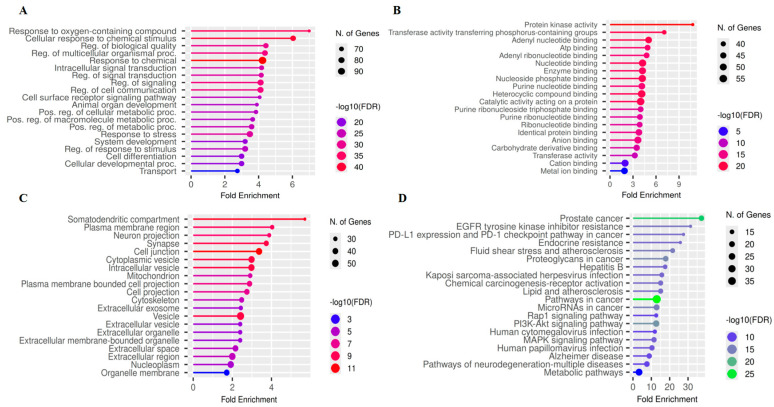
The enrichment analysis chart of the top 20 Gene Ontology (GO) categories for (**A**) biological processes (BP), (**B**) molecular functions (MF), (**C**) cellular component (CC), and (**D**) Kyoto Encyclopedia of Genes and Genomes (KEGG) of Neuro diseases (AD, PD, ALS) genes and the active components of *G. mangostana*.

The results of the biological process (BP) (Figure 6A) enrichment clearly aligned with the known antioxidant and neuro-modulatory effects of the extract. The most prominent categories identified were “Response to oxygen-containing compound,” “Cellular response to chemical stimulus,” and “Response to stress,” highlighting the extract’s potential to reduce oxidative damage, a major factor in motor neuron degeneration in ALS and in the loss of dopaminergic neurons in PD [46]. Analysis of molecular function (MF) (Figure 6B) revealed a high concentration of targets associated with Protein kinase activity and ATP binding, suggesting that the bioactive compounds in GMPE primarily function as signaling modulators rather than as enzyme inhibitors. Additionally, the enrichment analysis for cellular components (CC) (Figure 6C) specifically pinpointed these targets within the somatodendritic compartment, synapses, and neuronal projections, thereby confirming the extract’s targeting of the CNS structure.

#### 3.5.2. KEGG Pathway Analysis

The KEGG enrichment analysis identified the 20 pathways most strongly influenced by the GMPE-target interactome (Supplementary File S1 Table S5). The pathway with the highest statistical significance was “Pathways in cancer” (hsa05200) (FDR = 1.02 × 10^−27^), which encompasses 36 common genes, including BCL2, EGFR, and JAK2. In the realm of neurodegeneration, this enrichment highlights a significant alteration in cell survival and apoptotic processes that are shared between oncogenic and neurotoxic environments [47].

**Figure 7:**
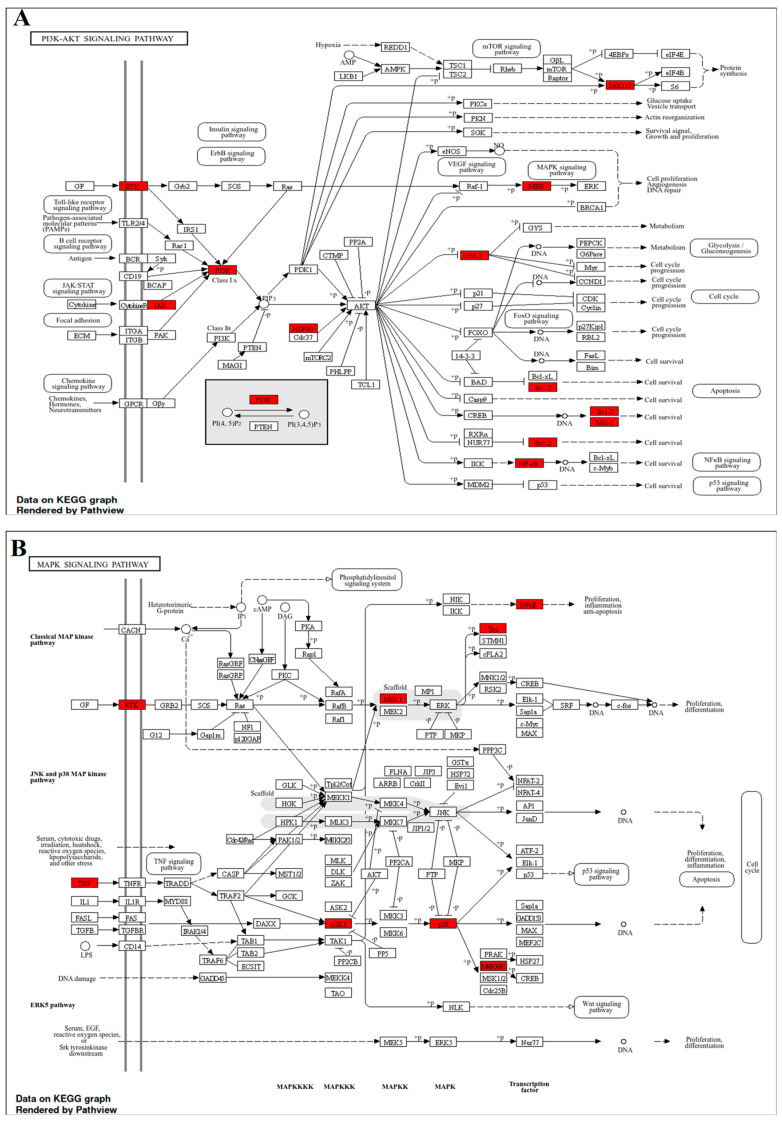
Key enriched KEGG signaling pathways associated with GMPE targets, including (**A**) PI3K-Akt signaling pathway (hsa04151), and (**B**) MAPK signaling pathway (hsa04010), illustrating molecular nodes involved in neuronal survival, inflammation, and stress response. Pathway diagrams were reproduced according to KEGG guidelines [52].

Among the 20 most significantly enriched pathways with the highest degree of overlap of GMPE-related targets (i.e., the PI3K–Akt signaling pathway; 24 target genes; FDR = 7.06 × 10^−18^) among the neuronal survival/homeostasis pathways by inhibiting apoptosis and tau phosphorylation in AD [48]. In PD and ALS, it counters oxidative stress, GSK-3β activation, and dopaminergic/motor neuron death [17]. The PI3K–Akt pathway is therefore the top-ranking pathway related to neuronal survival/homeostasis (key regulators: AKT1, BCL2, and PIK3CA) (Figure 7A). The second-highest-ranking pathway is the MAPK signaling pathway (18 target genes; FDR = 4.61 × 10^−13^), which regulates critical aspects of neuronal function (e.g., neuroinflammation, stress response, and apoptosis) [49] through the genes TNF, EGFR, and JAK2 (Figure 7B). MAPK drives apoptosis, tau/APP phosphorylation, and neuroinflammation in AD and PD [50], and in ALS, it links stress responses to motor neuron/glial defects and progression [51]. The identification of “Pathways of neurodegeneration-multiple diseases” (hsa05022) (FDR = 7.88 × 10^−11^) and “Alzheimer disease” (hsa05010) (FDR = 2.50 × 10^−11^) as leading categories offers direct genetic evidence for the extract’s multi-target effectiveness across the neurodegenerative disease triad.

#### 3.5.3. The Compound–Gene–Pathway (CGP) Network

**Figure 8:**
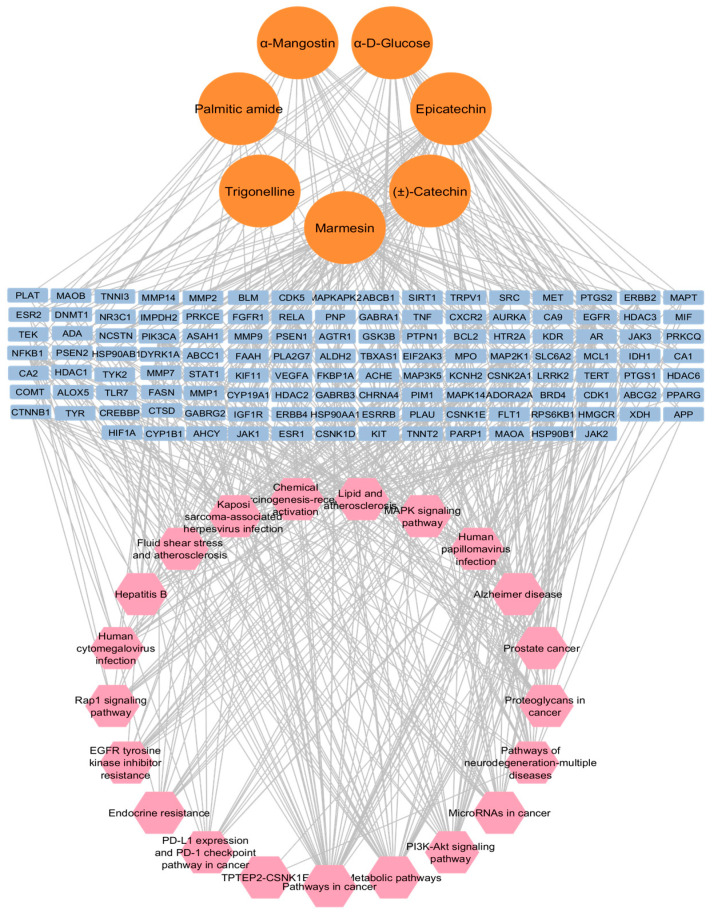
Integrated compound–gene–pathway network of GMPE, showing multi-component, multi-target, and multi-pathway interactions, bioactive compounds (orange), target genes (blue), and enriched signaling pathways (pink).

The resulting compound–gene–pathway network (Figure 8) depicts a well-integrated mechanism in which individual metabolites, such as Marmesin and α-Mangostin, interact with multiple hubs within the NDD interactome. The network construction statistics revealed an interactome comprising 152 nodes and 567 edges, with an average neighbor count of 7.461. The elevated average connectivity suggests that the aqueous extract does not depend on a single “silver bullet” molecule but rather uses a collaborative poly-metabolite approach. In this network, specific metabolites are shown to interact simultaneously with several hub genes (e.g., CTNNB1, EGFR), which are themselves part of multiple pathological pathways. This extensive overlap clarifies the enhanced effectiveness of the aqueous fraction in addressing the intricate, multifactorial relationship of the AD-PD-ALS nexus compared to individual compounds.

### 3.6. Molecular Docking Validation Against Neuro-Hubs

**Figure 9:**
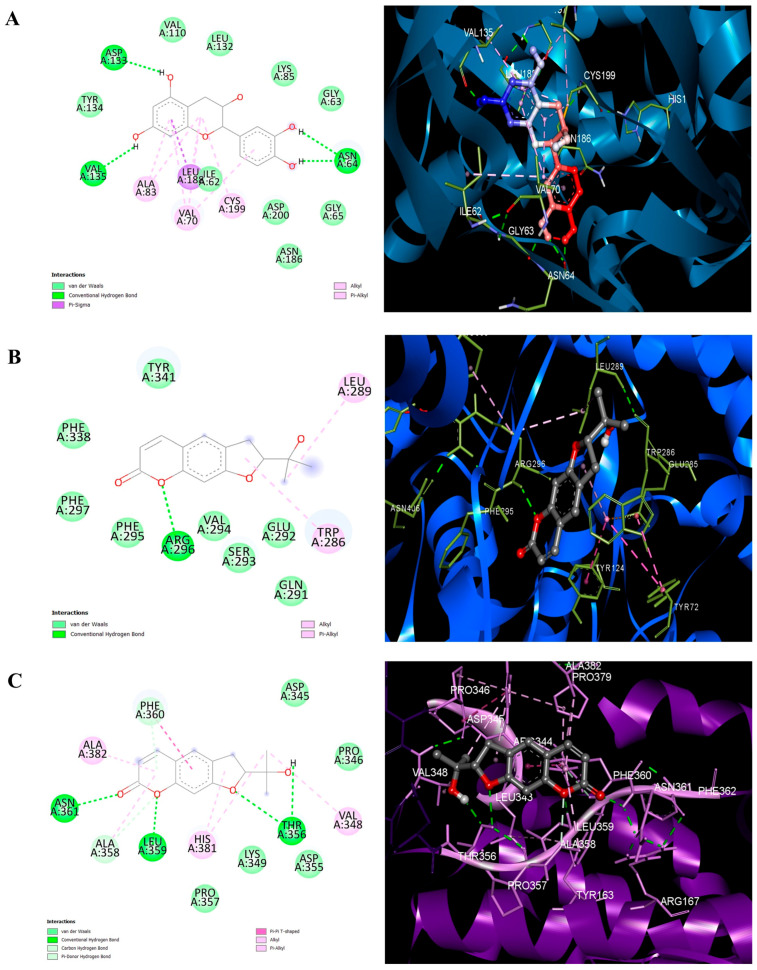
2D and 3D interaction diagrams of (**A**) Epicathechin in the active site of Hypoxia Inducible Factor 1 Alpha (PDB ID 1PYX), (**B**) Marmesin in the active site of Acetylcholinesterase (PDB ID 4EY7), and (**C**) Marmesin in the active site of Hypoxia Inducible Factor 1 Alpha (PDB ID 1PYX), highlighting hydrogen bonding and hydrophobic interactions stabilizing ligand binding.

Binding affinities (ΔG) for lead bioactives against the identified hub proteins were assessed to validate systems pharmacology predictions (Table 4). The key proteins were selected based on their neuroactivity. These included AChE, AKT, APP, BCL2, CTNNB1, EGFR, HIF1A, and TNF, which are known to be involved in neuroinflammation, response to oxidative stress, and neuronal signaling pathways associated with neurodegenerative diseases [53,54,55]. The docking analysis results showed that the selected compounds (e.g., marmesin, α-mangostin) have binding energies ranging from −4.06 to −6.89 kcal/mol. Marmesin had the lowest binding energy (−6.89 kcal/mol) with HIF1A, followed by AChE and TNF, which had binding energies of −5.79 kcal/mol and −5.47 kcal/mol, respectively. α-Mangostin bound to AChE with a binding energy of −5.17 kcal/mol. Based on the observed binding energies, the predicted interactions between GMPE bioactives and the selected protein targets are expected to be weak to moderate. The docking pose analysis showed that the main interactions holding the molecules in place were hydrogen bonds and hydrophobic interactions between the ligands and the protein’s active or allosteric sites. The observed interaction patterns suggest that GMPE-derived metabolites may affect the functions of target proteins. We converted the binding free energies into estimated dissociation constants (Kd, µM) using the following formula: Kd = e^(ΔG/RT)^ at T = 298 K. Similarly, like binding affinities, Marmesin had the best predicted affinities for all four targets and produced Kd values of 9.80 µM (HIF1A), 18.90 µM (AKT1), 57.40 µM (AChe), and 91.20 µM (APP), all falling below the standard threshold of 100 µM used to gauge the validity of the results obtained in computational screening studies. Epicatechin also showed an excellent predicted affinity for HIF1A, yielding a Kd of 68.40 µM. Palmitic amide, on the other hand, was predicted to have uniformly poor affinities for all four targets, yielding a range of Kd values (572–66,800 µM), which indicates that this compound will likely have its neuroprotective contributions through means other than direct protein binding, such as membrane modulation or lipid signaling pathways. The 2D and 3D interaction diagrams (Figure 9) illustrate that these compounds stabilize the active sites of their targets via multiple conventional hydrogen bonds and hydrophobic interactions (e.g., pi-alkyl and van der Waals forces), providing structural evidence for the neuroprotective mechanism of the aqueous extract. The molecular docking results indicate preliminary evidence of interactions with key targets linked to neurodegenerative diseases, but moderate binding affinities highlight the need for further validation through dynamic simulations or experimental methods.

## 4. Discussion

Previous studies on mangosteen have primarily focused on lipophilic xanthones, particularly α- and γ-mangostin, which are extracted using organic solvents [10]. Although these compounds exhibit strong in vitro antioxidant and anti-inflammatory properties, their practical application is limited by poor water solubility, low oral bioavailability, questionable permeability across the BBB, and the risk of dose-dependent toxicity [56]. Additionally, many previous studies have focused on isolated compounds and single-disease models, thereby limiting their relevance to the intricate, network-based nature of neurodegeneration [57]. In contrast, our research intentionally redirects attention to polar, water-soluble metabolites that are concentrated through PHWE. Analysis via UHPLC–QTOF–MS showed that GMPE is primarily composed of quinic acid and palmitic amide, along with epicatechin, marmesin, and trigonelline. The significant presence of these metabolites supports a poly-metabolite approach, where several moderately active compounds together influence pathways relevant to disease. This strategy is especially appropriate for chronic neurodegenerative conditions, where long-term safety and network-level modulation are vital.

One of the primary challenges in developing neurotherapeutics is ensuring effective delivery to the central nervous system. In this respect, our targeted use of SwissADME-based filtering, along with the BOILED-Egg model, enabled us to prioritize metabolites logically accessible to the CNS. Palmitic amide and marmesin emerged as important candidates, offering a combination of high gastrointestinal absorption, predicted blood–brain barrier permeability, and low toxicity [14]. Notably, palmitic amide was identified as the most prevalent metabolite, underscoring its relevance for translational applications. Conversely, abundant compounds such as quinic acid and procyanidin B2 were deprioritized for CNS-focused studies, despite their beneficial antioxidant properties, because of their low predicted blood–brain barrier permeability [14]. This strategic filtering approach enhances biological relevance and distinguishes this study from general phytochemical analyses that do not account for pharmacokinetic factors.

The convergence of GMPE-predicted targets with genes associated with AD, PD, and ALS identified 121 common targets, indicating significant molecular overlap across these conditions. PPI network analysis highlighted CTNNB1, SRC, ESR1, HSP90AA1, EGFR, HDAC1, PIK3CA, HSP90AB1, CREBBP, and JAK2 as key hub genes, representing important pathological axes related to neuroinflammation, neuronal survival, protein aggregation, and oxidative stress [54]. The discovery of these hubs supports the idea that neurodegeneration is influenced by interconnected molecular networks, rather than solely by disease-specific factors. Notably, these targets are not just indicators of pathology but also actively participate in disease progression, making them particularly appealing for multi-target modulation with botanical extracts.

Functional enrichment analysis further validated the overarching effects of GMPE. The enrichment of the PI3K–Akt signaling pathway underscores its role in promoting neuronal survival, maintaining synaptic integrity, and enhancing resistance to oxidative stress [17]. Additionally, the concurrent enrichment of the MAPK pathway, typically linked to inflammatory and apoptotic signaling, suggests that GMPE metabolites may help balance pro-survival and pro-death pathways [18]. Furthermore, pathways associated with autophagy and metabolic regulation were notably represented, which corresponds with emerging treatment strategies focused on improving the clearance of misfolded proteins, such as amyloid-β and α-synuclein [15]. Together, these results support a neuroprotective model that involves multiple mechanisms rather than relying on a single pathway hypothesis.

Molecular docking analysis was used to explore the neuroprotective potential of key metabolites from GMPE by examining their interactions with hub targets (CTNNB1 and EGFR) and other proteins involved in neurodegeneration (AChE, AKT1, APP, BCL2, HIF1A, and TNF). AChE is a suitable therapeutic target for treating the symptoms of AD [34]. An important role for amyloid-beta formation is through the intermediate influence of APP [58]. The critical regulators of the PI3K-Akt signaling pathway needed for neuronal survival and death, respectively, are AKT1 and PIK3CA [48]. Interactions with TNF and EGFR suggest roles in modulating neuroinflammatory and survival pathways, while binding to BCL2 and AKT1 supports anti-apoptotic mechanisms [59,60]. BCL2 regulates cell death through apoptosis; CTNNB1 and HIF1A play important roles in Wnt signaling, mediating the plasticity of synapses [37,54]. Favorable interactions with AChE and APP underline the wide-ranging neuroprotective effects of GMPE [34]. The metabolites showed moderate binding affinities and stable hydrogen-bond interactions with key amino acids, characteristic of small, polar, naturally occurring metabolites that target multiple proteins rather than a single high-affinity protein. By including both network-derived proteins identified as hubs and well-known disease-related targets, it is possible to evaluate the potential for pharmacological interactions more thoroughly, integrating data-driven network analysis with relevant biological knowledge.

To highlight the originality of our discoveries, we systematically compared GMPE with previously documented mangosteen extracts utilized in neurodegenerative studies [Table 5]. This comparison underscores essential differences in the composition of extracts, the methodological strategies used, and the translational significance.

The current investigation has been deliberately structured as a computation-first approach, inspired by metabolomic data validated through experimentation. This strategy tackles a significant challenge in natural product research: the substantial expenses, ethical concerns, and low success rates of early in vivo experiments. Using systems pharmacology and molecular docking, we have identified CNS-relevant candidates that are bioavailable and exhibit low toxicity, before progressing to biological models. This methodology is increasingly recognized as a crucial step before conducting animal and cellular studies, especially for complex botanical extracts containing numerous bioactive components. Crucially, the computational framework used here does not substitute for experimental validation but rather enhances and rationalizes it, ensuring that subsequent in vivo research is driven by hypotheses and is resource efficient. The combination of experimentally validated metabolomics with systems pharmacology, prioritization of compounds based on bioavailability and blood–brain barrier guidance, and a disease-convergence framework covering AD, PD, and ALS are all key components of our study. A quantitative focus on abundant polar metabolites, along with a green extraction method with translational significance, is also emphasized.

## 5. Limitations of the Study

This research has several limitations. The mechanistic framework based on computational predictions (target identification, pathway enrichment, molecular docking) implies neuroprotective activity of GMPE but needs in vitro or in vivo validation. Future studies will test these predicted mechanisms using neuronal cell line models and validate findings in animal models of neurodegeneration. Additionally, ADMET predictions using SwissADME and ProTox-II may not accurately reflect in vivo pharmacokinetics, as they do not account for factors such as active transport and plasma protein binding, which can affect CNS exposure. Future research will involve in vitro assessments of BBB penetration and improved molecular docking through dynamics simulations and free energy calculations. Network pharmacology will also explore potential interactions among GMPE metabolites, followed by experimental validation of target proteins and pharmacokinetics. Overall, this systematic approach aims to direct experimental efforts in future studies efficiently.

## 6. Conclusions

This research utilized an integrated system of pharmacological and metabolomics approaches to investigate possible interactions between the polar metabolite components of the GMPE and common molecular targets implicated in AD, PD, and ALS. The results indicate that GMPE-derived biological activity may involve multiple target interactions that mediate neuron survival, oxidative stress, and neuroinflammation, including the PI3K-Akt and MAPK signaling pathways. The results of the present study (computationally based predictive modeling with network analysis) do not demonstrate direct, causal biological relationships regarding the effectiveness of GMPE-derived biological activity or the putative compounds identified in this analysis in eliciting such activity. Consequently, the results presented here should be considered only as a hypothesis-generating framework for future experimental studies. Therefore, validation using in vitro and/or in vivo experimental methodologies is needed to determine whether GMPE-derived phytochemicals have neuroprotective properties and, if so, which mechanisms are predominant.

## Figures and Tables

**Table 1:** Top ten abundant positive (ESI^+^) and negative (ESI^−^) polar metabolites in GMPE identified by UHPLC–QTOF–MS.

Retention Time (min)	Putative Identity	Formula	Observed *m*/*z*	Theoretical *m*/*z*	Mass Error (ppm)	Area Sum (%)
Positive (ESI^+^) modes						
20.696	Palmitic amide	C_16_H_33_NO	256.2633	255.2562	0.61	18.61%
1.947	6-(alpha-D-glucosaminyl)-1D-myo-inositol	C_12_H_23_NO_10_	342.1394	341.1322	0.28	15.29%
4.508	D-galactopyranosyl-(1-3)-L-arabinose	C_17_H_30_O_15_	497.1468	474.1585	1.89	9.66%
10.657	Epicatechin	C_15_H_14_O_6_	291.0866	290.079	−0.71	6.47%
10.029	Procyanidin B2	C_30_H_26_O_12_	579.1502	578.1424	−0.33	5.38%
2.199	Trigonelline	C_7_H_8_NO_2_	138.0552	138.0555	−2.07	4.79%
2.023	*α*-D-Glucose	C_6_H_12_O_6_	203.0525	180.0634	−0.56	4.20%
1.898	Choline	C_5_H_14_NO	104.1073	104.1075	−2.67	3.88%
5.135	Kojic Acid	C_6_H_6_O_4_	143.0338	142.0266	0.93	2.73%
2.5	Sucrose	C_12_H_22_O_11_	365.1056	342.1162	−0.16	1.92%
Negative (ESI^−^) modes						
2.305	Quinic acid	C_7_H_12_O_6_	191.0563	192.0634	−1.01	16.38%
10.675	Epicatechin	C_15_H_14_O_6_	289.0721	290.079	−1.09	6.32%
10.073	Procyanidin B2	C_30_H_26_O_12_	577.1349	578.1424	0.47	5.06%
1.916	Galactaric acid	C_6_H_10_O_8_	209.0376	210.0376	−0.06	4.51%
2.618	Sucrose	C_12_H_22_ O_11_	341.1089	342.1162	0.4	3.96%
9.684	(±)-Catechin	C_15_H_14_O_6_	289.0718	290.079	−0.24	3.28%
35.121	*α*-Mangostin	C_24_H_26_O_6_	409.1657	410.1729	0.03	2.61%
4.626	Citric acid	C_6_H_8_O_7_	191.02	192.027	−1.03	1.88%
10.7	Marmesin	C_14_H_14_O_4_	245.0818	246.0892	0.37	1.35%
9.345	Chlorogenic Acid	C_16_H_18_O_9_	353.0875	354.0951	0.93	0.64%

**Table 2:** ADMET and drug-likeness profiling of identified GMPE metabolites.

Molecule	Metabolites	MW (g/mol)	HBA	HBD	GI	BBB	Pgp	BA	Lipinski’s Violations
1	Quinic acid	192.17	6	5	Low	No	No	0.56	0
2	Epicatechin	290.27	6	5	High	No	Yes	0.55	0
3	Procyanidin B2	578.52	12	10	Low	No	No	0.17	3
4	Galactaric acid	210.14	8	6	Low	No	No	0.11	1
5	Sucrose	342.30	11	8	Low	No	Yes	0.17	2
6	(±)-Catechin	290.27	6	5	High	No	Yes	0.55	0
7	α-Mangostin	410.46	6	3	High	No	No	0.55	0
8	Citric acid	192.12	7	4	Low	No	No	0.56	0
9	Marmesin	246.26	4	1	High	Yes	No	0.55	0
10	Chlorogenic Acid	354.31	9	6	Low	No	No	0.11	1
11	Palmitic amide	255.44	1	1	High	Yes	No	0.55	0
12	6-(alpha-D-Glucosaminyl)-1D-myo-inositol	341.31	11	9	Low	No	Yes	0.17	2
13	D-Galactopyranosyl-(1-3)-D-galactopyranosyl-(1-3)-L-arabinose	474.41	15	10	Low	No	Yes	0.17	2
14	Trigonelline	137.14	2	0	High	No	No	0.55	0
15	α-D-Glucose	180.16	4	2	High	No	No	0.55	0
16	Choline	104.17	1	1	Low	No	No	0.55	0
17	Kojic Acid	142.11	4	2	High	No	No	0.55	0

Note: Molecular weight (MW), hydrogen bond acceptors (HBA), hydrogen bond donors (HBD), gastrointestinal (GI) absorption, blood–brain barrier (BBB) permeability, P-glycoprotein (P-gp) substrate status, and bioavailability score (BA).

**Table 3:** Toxicity classification and acute oral toxicity (LD_50_) prediction of selected GMPE metabolites.

Structure	Molecule Name	Toxicity (Class)	LD_50_ (mg/kg)
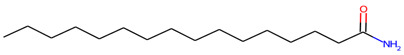	Palmitic amide	IV	1000
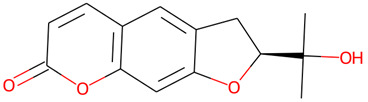	Marmesin	IV	1000
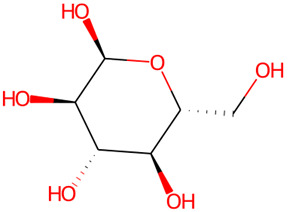	α-D-Glucose	VI	23,000
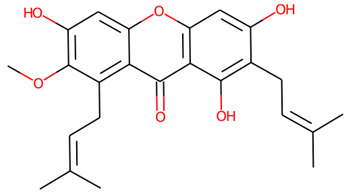	α-Mangostin	IV	1500
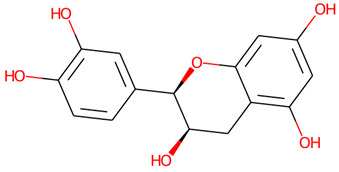	Epicatechin	VI	10,000
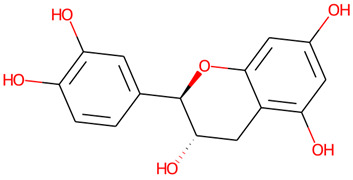	(±)-Catechin	VI	10,000
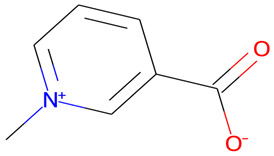	Trigonelline	V	3720

**Table 4:** Molecular docking binding affinities (kcal/mol) of selected GMPE metabolites against core neurodegenerative targets, including AChE, AKT, APP, BCL2, CTNNB1, EGFR, HIF1A, and TNF. More negative values indicate stronger predicted binding interactions.

Target [PDB]	Compound	Binding Energy (Kcal/mol)	Kd (µM)	Key Interactions and Contact Distances (Length Å)
Acetylcholinesterase (AChE) [4EY7]	Palmitic amide	−4.29	572.3	-
	Marmesin	−5.79	57.4	ARG-296 (2.35)
	α-D-Glucose	−3.67	1820	GLN-527 (4.09), ARG-522 (5.59, 6.21), TYR-510 (6.23), GLY-523 (3.17, 4.61)
	α-Mangostin	−5.17	148.2	THR-383 (4.27)
	Epicatechin	−4.41	484.7	SER-293 (2.59), TYR-341 (4.57), PHE-295 (5.24)
	(±)-Catechin	−3.87	1280	GLY-523 (4.25), ARG-525 (3.68), ARG-522 (5.78)
	Trigonelline	−3.84	1340	HIS-405 (3.48)
AKT serine/threonine kinase 1 (AKT1) [3QKK]	Palmitic amide	−3.84	1340	GLY-294 (3.32), ASP-292 (3.73)
	Marmesin	−6.52	18.9	PHE-472 (5.68), GLN-218 (4.04)
	α-D-Glucose	−4.55	537.0	PHE-472 (5.39), GLN-218 (3.58, 3.79), VAL-145 (3.82)
	α-Mangostin	−5.31	131.6	PHE-472 (6.42), GLN-218 (3.49, 3.94), VAL-145 (3.89)
	Epicatechin	−5.41	109.6	ARG-346 (5.16), TYR-340 (6.35), ARG-367 (2.72, 4.05)
	(±)-Catechin	−5.10	183.2	TYR-474 (5.23), SER-216 (4.40), GLU-459 (4.50, 4.61)
	Trigonelline	−5.30	133.8	PHE-368 (3.40), LYS-377 (4.88)
Amyloid Precursor Protein (APP) [1W51]	Palmitic amide	−2.17	55,200	LEU-306 (5.70, 5.81)
	Marmesin	−5.51	91.2	GLN-73 (4.35), THR-72 (3.94)
	α-D-Glucose	−2.25	44,800	SER-187 (4.99), LEU-188 (5.46, 5.55), TYR-190 (4.24), GLU-290 (3.61, 4.54)
	α-Mangostin	−3.28	3560	GLU-255 (3.72)
	Epicatechin	−3.30	3480	THR-274 (4.55), GLU-255 (4.74, 5.64)
	(±)-Catechin	−3.34	3280	GLU-255 (3.70), TRP-270 (5.54), LEU-267 (4.78, 5.28)
	Trigonelline	−3.90	1180	VAL-309 (3.67), SER-315 (4.51)
B-cell Lymphoma 2 (BCL2) [2W3L]	Palmitic amide	−3.07	6540	GLU-94 (3.55)
	Marmesin	−5.05	198.4	ARG-86 (4.76)
	α-D-Glucose	−2.59	23,400	GLU-119 (4.29, 5.21), ARG-123 (4.38), GLY-77 (6.00)
	α-Mangostin	−4.47	424.0	HIS-143 (5.44)
	Epicatechin	−4.23	617.0	GLU-124 (3.34), SER-126 (2.64, 3.07), ARG-26 (4.67, 5.24)
	(±)-Catechin	−3.50	2620	TRP-30 (5.05), ARG-57 (5.92), GLU-50 (4.09), GLU-13 (4.58)
	Trigonelline	−4.17	683.0	LYS-22 (4.55)
Catenin Beta 1 (CTNNB1) [1JPW]	Palmitic amide	−2.01	66,800	ARG-486 (5.20), ALA-522 (3.98)
	Marmesin	−4.38	514.0	-
	α-D-Glucose	−1.98	71,200	ASP-162 (4.99), GLN-165 (3.97, 4.45), LEU-160 (4.27)
	α-Mangostin	−3.44	2900	MET-662 (5.87)
	Epicatechin	−2.78	13,700	ARG-661 (6.07), PHE-660 (3.46), GLU-629 (4.28, 4.90)
	(±)-Catechin	−3.45	2860	ASN-380 (4.48), ASN-415 (4.09), ASP-413 (4.69, 4.77), GLN-379 (4.13)
	Trigonelline	−3.47	2780	LYS-281 (5.77)
Epidermal Growth Factor Receptor (EGFR) [1M17]	Palmitic amide	−2.67	17,200	ASP-776 (4.04), CYS-773 (3.25, 3.82)
	Marmesin	−5.23	140.6	MET-769 (4.72), THR-766 (3.31), CYS-751 (5.50)
	α-D-Glucose	−3.60	2040	THR-766 (3.22), GLU-738 (4.57), ASP-831 (3.42, 3.62), THR-830 (3.09)
	α-Mangostin	−4.46	456.0	PRO-770 (4.00)
	Epicatechin	−3.59	2080	ASP-783 (2.90, 3.84), GLN-958 (3.32)
	(±)-Catechin	−4.68	338.0	THR-766 (2.70), THR-830 (3.47), ASP-831 (3.60, 4.59), CYS-773 (3.80)
	Trigonelline	−3.49	2700	ARG-807 (5.30), ASN-676 (3.89), TYR-740 (6.23)
Hypoxia Inducible Factor 1 Alpha (HIF1A) [1PYX]	Palmitic amide	−3.97	1140	ARG-141 (5.10), PRO-136 (5.09), TYR-134 (5.21)
	Marmesin	−6.89	9.8	ASN-361 (2.31), LEU-359 (2.01), THR-356 (2.14, 2.79)
	α-D-Glucose	−3.78	1530	THR-152 (3.71), LEU-251 (3.78, 4.45)
	α-Mangostin	−4.97	228.0	VAL-135 (3.53), ASP-200 (4.35), LYS-85 (5.04)
	Epicatechin	−5.73	68.4	ASP-133 (1.99), VAL-135 (1.98), ASN-64 (1.90, 2.22)
	(±)-Catechin	−5.33	120.3	TRP-301 (5.42), SER-318 (3.33), GLN-280 (4.49)
	Trigonelline	−4.88	263.0	ARG-180 (6.19), VAL-214 (3.09)
Tumor Necrosis Factor (TNF) [2AZ5]	Palmitic amide	−4.06	820.0	LEU-93 (5.80), GLN-125 (4.46)
	Marmesin	−5.47	100.8	ASN-92 (3.75)
	α-D-Glucose	−2.84	10,800	GLU-116 (4.59, 5.29), SER-399 (2.53, 3.49), TYR-115 (6.15)
	α-Mangostin	−4.21	630.0	TYR-151 (5.91)
	Epicatechin	−3.13	5180	GLN-61 (4.37), LEU-120 (5.12), SER-60 (3.72, 4.24)
	(±)-Catechin	−4.03	860.0	SER-60 (4.19, 4.28), TYR-151 (4.82)
	Trigonelline	−3.65	1900	ASP-10 (3.41), LYS-11 (3.27)

Note: Kd values were estimated from binding free energies using Kd = e^(ΔG/RT)^ at T = 298 K, R = 0.001987 kcal/mol·K. Values below 100 µM are considered within the validity threshold for computational screening. All Kd values represent predicted estimates and require experimental confirmation.

**Table 5:** Comparison of GMPE with previously reported *G. mangostana* extracts in neurodegenerative research.

Extract Type	Major Compounds	Approach	Target Disease(s)	Key Limitation	Study
Ethanolic/methanolic peel extract	α-Mangostin, γ-mangostin	In vitro	AD, PD	Poor water solubility; single-compound focus	[10]
Isolated xanthones	α-Mangostin	In vitro/in silico	AD	Limited bioavailability; narrow mechanistic scope	[56,61]
Organic solvent pericarp extract	Xanthones, flavonoids	In vitro	PD	Lack of systems-level analysis	[62]
Database-predicted mangosteen compounds	Predicted phytochemicals	In silico	AD	No experimental metabolite confirmation	[63,64]
GMPE (present study)	Palmitic amide, quinic acid, marmesin, epicatechin	Metabolomics + systems pharmacology + docking	AD, PD, ALS	Computational predictions require biological validation	-

## Data Availability

All data generated or analyzed during this study are included in this published article.

## Supplementary Materials

The following supporting information can be downloaded at: https://www.mdpi.com/article/10.3390/life16040580/s1. Supplementary File S1: Table S1: UHPLC-QTOF-MS chromatographic and instrument parameters for GMPE phytochemical analysis; Table S2: Sequential inputs and outputs of the integrated GMPE systems pharmacology pipeline; Table S3: Putative therapeutic target genes of GMPE associated with AD-PD-ALS; Table S4: Hierarchy of 10 hub genes in the PPI network, based on topological measures of degree, betweenness, closeness, and MCC; Table S5: Top 20 enriched Kyoto Encyclopedia of Genes and Genomes (KEGG) pathways associated with GMPE-predicted targets and shared AD-PD-ALS genes; Supplementary File S2: TICs showed a complex array of bioactive compounds; Supplementary File S3: Compound selection framework for GMPE metabolites; Supplementary File S4: AD, PD, and ALS, targets from the GeneCards, DisGeNET, and OMIM databases.

## Abbreviations

The following abbreviations are used in this manuscript:

AChE	Acetylcholinesterase
AD	Alzheimer’s disease
ADMET	Absorption, distribution, metabolism, excretion, and toxicity
AKT1	Protein kinase B alpha
ALS	Amyotrophic lateral sclerosis
APP	Amyloid precursor protein
ATP	Adenosine triphosphate
BBB	Blood–brain barrier
BCL2	B-cell lymphoma 2
BOILED-Egg	Brain or intestinal estimated permeation method
BP	Biological process
CC	Cellular component
CNS	Central nervous system
CTNNB1	Catenin beta-1
DL	Drug-likeness
DMNC	Density of maximum neighborhood component
EGFR	Epidermal growth factor receptor
EPC	Edge percolated component
ESI	Electrospray ionization
FC	Fold change
GMPE	*Garcinia mangostana* peel extract
GO	Gene ontology
HIF1A	Hypoxia-inducible factor-1 alpha
IC_50_	Half-maximal inhibitory concentration
IL	Interleukin
KEGG	Kyoto Encyclopedia of Genes and Genomes
LC–MS	Liquid chromatography–mass spectrometry
MCC	Maximal clique centrality
MD	Molecular docking
MF	Molecular function
MNC	Maximum neighborhood component
MW	Molecular weight
OB	Oral bioavailability
PDB	Protein data bank
