# Supplementary material for: Mapping the Polar Neuro-Interactome of Garcinia mangostana Against the AD-PD-ALS Nexus
Source: Life (Basel). 2026 Apr 1;16(4):580. doi: 10.3390/life16040580 (PMC13117457; doi:10.3390/life16040580)
Supplement: Supplementary file 1 [file life-16-00580-s001.zip › Supplementary file S1_Table 1, 2, 3.pdf]

Table S1: UHPLC-QTOF-MS chromatographic and instrument parameters for GMPE phytochemical analysis.

Parameter	Condition
**Instrument**	Agilent 6200/6500 series UHPLC-QTOF-MS
**Column**	Zorbax Eclipse Plus C18
**Column temperature**	Ambient
**Injection volume**	2 µL
**Sample concentration**	10 mg/mL in water
**Flow rate**	0.2 mL/min
**Mobile phase A**	0.1% formic acid in water
**Mobile phase B**	Acetonitrile
**Gradient — 0 min**	100% A / 0% B
**Gradient — 35 min**	10% A / 90% B
**Gradient — 37 min**	100% A / 0% B (re-equilibration)
**Ionization source**	Agilent Jet Stream ESI
**Ionization modes**	Positive and Negative (Auto-MS/MS)
**m/z range**	100–1500
**Capillary voltage**	4000 V
**Gas temperature**	350°C
**Fragmentor voltage**	175 V
**Mass calibration**	Internal Reference Mass (IRM)
**Data processing software**	MassHunter Workstation B.08.00
**Library**	Metlin Metabolites PCDL

Table S2: Sequential inputs and outputs of the integrated GMPE systems pharmacology pipeline.

Step	Process	Input	Output	N
1	UHPLC-QTOF-MS profiling	GMPE aqueous extract	Identified metabolites	17
2	ADMET/BBB filtering	17 identified metabolites	Prioritized bioactives	7
3	Swiss Target Prediction	7 prioritized bioactives (SMILES)	Predicted protein targets	233
4	Disease gene retrieval	GeneCards + DisGeNET + OMIM	AD∪PD∪ALS gene set	976 (common core)
5	Target-disease intersection	Predicted targets ∩ Common core genes	Intersected therapeutic targets	121
6	PPI network construction	121 intersected targets (STRING ≥0.900)	Connected network	98 nodes/250 edges
7	Hub gene identification	PPI network (CytoHubba, 11 algorithms)	Hub genes ranked by degree	10
8	Functional enrichment	121 intersected targets (ShinyGO)	Enriched pathways (FDR<0.05)	20 KEGG pathways
9	Molecular docking	7 bioactives × 8 hub targets	Binding energies + Kd (µM)	56 poses

Table S3: Putative therapeutic target genes of GMPE associated with AD-PD-ALS.

Number	Gene symbol	Annotation
1	ABCB1	ATP-dependent translocase ABCB1
2	ABCC1	Multidrug resistance-associated protein 1
3	ABCG2	Broad substrate specificity ATP-binding cassette transporter ABCG2
4	ACHE	Acetylcholinesterase
5	ADA	Adenosine deaminase
6	ADORA2A	Adenosine receptor A2a
7	AGTR1	Type-1 angiotensin II receptor
8	AHCY	Adenosylhomocysteinase
9	ALDH2	Aldehyde dehydrogenase, mitochondrial
10	ALOX5	Arachidonate 5-lipoxygenase
11	APP	Gamma-secretase C-terminal fragment 50
12	AR	Androgen receptor

13	ASAH1	Acid ceramidase subunit alpha
14	AURKA	Aurora kinase A
15	BCL2	Apoptosis regulator Bcl-2
16	BRD4	Bromodomain-containing protein 4
17	CA1	Carbonic anhydrase 1
18	CA2	Carbonic anhydrase 2
19	CA9	Carbonic anhydrase 9
20	CDK1	Cyclin-dependent kinase 1
21	CDK5	Cyclin-dependent-like kinase 5
22	CHRNA4	Neuronal acetylcholine receptor subunit alpha-4
23	COMT	Catechol O-methyltransferase
24	CREBBP	CREB-binding protein
25	CSNK1D	Casein kinase I isoform delta
26	CSNK1E	Casein kinase I isoform epsilon
27	CSNK2A1	Casein kinase II subunit alpha
28	CTNNB1	Catenin beta-1
29	CXCR2	C-X-C chemokine receptor type 2
30	CYP19A1	Aromatase
31	CYP1B1	Cytochrome P450 1B1
32	DNMT1	DNA (cytosine-5)-methyltransferase 1
33	DYRK1A	Dual specificity tyrosine-phosphorylation-regulated kinase 1A
34	EGFR	Epidermal growth factor receptor
35	EIF2AK3	Eukaryotic translation initiation factor 2-alpha kinase 3
36	ERBB2	Receptor tyrosine-protein kinase erbB-2
37	ERBB4	Receptor tyrosine-protein kinase erbB-4
38	ESR1	Estrogen receptor
39	ESR2	Estrogen receptor beta
40	ESRRB	Steroid hormone receptor ERR2
41	FAAH	Fatty-acid amide hydrolase 1
42	FASN	3-hydroxyacyl-[acyl-carrier-protein] dehydratase
43	FGFR1	Fibroblast growth factor receptor 1
44	FKBP1A	Peptidyl-prolyl cis-trans isomerase FKBP1A
45	FLT1	Vascular endothelial growth factor receptor 1
46	GABRA1	Gamma-aminobutyric acid receptor subunit alpha-1
47	GABRB3	Gamma-aminobutyric acid receptor subunit beta-3
48	GABRG2	Gamma-aminobutyric acid receptor subunit gamma-2
49	GSK3B	Glycogen synthase kinase-3 beta
50	HDAC1	Histone deacetylase 1
51	HDAC2	Histone deacetylase 2
52	HDAC3	Histone deacetylase 3
53	HDAC6	Histone deacetylase 6
54	HIF1A	Hypoxia-inducible factor 1-alpha
55	HMGCR	3-hydroxy-3-methylglutaryl-coenzyme A reductase
56	HSP90AA1	Heat shock protein HSP 90-alpha

57	HSP90AB1	Heat shock protein HSP 90-beta
58	HSP90B1	Endoplasmin
59	HTR2A	5-hydroxytryptamine receptor 2A
60	IDH1	Isocitrate dehydrogenase 1
61	IGF1R	Insulin-like growth factor 1 receptor alpha chain
62	IMPDH2	Inosine-5'-monophosphate dehydrogenase 2
63	JAK1	Tyrosine-protein kinase JAK1
64	JAK2	Tyrosine-protein kinase JAK2
65	JAK3	Tyrosine-protein kinase JAK3
66	KCNH2	Potassium voltage-gated channel subfamily H member 2
67	KDR	Vascular endothelial growth factor receptor 2
68	KIF11	Kinesin-like protein KIF11
69	KIT	Mast/stem cell growth factor receptor Kit
70	LRRK2	Leucine-rich repeat serine/threonine-protein kinase 2
71	MAOA	Amine oxidase [flavin-containing] A
72	MAOB	Amine oxidase [flavin-containing] B
73	MAP2K1	Dual specificity mitogen-activated protein kinase kinase 1
74	MAP3K5	Mitogen-activated protein kinase kinase kinase 5
75	MAPK14	Mitogen-activated protein kinase 14
76	MAPKAPK2	MAP kinase-activated protein kinase 2
77	MAPT	Microtubule-associated protein tau
78	MCL1	Induced myeloid leukemia cell differentiation protein Mcl-1
79	MET	Hepatocyte growth factor receptor
80	MIF	Macrophage migration inhibitory factor
81	MMP1	22 kDa interstitial collagenase
82	MMP14	Matrix metalloproteinase-14
83	MMP2	72 kDa type IV collagenase
84	MMP7	Matrilysin
85	MMP9	67 kDa matrix metalloproteinase-9
86	MPO	Myeloperoxidase heavy chain
87	NCSTN	Nicastrin
88	NFKB1	Nuclear factor NF-kappa-B p105 subunit
89	NR3C1	Glucocorticoid receptor
90	PARP1	Poly [ADP-ribose] polymerase 1
91	PIK3CA	Phosphatidylinositol 4,5-bisphosphate 3-kinase catalytic subunit alpha isoform
92	PIM1	Serine/threonine-protein kinase pim-1
93	PLA2G7	Platelet-activating factor acetylhydrolase
94	PLAT	Tissue-type plasminogen activator chain A
95	PLAU	Urokinase-type plasminogen activator short chain A
96	PNP	Purine nucleoside phosphorylase
97	PPARG	Peroxisome proliferator-activated receptor gamma
98	PRKCE	Protein kinase C epsilon type
99	PRKCQ	Protein kinase C theta type

100	PSEN1	Presenilin-1 CTF subunit
101	PSEN2	Presenilin-2 CTF subunit
102	PTGS1	Prostaglandin G/H synthase 1
103	PTGS2	Prostaglandin G/H synthase 2
104	PTPN1	Tyrosine-protein phosphatase non-receptor type 1
105	RELA	Transcription factor p65
106	RPS6KB1	Ribosomal protein S6 kinase beta-1
107	SIRT1	NAD-dependent protein deacetylase sirtuin-1
108	SLC6A2	Sodium-dependent noradrenaline transporter
109	SRC	Proto-oncogene tyrosine-protein kinase Src
110	STAT1	Signal transducer and activator of transcription 1-alpha/beta
111	TBXAS1	Thromboxane A synthase 1
112	TEK	Angiopoietin-1 receptor
113	TERT	Telomerase reverse transcriptase
114	TLR7	Toll-like receptor 7
115	TNF	Tumor necrosis factor, membrane form
116	TNNI3	Troponin I, cardiac muscle
117	TNNT2	Troponin T, cardiac muscle
118	TRPV1	Transient receptor potential cation channel subfamily V member 1
119	TYK2	Non-receptor tyrosine-protein kinase TYK2
120	TYR	Tyrosinase
121	XDH	Xanthine dehydrogenase/oxidase

Table S4: Hierarchy of 10 hub genes in the PPI network, based on topological measures of degree, betweenness, closeness, and MCC.

Hub Gene Name	MCC	DMNC	MNC	Degree	EPC	BottleNeck	EcCentricity	Closeness	Radiality	Betweenness	Stress	Clustering Coefficient
CTNNB1	1203	0.37524	19	22	26.726	54	0.15136	49.35	8.08059	1898.479	6690	0.24675
SRC	1323	0.45334	17	21	26.21	19	0.12974	48.04286	7.96707	1693.799	5860	0.27619
ESR1	1370	0.35044	21	21	26.81	10	0.12974	48.12619	7.96707	1245.447	4564	0.29524
HSP90AA1	203	0.24286	17	18	23.851	12	0.15136	46.3	7.95675	1219.663	4828	0.19608
EGFR	1560	0.47763	17	17	26.044	5	0.12974	44.87619	7.84323	343.90681	2252	0.43382
HDAC1	378	0.32307	16	16	23.47	5	0.12974	43.50952	7.77099	560.84919	2126	0.3
PIK3CA	1293	0.5237	13	14	24.398	3	0.12974	41.95952	7.71939	281.91555	1160	0.45055
HSP90AB1	196	0.30405	14	14	22.949	6	0.15136	43.8	7.88451	521.04321	2796	0.2967
CREBBP	370	0.40874	13	13	23.941	1	0.12974	42.45952	7.77099	164.85246	996	0.41026
JAK2	529	0.54149	12	13	23.561	2	0.11352	38.32024	7.37883	234.17019	1728	0.47436

Table S5: Top 20 enriched Kyoto Encyclopedia of Genes and Genomes (KEGG) pathways associated with GMPE-predicted targets and shared AD-PD-ALS genes.

ID	Pathway	FDR	nGenes	Gene names
hsa05200	Pathways in cancer	1.02×10^{-27}	36	CREBBP, CTNNB1, AGTR1, EGFR, ERBB2, ESR1, ESR2, FGFR1, GSK3B, HDAC1, HDAC2, HIF1A, HSP90AA1, HSP90AB1, IGF1R, AR, JAK1, JAK2, JAK3, KIT, MET, MMP1, MMP2, MMP9, NFKB1, PIK3CA, PIM1, PPARG, MAP2K1, PTGS2, BCL2, RELA, RPS6KB1, STAT1, TERT, HSP90B1
hsa01100	Metabolic pathways	1.10×10^{-07}	27	ADA, COMT, CYP19A1, DNMT1, AHCY, ALDH2, FASN, SIRT1, ALOX5, HMGCR, IDH1, IMPDH2, MAOA, MAOB, ASAH1, MIF, PNP, PIK3CA, PTGS1, PTGS2, TBXAS1, TYR, XDH, CA1, CA2, CA9, PLA2G7
hsa04151	PI3K-Akt signaling pathway	7.06×10^{-18}	24	EGFR, ERBB2, ERBB4, FGFR1, FLT1, GSK3B, HSP90AA1, HSP90AB1, IGF1R, JAK1, JAK2, JAK3, KDR, KIT, MCL1, MET, NFKB1, PIK3CA, MAP2K1, BCL2, RELA, RPS6KB1, TEK, HSP90B1
hsa05206	MicroRNAs in cancer	7.26×10^{-16}	21	CREBBP, CYP1B1, DNMT1, EGFR, ERBB2, SIRT1, HDAC1, HDAC2, MCL1, MET, MMP9, ABCC, NFKB1, ABCB1, PIK3CA, PIM1, PLAU, PRKCE, MAP2K1, PTGS2, BCL2
hsa05215	Prostate cancer	7.19×10^{-23}	19	CREBBP, CTNNB1, EGFR, ERBB2, FGFR1, GSK3B, HSP90AA1, HSP90AB1, IGF1R, AR, MMP9, NFKB1, PIK3CA, PLAT, PLAU, MAP2K1, BCL2, RELA, HSP90B1
hsa05205	Proteoglycans in cancer	7.88×10^{-17}	19	MAPK14, CTNNB1, EGFR, ERBB2, ERBB4, ESR1, FGFR1 HIF1A, IGF1R, KDR, MET, MMP2, MMP9, PIK3CA, PLAU, MAP2K1, RPS6KB1, SRC, TNF
hsa05022	Pathways of neurodegeneration-multiple diseases	7.88×10^{-11}	19	CDK5, TPTEP2-CSNK1E, LRRK2, MAPK14, CSNK1E, CSNK2A1, CTNNB1, GSK3B, APP, MAPT, MAP3K5, NFKB1, MAP2K1, PSEN1, PTGS2, BCL2, RELA, TNF, EIF2AK3
hsa04010	MAPK signaling pathway	4.61×10^{-13}	18	MAPK14, EGFR, ERBB2, ERBB4, FGFR1, FLT1, IGF1R, KDR, KIT, MAPT, MAP3K5, MET, NFKB1, MAP2K1, RELA, TEK, TNF, MAPKAPK2
hsa05165	Human papillomavirus infection	2.30×10^{-12}	18	CREBBP, CTNNB1, EGFR, GSK3B, HDAC1, HDAC2, JAK1, NFKB1, PIK3CA, MAP2K1, PSEN1, PTGS2, RELA, RPS6KB1, STAT1, TERT, TNF, TYK2
hsa05010	Alzheimer disease	2.50×10^{-11}	18	CDK5, TPTEP2-CSNK1E, CSNK1E, CSNK2A1, CTNNB1, NCSTN, GSK3B, APP, MAPT, MAP3K5, NFKB1, PIK3CA, MAP2K1, PSEN1, PTGS2, RELA, TNF, EIF2AK3
hsa05207	Chemical carcinogenesis-receptor activation	3.99×10^{-14}	17	CHRNA4, CYP1B1, EGFR, ESR1, ESR2, HSP90AA1, HSP90AB1, AR, JAK2, NFKB1, PIK3CA, MAP2K1, BCL2, RELA, RPS6KB1, SRC, HSP90B1

hsa05417	Lipid and atherosclerosis	3.99×10^{-14}	17	MAPK14, GSK3B, HSP90AA1, HSP90AB1, JAK2, MAP3K5, MMP1, MMP9, NFKB1, PIK3CA, PPARG, BCL2, RELA, SRC, TNF, HSP90B1, EIF2AK3
hsa05418	Fluid shear stress and atherosclerosis	1.46×10^{-15}	16	MAPK14, CTNNB1, HSP90AA1, HSP90AB1, KDR, MAP3K5, MMP2, MMP9, NFKB1, PIK3CA, PLAT, BCL2, RELA, SRC, TNF, HSP90B1
hsa05167	Kaposi sarcoma-associated herpesvirus infection	1.22×10^{-13}	16	CREBBP, MAPK14, CTNNB1, GSK3B, HIF1A, JAK1, JAK2, NFKB1, PIK3CA, MAP2K1, PTGS2, RELA, SRC, STAT1, TYK2, MAPKAPK2
hsa05161	Hepatitis B	1.64×10^{-13}	15	CREBBP, MAPK14, JAK1, JAK2, JAK3, MMP9, NFKB1, PIK3CA, MAP2K1, BCL2, RELA, SRC, STAT1, TNF, TYK2
hsa04015	Rap1 signaling pathway	6.85×10^{-11}	14	ADORA2A, MAPK14, CTNNB1, EGFR, FGFR1, FLT1, IGF1R, KDR, KIT, MET, PIK3CA, MAP2K1, SRC, TEK
hsa05163	Human cytomegalovirus infection	1.31×10^{-10}	14	MAPK14, CTNNB1, EGFR, GSK3B, CXCR2, JAK1, NFKB1, PIK3CA, MAP2K1, PTGS2, RELA, RPS6KB1, SRC, TNF
hsa01521	EGFR tyrosine kinase inhibitor resistance	8.75×10^{-15}	13	EGFR, ERBB2, GSK3B, IGF1R, JAK1, JAK2, KDR, MET, PIK3CA, MAP2K1, BCL2, RPS6KB1, SRC
hsa05235	PD-L1 expression and PD-1 checkpoint pathway in cancer	3.99×10^{-14}	13	MAPK14, CSNK2A1, EGFR, HIF1A, JAK1, JAK2, NFKB1, PIK3CA, PRKCQ, MAP2K1, RELA, RPS6KB1, STAT1
hsa01522	Endocrine resistance	7.91×10^{-14}	13	MAPK14, EGFR, ERBB2, ESR1, ESR2, IGF1R, MMP2, MMP9, PIK3CA, MAP2K1, BCL2, RPS6KB1, SRC
