# Supplementary material for: Mapping the Polar Neuro-Interactome of Garcinia mangostana Against the AD-PD-ALS Nexus
Source: Life (Basel). 2026 Apr 1;16(4):580. doi: 10.3390/life16040580 (PMC13117457; doi:10.3390/life16040580)
Supplement: Supplementary file 1 [file life-16-00580-s001.zip › Supplementary file S2_TICs showed a complex array of bioactive compounds.pdf]

Compound Identification (Positive (ESI$^+$) modes)

			General													Database Search				Library Search	
Cpd	Name	Formula	Label	Obs m/z	Mass	Start	RT	End	Height	Score	Base Peak	ID Source	Polarity	ining Algorith	File	Score (DB)	Mass (DB)	Diff (DB, ppm)	Diff (DB, mDa)	Score (Lib)	Lib/DB
22	Choline	C5 H14 N O	Cpd 22: Choline; C5 H14 N O; 1.898	104.1073	104.1078	1.889	1.898	1.906	2596871	90.91	104.1072	DBSearch-Lib	Positive	Find by Auto I	1_050_65_P1001.d	86.74	104.1075	-2.67	-0.28	95.08	D:\MassHunter\PCDL\Metlin_Metabolites_AM_PCDL.cdb
23	6-(alpha-D-Glucosaminyl)-1D-myo-inositol	C12 H23 N O10	Cpd 23: 6-(alpha-D-Glucosaminyl)-1D-myo-inositol; C12 H23 N O10; 1.947	342.1394	341.1321	1.939	1.947	1.956	131803	49.81	342.1386	DBSearch	Positive	Find by Auto I	1_050_65_P1001.d	99.61	341.1322	0.28	0.1	0	
24	Trimethylammonioacetate	C5 H12 N O2	Cpd 24: Trimethylammonioacetate; C5 H12 N O2; 2.023	118.0863	118.0868	2.015	2.023	2.031	782043	43.9	121.0508	DBSearch	Positive	Find by Auto I	1_050_65_P1001.d	87.79	118.0868	0.05	0.01	0	
25	α-D-Glucose	C6 H12 O6	Cpd 25: α-D-Glucose; C6 H12 O6; 2.023	203.0525	180.0635	1.914	2.023	2.132	770185	88.72	203.0522	DBSearch-Lib	Positive	Find by Auto I	1_050_65_P1001.d	97.22	180.0634	-0.56	-0.1	80.21	D:\MassHunter\
28	Maltose	C12 H22 O11	Cpd 28: Maltose; C12 H22 O11; 2.199	365.1058	342.1166	2.09	2.199	2.308	1472537	95.97	365.1052	DBSearch-Lib	Positive	Find by Auto I	1_050_65_P1001.d	98.23	342.1162	-1.03	-0.35	93.72	D:\MassHunter\
29	Trigonelline	C7 H8 N O2	Cpd 29: Trigonelline; C7 H8 N O2; 2.199	138.0552	138.0558	2.191	2.199	2.207	1856962	89.1	138.0549	DBSearch-Lib	Positive	Find by Auto I	1_050_65_P1001.d	87.29	138.0555	-2.07	-0.29	90.91	D:\MassHunter\
30	Valiolone	C7 H12 O6	Cpd 30: Valiolone; C7 H12 O6; 2.299	215.0527	192.0634	2.24	2.299	2.357	230039	49.78	215.0523	DBSearch	Positive	Find by Auto I	1_050_65_P1001.d	99.56	192.0634	-0.29	-0.06	0	
31	Firocoxib	C17 H20 O5 S	Cpd 31: Firocoxib; C17 H20 O5 S; 2.324	337.1103	336.1031	2.316	2.324	2.332	158477	43.49	337.1098	DBSearch	Positive	Find by Auto I	1_050_65_P1001.d	86.97	336.1031	0.11	0.04	0	
32	Malonylcarnitine	C10 H18 N O6	Cpd 32: Malonylcarnitine; C10 H18 N O6; 2.449	248.113	248.1136	2.441	2.449	2.458	110001	49.76	248.1122	DBSearch	Positive	Find by Auto I	1_050_65_P1001.d	99.52	248.1134	-0.76	-0.19	0	
33	Sucrose	C12 H22 O11	Cpd 33: Sucrose; C12 H22 O11; 2.500	365.1056	342.1163	2.391	2.5	2.609	1029469	99.26	365.1051	DBSearch-Lib	Positive	Find by Auto I	1_050_65_P1001.d	99.36	342.1162	-0.16	-0.05	99.16	D:\MassHunter\
38	2-(beta-D-Glucosyl)-sn-glycerol	C9 H18 O8	Cpd 38: 2-(beta-D-Glucosyl)-sn-glycerol; C9 H18 O8; 2.751	277.0894	254.1002	2.742	2.751	2.759	132815	49.83	277.0893	DBSearch	Positive	Find by Auto I	1_050_65_P1001.d	99.65	254.1002	-0.03	-0.01	0	
40	(-)-Dioxibrassinin	C11 H12 N2 O2 S2	Cpd 40: (-)-Dioxibrassinin; C11 H12 N2 O2 S2; 2.801	306.9967	268.0338	2.793	2.801	2.81	329837	35.97	172.9752	DBSearch	Positive	Find by Auto I	1_050_65_P1001.d	71.94	268.034	0.87	0.23	0	
45	Galactose-beta-1,4-xylose	C11 H20 O10	Cpd 45: Galactose-beta-1,4-xylose; C11 H20 O10; 3.102	335.0946	312.1059	2.994	3.102	3.211	286378	39.84	335.0942	DBSearch	Positive	Find by Auto I	1_050_65_P1001.d	79.68	312.1056	-0.97	-0.3	0	
51	Adenine	C5 H5 N5	Cpd 51: Adenine; C5 H5 N5; 3.504	136.0615	135.0542	3.496	3.504	3.512	166349	43.35	136.0616	DBSearch	Positive	Find by Auto I	1_050_65_P1001.d	86.7	135.0545	2.12	0.29	0	
52	4-Guanidinobutanoic acid	C5 H11 N3 O2	Cpd 52: 4-Guanidinobutanoic acid; C5 H11 N3 O2; 3.529	146.0923	145.085	3.521	3.529	3.537	174405	88.64	149.0229	DBSearch-Lib	Positive	Find by Auto I	1_050_65_P1001.d	87.77	145.0851	0.77	0.11	89.5	D:\MassHunter\
63	D-Galactopyranosyl-(1->3)-D-galactopyranosyl-(1->3)-L-arabinose	C17 H30 O15	Cpd 63: D-Galactopyranosyl-(1->3)-D-galactopyranosyl-(1->3)-L-arabinose; C1	497.1468	474.1576	4.5	4.508	4.516	71698	48.69	497.1457	DBSearch	Positive	Find by Auto I	1_050_65_P1001.d	97.37	474.1585	1.89	0.9	0	
65	DL-o-Tyrosine	C9 H11 N O3	Cpd 65: DL-o-Tyrosine; C9 H11 N O3; 4.633	182.0808	181.0736	4.625	4.633	4.641	149677	49.48	164.9854	DBSearch	Positive	Find by Auto I	1_050_65_P1001.d	98.95	181.0739	1.52	0.28	0	
66	Cystine	C6 H12 N2 O4 S2	Cpd 66: Cystine; C6 H12 N2 O4 S2; 4.658	241.0317	240.0247	4.65	4.658	4.666	118871	39.52	241.0316	DBSearch	Positive	Find by Auto I	1_050_65_P1001.d	79.05	240.0238	-3.64	-0.87	0	
67	Ethyl beta-D-glucopyranoside	C8 H16 O6	Cpd 67: Ethyl beta-D-glucopyranoside; C8 H16 O6; 4.659	231.0839	208.0946	4.6	4.658	4.717	381882	49.89	231.0838	DBSearch	Positive	Find by Auto I	1_050_65_P1001.d	99.78	208.0947	0.32	0.07	0	
71	(R)-N-Methylsalsolinol	C11 H15 N O2	Cpd 71: (R)-N-Methylsalsolinol; C11 H15 N O2; 4.934	194.1175	193.1102	4.85	4.934	5.018	215174	49.97	194.1174	DBSearch	Positive	Find by Auto I	1_050_65_P1001.d	99.94	193.1103	0.43	0.08	0	
74	2'-Aminoacetophenone	C8 H9 N O	Cpd 74: 2'-Aminoacetophenone; C8 H9 N O; 4.959	136.0754	135.0681	4.951	4.959	4.967	158439	43.28	120.9525	DBSearch	Positive	Find by Auto I	1_050_65_P1001.d	86.57	135.0684	2.09	0.28	0	
76	Kojic Acid	C6 H6 O4	Cpd 76: Kojic Acid; C6 H6 O4; 5.135	143.0338	142.0265	5.127	5.135	5.143	66728	97.41	143.0332	DBSearch-Lib	Positive	Find by Auto I	1_050_65_P1001.d	99.6	142.0266	0.93	0.13	95.22	D:\MassHunter\
78	2,3-Butanediol glucoside	C10 H20 O7	Cpd 78: 2,3-Butanediol glucoside; C10 H20 O7; 5.211	275.1102	252.1209	5.202	5.211	5.219	507963	49.79	275.1096	DBSearch	Positive	Find by Auto I	1_050_65_P1001.d	99.59	252.1209	0.05	0.01	0	
80	Methyl-2-alpha-L-fucopyranosyl-beta-D-galactoside	C13 H24 O10	Cpd 80: Methyl-2-alpha-L-fucopyranosyl-beta-D-galactoside; C13 H24 O10; 5.3	363.1256	340.1364	5.352	5.361	5.369	69222	49.37	363.1258	DBSearch	Positive	Find by Auto I	1_050_65_P1001.d	98.75	340.1369	1.64	0.56	0	
85	Isopropyl beta-D-glucoside	C9 H18 O6	Cpd 85: Isopropyl beta-D-glucoside; C9 H18 O6; 5.713	245.0994	222.1102	5.704	5.713	5.721	133058	49.94	245.0993	DBSearch	Positive	Find by Auto I	1_050_65_P1001.d	99.88	222.1103	0.55	0.12	0	
88	Isopropyl apiosylglucoside	C14 H26 O10	Cpd 88: Isopropyl apiosylglucoside; C14 H26 O10; 5.988	377.1414	354.1521	5.905	5.988	6.072	1428680	49.55	377.1417	DBSearch	Positive	Find by Auto I	1_050_65_P1001.d	99.1	354.1526	1.28	0.45	0	
94	Leonuriside A	C14 H20 O9	Cpd 94: Leonuriside A; C14 H20 O9; 6.340	355.0997	332.1104	6.332	6.34	6.348	63135	49.5	355.0991	DBSearch	Positive	Find by Auto I	1_050_65_P1001.d	99.01	332.1107	0.96	0.32	0	
118	Cynaroside A	C21 H32 O10	Cpd 118: Cynaroside A; C21 H32 O10; 8.473	467.188	444.1987	8.465	8.473	8.481	44911	48.82	467.1872	DBSearch	Positive	Find by Auto I	1_050_65_P1001.d	97.64	444.1995	1.91	0.85	0	
127	3-Hydroxycoumarin	C9 H6 O3	Cpd 127: 3-Hydroxycoumarin; C9 H6 O3; 9.327	163.039	162.0318	9.318	9.327	9.335	114794	43.81	163.0385	DBSearch	Positive	Find by Auto I	1_050_65_P1001.d	87.61	162.0317	-0.4	-0.06	0	
129	2-[4-(3-Hydroxypropyl)-2-methoxyphenoxy]-1,3-propanediol 1-glucosi	C19 H30 O10	Cpd 129: 2-[4-(3-Hydroxypropyl)-2-methoxyphenoxy]-1,3-propanediol 1-gluco	441.1727	418.1834	9.343	9.351	9.36	54371	49.49	441.1716	DBSearch	Positive	Find by Auto I	1_050_65_P1001.d	98.98	418.1839	1.21	0.51	0	
139	Procyanidin B2	C30 H26 O12	Cpd 139: Procyanidin B2; C30 H26 O12; 10.029	579.1502	578.1426	10.021	10.029	10.038	1096683	93.87	579.1486	DBSearch-Lib	Positive	Find by Auto I	1_050_65_P1001.d	97.49	578.1424	-0.33	-0.19	90.24	D:\MassHunter\
140	Procyanidin B3	C30 H26 O12	Cpd 140: Procyanidin B3; C30 H26 O12; 10.054	601.1311	578.1417	10.046	10.054	10.062	282208	49.36	601.1302	DBSearch	Positive	Find by Auto I	1_050_65_P1001.d	98.71	578.1424	1.19	0.69	0	
142	Phenylethyl primeveroside	C19 H28 O10	Cpd 142: Phenylethyl primeveroside; C19 H28 O10; 10.230	439.1569	416.1676	10.222	10.23	10.238	169674	48.96	439.157	DBSearch	Positive	Find by Auto I	1_050_65_P1001.d	97.92	416.1682	1.49	0.62	0	
150	Epicatechin	C15 H14 O6	Cpd 150: Epicatechin; C15 H14 O6; 10.657	291.0866	290.0792	10.648	10.657	10.665	758402	97.94	139.0388	DBSearch-Lib	Positive	Find by Auto I	1_050_65_P1001.d	99.3	290.079	-0.71	-0.21	96.58	D:\MassHunter\
151	cis-3,4-Leucopelargonidin	C15 H14 O6	Cpd 151: cis-3,4-Leucopelargonidin; C15 H14 O6; 10.681	313.0682	290.0789	10.673	10.681	10.69	107904	49.62	313.0674	DBSearch	Positive	Find by Auto I	1_050_65_P1001.d	99.24	290.079	0.63	0.18	0	
152	2-Methoxy-1,4-benzoquinone	C7 H6 O3	Cpd 152: 2-Methoxy-1,4-benzoquinone; C7 H6 O3; 10.682	139.039	138.0317	10.623	10.682	10.74	1050044	43.86	139.0387	DBSearch	Positive	Find by Auto I	1_050_65_P1001.d	87.71	138.0317	0.04	0	0	
159	Epifisetinidol-(4beta->8)-catechin	C30 H26 O11	Cpd 159: Epifisetinidol-(4beta->8)-catechin; C30 H26 O11; 11.234	563.1539	562.1467	11.226	11.234	11.242	113586	49.23	563.153	DBSearch	Positive	Find by Auto I	1_050_65_P1001.d	98.45	562.1475	1.51	0.85	0	
180	Unshuoside A	C16 H28 O7	Cpd 180: Unshuoside A; C16 H28 O7; 13.041	355.1724	332.1832	13.033	13.041	13.049	65327	49.26	355.1725	DBSearch	Positive	Find by Auto I	1_050_65_P1001.d	98.53	332.1835	0.94	0.31	0	
183	Isolariciresinol 9-O-beta-D-glucoside	C26 H34 O11	Cpd 183: Isolariciresinol 9-O-beta-D-glucoside; C26 H34 O11; 13.242	545.1987	522.2092	13.234	13.242	13.25	96439	46.94	545.198	DBSearch	Positive	Find by Auto I	1_050_65_P1001.d	93.89	522.2101	1.66	0.87	0	
190	2,6 dimethylheptanoyl carnitine	C16 H32 N O4	Cpd 190: 2,6 dimethylheptanoyl carnitine; C16 H32 N O4; 13.668	302.2326	302.2331	13.66	13.668	13.677	126803	49.91	302.2327	DBSearch	Positive	Find by Auto I	1_050_65_P1001.d	99.82	302.2331	0	0	0	
193	S-Furanopetasitin	C24 H32 O5 S	Cpd 193: S-Furanopetasitin; C24 H32 O5 S; 13.945	433.204	432.1968	13.936	13.945	13.953	77334	42.78	433.2041	DBSearch	Positive	Find by Auto I	1_050_65_P1001.d	85.56	432.197	0.61	0.26	0	
220	Hulupinic acid	C15 H20 O4	Cpd 220: Hulupinic acid; C15 H20 O4; 16.379	287.1254	264.1363	16.37	16.379	16.387	48738	49.44	288.2893	DBSearch	Positive	Find by Auto I	1_050_65_P1001.d	98.88	264.1362	-0.35	-0.09	0	
261	Palmitic amide	C16 H33 N O	Cpd 261: Palmitic amide; C16 H33 N O; 20.696	256.2633	255.2561	20.687	20.696	20.704	146262	49.88	256.2637	DBSearch	Positive	Find by Auto I	1_050_65_P1001.d	99.77	255.2562	0.61	0.15	0	
263	C16 Sphinganine	C16 H35 N O2	Cpd 263: C16 Sphinganine; C16 H35 N O2; 20.721	274.2754	273.268	20.662	20.721	20.779	14931002	46.77	274.2749	DBSearch	Positive	Find by Auto I	1_050_65_P1001.d	93.53	273.2668	-4.31	-1.18	0	
294	Alpha-CEHC	C16 H22 O4	Cpd 294: Alpha-CEHC; C16 H22 O4; 32.742	301.141	278.1518	32.734	32.742	32.751	53220	49.91	301.1406	DBSearch	Positive	Find by Auto I	1_050_65_P1001.d	99.82	278.1518	0.02	0.01	0	
297	α-Mangostin	C24 H26 O6	Cpd 297: α-Mangostin; C24 H26 O6; 35.127	411.1804	410.1731	35.119	35.127	35.135	101452	93.5	355.1176	DBSearch-Lib	Positive	Find by Auto I	1_050_65_P1001.d	99.41	410.1729	-0.32	-0.13	87.58	D:\MassHunter\
298	Isoartocarpesin	C20 H18 O6	Cpd 298: Isoartocarpesin; C20 H18 O6; 35.177	355.1176	354.1103	35.168	35.177	35.185	22350	49.52	355.2833	DBSearch	Positive	Find by Auto I	1_050_65_P1001.d	99.05	354.1103	0.15	0.05	0	

Compound Identification (Negative (ESI$^-$) modes)

			General													Database Search				Library Search	
Cpd	Name	Formula	Label	obs m/z	Mass	Start	RT	End	Height	Score	Base Peak	ID Source	Polarity	ining Algorith	File	Score (DB)	Mass (DB)	Diff (DB, ppm)	Diff (DB, mDa)	Score (Lib)	Lib/DB
4	Galactaric acid	C6 H10 O8	Cpd 4: Galactaric acid; C6 H10 O8; 1.916	209.0303	210.0376	1.907	1.916	1.924	139631	97.29	85.0295	DBSearch-Lib	Negative	Find by Auto I	1_050_65_N1001.d	99.87	210.0376	-0.06	-0.01	94.71	D:\MassHunter\
6	D-Glucose 6-sulfate	C6 H12 O9 S	Cpd 6: D-Glucose 6-sulfate; C6 H12 O9 S; 1.953	259.0129	260.0201	1.883	1.953	2.024	666836	37.68	259.0126	DBSearch	Negative	Find by Auto I	1_050_65_N1001.d	75.36	260.0202	0.33	0.09	0	
7	L-Altruronic acid	C6 H10 O7	Cpd 7: L-Altruronic acid; C6 H10 O7; 2.041	193.0353	194.0426	2.033	2.041	2.049	369919	43.4	195.0506	DBSearch	Negative	Find by Auto I	1_050_65_N1001.d	86.8	194.0427	0.31	0.06	0	
8	Galactonic acid	C6 H12 O7	Cpd 8: Galactonic acid; C6 H12 O7; 2.066	195.0513	196.0585	1.983	2.066	2.15	1599049	95.42	195.051	DBSearch-Lib	Negative	Find by Auto I	1_050_65_N1001.d	99.46	196.0583	-1.2	-0.24	91.39	D:\MassHunter\
9	threo-Isocitric acid	C6 H8 O7	Cpd 9: threo-Isocitric acid; C6 H8 O7; 2.092	191.0199	192.0271	2.083	2.092	2.1	205755	43.43	85.0295	DBSearch	Negative	Find by Auto I	1_050_65_N1001.d	86.86	192.027	-0.72	-0.14	0	
13	Quinic acid	C7 H12 O6	Cpd 13: Quinic acid; C7 H12 O6; 2.305	191.0563	192.0636	2.184	2.305	2.426	6946591	95.16	191.0562	DBSearch-Lib	Negative	Find by Auto I	1_050_65_N1001.d	99.64	192.0634	-1.01	-0.19	90.67	D:\MassHunter\
15	Oxalosuccinic acid	C6 H6 O7	Cpd 15: Oxalosuccinic acid; C6 H6 O7; 2.468	189.0042	190.0115	2.434	2.468	2.501	250211	43.42	127.0037	DBSearch	Negative	Find by Auto I	1_050_65_N1001.d	86.84	190.0114	-0.56	-0.11	0	
17	Homoisocitrate	C7 H10 O7	Cpd 17: Homoisocitrate; C7 H10 O7; 2.618	205.0353	206.0426	2.535	2.618	2.702	394450	49.98	205.035	DBSearch	Negative	Find by Auto I	1_050_65_N1001.d	99.96	206.0427	0.3	0.06	0	
18	Sucrose	C12 H22 O11	Cpd 18: Sucrose; C12 H22 O11; 2.618	341.1089	342.1161	2.61	2.618	2.627	147604	98.55	341.1082	DBSearch-Lib	Negative	Find by Auto I	1_050_65_N1001.d	99.39	342.1162	0.4	0.14	97.71	D:\MassHunter\
19	Coriose	C7 H14 O7	Cpd 19: Coriose; C7 H14 O7; 2.643	209.0666	210.0738	2.635	2.643	2.652	50445	49.7	209.0663	DBSearch	Negative	Find by Auto I	1_050_65_N1001.d	99.4	210.074	0.49	0.1	0	
20	Maleic acid	C4 H4 O4	Cpd 20: Maleic acid; C4 H4 O4; 2.819	115.0037	116.011	2.811	2.819	2.827	21915	98.92	71.0133	DBSearch-Lib	Negative	Find by Auto I	1_050_65_N1001.d	99.77	116.011	-0.15	-0.02	98.07	D:\MassHunter\
22	Malic acid	C4 H6 O5	Cpd 22: Malic acid; C4 H6 O5; 2.869	133.0143	134.0216	2.786	2.869	2.953	172779	99.32	115.0036	DBSearch-Lib	Negative	Find by Auto I	1_050_65_N1001.d	99.92	134.0215	-0.24	-0.03	98.72	D:\MassHunter\
23	Arabinopyranobiose	C10 H18 O9	Cpd 23: Arabinopyranobiose; C10 H18 O9; 2.995	281.0876	282.0949	2.987	2.995	3.003	192412	49.67	281.0873	DBSearch	Negative	Find by Auto I	1_050_65_N1001.d	99.33	282.0951	0.72	0.2	0	
24	Shikimic acid	C7 H10 O5	Cpd 24: Shikimic acid; C7 H10 O5; 3.020	173.0455	174.0528	3.012	3.02	3.028	101653	95.34	130.9656	DBSearch-Lib	Negative	Find by Auto I	1_050_65_N1001.d	99.44	174.0528	0.37	0.06	91.25	D:\MassHunter\
27	D-Galactopyranosyl-(1->3)-D-galactopyranosyl-(1->3)-L-arabinose	C17 H30 O15	Cpd 27: D-Galactopyranosyl-(1->3)-D-galactopyranosyl-(1->3)-L-arabinose; C1	473.1506	474.1582	3.112	3.12	3.129	50781	48.16	473.1503	DBSearch	Negative	Find by Auto I	1_050_65_N1001.d	96.31	474.1585	0.64	0.31	0	
34	3-Furoic acid	C5 H4 O3	Cpd 34: 3-Furoic acid; C5 H4 O3; 3.597	111.0088	112.0161	3.589	3.597	3.606	77562	49.95	68.9962	DBSearch	Negative	Find by Auto I	1_050_65_N1001.d	99.89	112.016	-0.18	-0.02	0	
37	Citric acid	C6 H8 O7	Cpd 37: Citric acid; C6 H8 O7; 4.626	191.02	192.0272	4.618	4.626	4.634	167004	96.58	111.0088	DBSearch-Lib	Negative	Find by Auto I	1_050_65_N1001.d	99.46	192.027	-1.03	-0.2	93.71	D:\MassHunter\
41	Ascladiol	C7 H8 O4	Cpd 41: Ascladiol; C7 H8 O4; 4.727	155.035	156.0423	4.718	4.727	4.735	125530	49.94	136.9363	DBSearch	Negative	Find by Auto I	1_050_65_N1001.d	99.89	156.0423	-0.08	-0.01	0	
45	2,5-Dimethyl-3(2H)-furanone	C6 H8 O2	Cpd 45: 2,5-Dimethyl-3(2H)-furanone; C6 H8 O2; 5.003	111.0452	112.0524	4.995	5.003	5.011	127986	43.85	111.0451	DBSearch	Negative	Find by Auto I	1_050_65_N1001.d	87.71	112.0524	0.11	0.01	0	
46	Phthalate 3,4-cis-dihydrodiol	C8 H8 O6	Cpd 46: Phthalate 3,4-cis-dihydrodiol; C8 H8 O6; 5.053	199.0246	200.0318	5.044	5.053	5.061	29453	49.11	130.0867	DBSearch	Negative	Find by Auto I	1_050_65_N1001.d	98.23	200.0321	1.37	0.27	0	
50	Methyl-2-alpha-L-fucopyranosyl-beta-D-galactoside	C13 H24 O10	Cpd 50: Methyl-2-alpha-L-fucopyranosyl-beta-D-galactoside; C13 H24 O10; 5.4	339.1295	340.1368	5.421	5.429	5.438	35614	49.32	339.1275	DBSearch	Negative	Find by Auto I	1_050_65_N1001.d	98.65	340.1369	0.53	0.18	0	
54	Isopropyl apiosylglucoside	C14 H26 O10	Cpd 54: Isopropyl apiosylglucoside; C14 H26 O10; 6.032	353.145	354.1522	6.023	6.032	6.04	31082	49.33	207.0872	DBSearch	Negative	Find by Auto I	1_050_65_N1001.d	98.65	354.1526	1.2	0.42	0	
70	3-Glucosyl-2,3',4,4',6-pentahydroxybenzophenone	C19 H20 O11	Cpd 70: 3-Glucosyl-2,3',4,4',6-pentahydroxybenzophenone; C19 H20 O11; 7.91	423.0936	424.1008	7.906	7.914	7.923	942499	49.61	423.0932	DBSearch	Negative	Find by Auto I	1_050_65_N1001.d	99.21	424.1006	-0.6	-0.25	0	
73	Pyrocatechol	C6 H6 O2	Cpd 73: Pyrocatechol; C6 H6 O2; 8.015	109.0296	110.0369	8.006	8.015	8.023	318357	88.64	109.0294	DBSearch-Lib	Negative	Find by Auto I	1_050_65_N1001.d	87.68	110.0368	-0.67	-0.07	89.6	D:\MassHunter\
74	2,6-dihydroxybenzoic acid	C7 H6 O4	Cpd 74: 2,6-dihydroxybenzoic acid; C7 H6 O4; 8.040	153.0192	154.0265	8.031	8.04	8.048	139922	49.72	136.9367	DBSearch	Negative	Find by Auto I	1_050_65_N1001.d	99.45	154.0266	0.98	0.15	0	
77	Cynaroside A	C21 H32 O10	Cpd 77: Cynaroside A; C21 H32 O10; 8.441	443.1919	444.1991	8.433	8.441	8.449	129517	49.56	443.192	DBSearch	Negative	Find by Auto I	1_050_65_N1001.d	99.12	444.1995	1.02	0.45	0	
81	(-)-epicatechin-3'-O-glucuronide	C21 H24 O11	Cpd 81: (-)-epicatechin-3'-O-glucuronide; C21 H24 O11; 8.742	451.1235	452.1306	8.734	8.742	8.751	66106	47.96	331.0823	DBSearch	Negative	Find by Auto I	1_050_65_N1001.d	95.93	452.1319	2.72	1.23	0	
88	Chlorogenic Acid	C16 H18 O9	Cpd 88: Chlorogenic Acid; C16 H18 O9; 9.345	353.0875	354.0948	9.336	9.345	9.353	61958	99.06	191.0558	DBSearch-Lib	Negative	Find by Auto I	1_050_65_N1001.d	98.74	354.0951	0.93	0.33	99.38	D:\MassHunter\
90	3,4-Dihydroxybenzaldehyde	C7 H6 O3	Cpd 90: 3,4-Dihydroxybenzaldehyde; C7 H6 O3; 9.521	137.024	138.0314	9.512	9.521	9.529	150464	88.87	137.0238	DBSearch-Lib	Negative	Find by Auto I	1_050_65_N1001.d	86.47	138.0317	2.45	0.34	91.27	D:\MassHunter\
93	(±)-Catechin	C15 H14 O6	Cpd 93: (±)-Catechin; C15 H14 O6; 9.684	289.0718	290.0791	9.613	9.684	9.755	2280708	95.94	289.0718	DBSearch-Lib	Negative	Find by Auto I	1_050_65_N1001.d	99.18	290.079	-0.24	-0.07	92.69	D:\MassHunter\
96	Procyanidin B2	C30 H26 O12	Cpd 96: Procyanidin B2; C30 H26 O12; 10.073	577.1349	578.1422	10.014	10.073	10.131	4812543	90.63	577.1353	DBSearch-Lib	Negative	Find by Auto I	1_050_65_N1001.d	99.4	578.1424	0.47	0.27	81.87	D:\MassHunter\
98	Verbasoside	C20 H30 O12	Cpd 98: Verbasoside; C20 H30 O12; 10.223	461.1659	462.1731	10.215	10.223	10.232	183833	49.13	415.1609	DBSearch	Negative	Find by Auto I	1_050_65_N1001.d	98.27	462.1737	1.44	0.66	0	
100	Khellol glucoside	C19 H20 O10	Cpd 100: Khellol glucoside; C19 H20 O10; 10.424	407.0981	408.1053	10.416	10.424	10.432	372160	49.54	245.0453	DBSearch	Negative	Find by Auto I	1_050_65_N1001.d	99.09	408.1056	0.77	0.32	0	
101	Coriandrone C	C13 H10 O5	Cpd 101: Coriandrone C; C13 H10 O5; 10.449	245.0456	246.0529	10.441	10.449	10.457	119554	49.81	245.0452	DBSearch	Negative	Find by Auto I	1_050_65_N1001.d	99.63	246.0528	-0.31	-0.08	0	
103	2-Methoxy-1,4-benzoquinone	C7 H6 O3	Cpd 103: 2-Methoxy-1,4-benzoquinone; C7 H6 O3; 10.675	137.0242	138.0316	10.666	10.675	10.683	148848	41.07	137.0241	DBSearch	Negative	Find by Auto I	1_050_65_N1001.d	82.14	138.0317	0.92	0.13	0	
104	Epicatechin	C15 H14 O6	Cpd 104: Epicatechin; C15 H14 O6; 10.675	289.0721	290.0794	10.617	10.675	10.734	6241071	93.2	289.0718	DBSearch-Lib	Negative	Find by Auto I	1_050_65_N1001.d	98.41	290.079	-1.09	-0.32	87.99	D:\MassHunter\
105	Marmesin	C14 H14 O4	Cpd 105: Marmesin; C14 H14 O4; 10.700	245.0818	246.0891	10.642	10.7	10.758	438728	89.66	245.0811	DBSearch-Lib	Negative	Find by Auto I	1_050_65_N1001.d	86.65	246.0892	0.37	0.09	92.68	D:\MassHunter\
107	Vanillin 4-sulfate	C9 H10 O6 S	Cpd 107: Vanillin 4-sulfate; C9 H10 O6 S; 10.851	245.0125	246.0199	10.842	10.851	10.859	73907	48.96	96.96	DBSearch	Negative	Find by Auto I	1_050_65_N1001.d	97.92	246.0198	-0.31	-0.08	0	
111	2-Acetyl-5,8-dihydroxy-3-methoxy-1,4-naphthoquinone	C13 H10 O6	Cpd 111: 2-Acetyl-5,8-dihydroxy-3-methoxy-1,4-naphthoquinone; C13 H10 O6	261.0406	262.0478	11.119	11.127	11.135	456140	49.79	261.0405	DBSearch	Negative	Find by Auto I	1_050_65_N1001.d	99.58	262.0477	-0.27	-0.07	0	
114	Epifisetinidol-(4beta->8)-catechin	C30 H26 O11	Cpd 114: Epifisetinidol-(4beta->8)-catechin; C30 H26 O11; 11.227	561.1399	562.147	11.219	11.227	11.236	349816	49.61	289.0714	DBSearch	Negative	Find by Auto I	1_050_65_N1001.d	99.21	562.1475	0.84	0.47	0	
118	4-O-8',5'-5''-Dehydrotriferulic acid	C30 H26 O12	Cpd 118: 4-O-8',5'-5''-Dehydrotriferulic acid; C30 H26 O12; 11.629	577.1342	578.1415	11.621	11.629	11.637	121610	49.08	577.1331	DBSearch	Negative	Find by Auto I	1_050_65_N1001.d	98.16	578.1424	1.55	0.89	0	
119	Garcimangosone D	C19 H20 O9	Cpd 119: Garcimangosone D; C19 H20 O9; 11.830	391.1031	392.1104	11.821	11.83	11.838	138598	49.59	229.0505	DBSearch	Negative	Find by Auto I	1_050_65_N1001.d	99.17	392.1107	0.91	0.36	0	
123	Amaroswerin	C29 H30 O14	Cpd 123: Amaroswerin; C29 H30 O14; 12.381	601.1585	602.1653	12.373	12.381	12.39	61266	45	601.1579	DBSearch	Negative	Find by Auto I	1_050_65_N1001.d	89.99	602.1636	-2.89	-1.74	0	
124	O-Demethylfonsecin	C14 H12 O6	Cpd 124: O-Demethylfonsecin; C14 H12 O6; 12.733	275.056	276.0632	12.725	12.733	12.742	247996	49.72	109.0294	DBSearch	Negative	Find by Auto I	1_050_65_N1001.d	99.43	276.0634	0.54	0.15	0	
125	Miscanthoside	C21 H22 O11	Cpd 125: Miscanthoside; C21 H22 O11; 12.846	449.1086	450.1156	12.75	12.846	12.942	271478	48.33	449.108	DBSearch	Negative	Find by Auto I	1_050_65_N1001.d	96.65	450.1162	1.3	0.58	0	
126	Streptonigrin	C25 H22 N4 O8	Cpd 126: Streptonigrin; C25 H22 N4 O8; 12.997	505.1381	506.1451	12.951	12.997	13.043	804022	41.99	505.1381	DBSearch	Negative	Find by Auto I	1_050_65_N1001.d	83.97	506.1438	-2.65	-1.34	0	
127	Citrusin B	C27 H36 O13	Cpd 127: Citrusin B; C27 H36 O13; 13.235	567.2076	568.2148	13.227	13.235	13.244	96703	48.78	179.0552	DBSearch	Negative	Find by Auto I	1_050_65_N1001.d	97.55	568.2156	1.42	0.81	0	
135	(R)-1-O-[b-D-Glucopyranosyl-(1->6)-b-D-glucopyranoside]-1,3-octaned	C20 H38 O12	Cpd 135: (R)-1-O-[b-D-Glucopyranosyl-(1->6)-b-D-glucopyranoside]-1,3-octan	469.2286	470.2358	14.03	14.038	14.047	154027	48.46	423.223	DBSearch	Negative	Find by Auto I	1_050_65_N1001.d	96.91	470.2363	1.13	0.53	0	
143	Urolithin D	C13 H8 O6	Cpd 143: Urolithin D; C13 H8 O6; 16.046	259.0245	260.0318	16.038	16.046	16.055	187017	49.58	259.0244	DBSearch	Negative	Find by Auto I	1_050_65_N1001.d	99.16	260.0321	1.02	0.27	0	
151	Coriandrin	C13 H10 O4	Cpd 151: Coriandrin; C13 H10 O4; 18.054	229.0508	230.058	18.046	18.054	18.062	553914	49.32	229.0507	DBSearch	Negative	Find by Auto I	1_050_65_N1001.d	98.63	230.0579	-0.6	-0.14	0	
155	Muscomin	C18 H18 O7	Cpd 155: Muscomin; C18 H18 O7; 19.008	345.0978	346.1051	18.949	19.008	19.066	552962	49.8	345.0978	DBSearch	Negative	Find by Auto I	1_050_65_N1001.d	99.61	346.1053	0.51	0.18	0	
160	(2S,4S,6S)-2-[2-(4-Hydroxy-3-meyhoxyphenyl)ethyl]tetrahydro-6-(4,5-di	C23 H28 O8	Cpd 160: (2S,4S,6S)-2-[2-(4-Hydroxy-3-meyhoxyphenyl)ethyl]tetrahydro-6-(4,5-	431.1705	432.1778	20.154	20.163	20.171	208679	49.1	431.1704	DBSearch	Negative	Find by Auto I	1_050_65_N1001.d	98.19	432.1784	1.46	0.63	0	
164	Cubebininolide	C24 H30 O8	Cpd 164: Cubebininolide; C24 H30 O8; 21.417	445.1861	446.1934	21.409	21.417	21.425	31483	48.32	445.185	DBSearch	Negative	Find by Auto I	1_050_65_N1001.d	96.64	446.1941	1.55	0.69	0	
169	Ugaxanthone	C18 H16 O6	Cpd 169: Ugaxanthone; C18 H16 O6; 24.781	327.0874	328.0947	24.772	24.781	24.789	360779	49.26	327.0869	DBSearch	Negative	Find by Auto I	1_050_65_N1001.d	98.52	328.0947	-0.06	-0.02	0	
171	Garcinone C	C23 H26 O7	Cpd 171: Garcinone C; C23 H26 O7; 26.989	413.1602	414.1674	26.981	26.989	26.998	227823	49.56	413.1598	DBSearch	Negative	Find by Auto I	1_050_65_N1001.d	99.11	414.1679	1.05	0.43	0	
172	1,4,6-Trihydroxy-5-methoxy-7-prenylxanthone	C19 H18 O6	Cpd 172: 1,4,6-Trihydroxy-5-methoxy-7-prenylxanthone; C19 H18 O6; 27.441	341.1025	342.1099	27.432	27.441	27.449	28204	49.31	341.1018	DBSearch	Negative	Find by Auto I	1_050_65_N1001.d	98.62	342.1103	1.42	0.48	0	
173	1-Isomangostin hydrate	C24 H28 O7	Cpd 173: 1-Isomangostin hydrate; C24 H28 O7; 27.993	427.1759	428.1831	27.985	27.993	28.002	317215	49.52	427.1752	DBSearch	Negative	Find by Auto I	1_050_65_N1001.d	99.04	428.1835	1	0.43	0	
178	Garcinone A	C23 H24 O5	Cpd 178: Garcinone A; C23 H24 O5; 33.364	379.1549	380.1621	33.306	33.364	33.423	580157	49.81	379.1551	DBSearch	Negative	Find by Auto I	1_050_65_N1001.d	99.62	380.1624	0.6	0.23	0	
179	Dulciol C	C28 H34 O7	Cpd 179: Dulciol C; C28 H34 O7; 33.515	481.2223	482.2295	33.507	33.515	33.523	106774	48.38	481.2222	DBSearch	Negative	Find by Auto I	1_050_65_N1001.d	96.77	482.2305	2.04	0.99	0	
181	BR-Xanthone A	C23 H24 O6	Cpd 181: BR-Xanthone A; C23 H24 O6; 34.669	395.1499	396.1571	34.611	34.669	34.728	1013065	49.68	395.1498	DBSearch	Negative	Find by Auto I	1_050_65_N1001.d	99.36	396.1573	0.45	0.18	0	
183	α-Mangostin	C24 H26 O6	Cpd 183: α-Mangostin; C24 H26 O6; 35.121	409.1657	410.1729	35.012	35.121	35.23	1673642	99.29	409.1658	DBSearch-Lib	Negative	Find by Auto I	1_050_65_N1001.d	99.64	410.1729	0.03	0.01	98.94	D:\MassHunter\
188	Dulciol B	C28 H32 O6	Cpd 188: Dulciol B; C28 H32 O6; 35.987	463.2124	464.2196	35.916	35.987	36.058	589186	49.72	463.2122	DBSearch	Negative	Find by Auto I	1_050_65_N1001.d	99.45	464.2199	0.58	0.27	0	
