# Supplementary material for: Mapping the Polar Neuro-Interactome of Garcinia mangostana Against the AD-PD-ALS Nexus
Source: Life (Basel). 2026 Apr 1;16(4):580. doi: 10.3390/life16040580 (PMC13117457; doi:10.3390/life16040580)
Supplement: Supplementary file 1 [file life-16-00580-s001.zip › Supplementary file S3_Compounds selction.pdf]

S3: compound selection framework for GMPE metabolites

Molecule name	Molecule	Canonical SMILES	Formula	MW	#H-bond acceptors	#H-bond donors	TPSA	iLOGP	XLOGP3	WLOGP	MLOGP	Silicos-IT Lo	Consensus Log P
Quinic acid	Molecule 1	OC1[C@H](O)CC(C[C@H]1O)(O)C(=O)O	C7H12O6	192.17	6	5	118.22	0.22	-2.37	-2.32	-2.14	-1.82	-1.69
Epicatechin	Molecule 2	C1[C@H]([C@H](OC2=CC(=CC(=C21)O)O)C3=CC(=C(C=C3)O)O)O	C15H14O6	290.27	6	5	110.38	1.47	0.36	1.22	0.24	0.98	0.85
Procyanidin B2	Molecule 3	Oc1cc(O)c2c(c1)O[C@@H]([C@@H]([C@H]2c1c(O)cc(c2c1O[C@@H]([C@@H](C2)O)c1ccc(c(c1)O)O)O)O)c1ccc(c(c1)O)O	C30H26O12	578.52	12	10	220.76	1.35	2.37	2.35	-0.26	1.14	1.39
Galactaric acid	Molecule 4	O[C@H]([C@H](C(=O)O)O)[C@H]([C@@H](C(=O)O)O)O	C6H10O8	210.14	8	6	155.52	-0.62	-2.51	-3.4	-2.98	-2.61	-2.43
Sucrose	Molecule 5	OC[C@H]1O[C@@]([C@H]([C@@H]1O)O)(CO)O[C@H]1O[C@H](CO)[C@H]([C@@H]([C@H]1O)O)O	C12H22O11	342.3	11	8	189.53	0.85	-3.7	-5.4	-4.37	-3.86	-3.29
(±)-Catechin	Molecule 6	C1[C@@H]([C@H](OC2=CC(=CC(=C21)O)O)C3=CC(=C(C=C3)O)O)O	C15H14O6	290.27	6	5	110.38	1.47	0.36	1.22	0.24	0.98	0.85
α-Mangostin	Molecule 7	COc1c(O)cc2c(c1CC=C(C)C)c(=O)c1c(o2)cc(c(c1O)CC=C(C)C)O	C24H26O6	410.46	6	3	100.13	4.14	6.27	5.09	2.19	5.52	4.64
Citric acid	Molecule 8	OC(=O)C(CC(=O)O)(CC(=O)O)O	C6H8O7	192.12	7	4	132.13	-1.49	-1.72	-1.25	-1.48	-1.6	-1.51
Marmesin	Molecule 9	O=c1ccc2c(o1)cc1c(c2)C[C@H](O1)C(O)(C)C	C14H14O4	246.26	4	1	59.67	2.55	1.91	1.87	1.6	2.78	2.14
Chlorogenic Acid	Molecule 10	O=C(O[C@@H]1C[C@@](O)(C[C@H]([C@H]1O)O)C(=O)O)/C=C/c1ccc(c(c1)O)O	C16H18O9	354.31	9	6	164.75	0.87	-0.42	-0.75	-1.05	-0.61	-0.39
Palmitic amide	Molecule 11	CCCCCCCCCCCCCCCC(=O)N	C16H33NO	255.44	1	1	43.09	3.87	6.52	4.95	3.79	5.01	4.83
6-(alpha-D-Glucosaminyl)-1D-myo-inositol	Molecule 12	OC[C@H]1O[C@H](OC2[C@H](O)[C@H](O)C([C@H]([C@@H]2O)O)O)[C@@H]([C@H]([C@@H]1O)O)N	C12H23NO10	341.31	11	9	206.32	0.11	-5.95	-6.04	-4.77	-5.28	-4.39
D-Galactopyranosyl-(1->3)-D-galactopyranosyl-(1->3)-L-arabinose	Molecule 13	OCC1OC(OC2C(O)COC(C2O)O)C(C(C1O)OC1OC(CO)C(C(C1O)O)O)O	C17H30O15	474.41	15	10	248.45	-0.75	-5.71	-6.93	-5.66	-6	-5.01
Trigonelline	Molecule 14	C[n+]1cccc(c1)C(=O)[O-]	C7H7NO2	137.14	2	0	44.01	-3.11	0.51	-1.13	0.33	0.36	-0.61
α-D-Glucose	Molecule 15	C([C@@H]1[C@H]([C@@H]([C@H]([C@H](O1)O)O)O)O)O	C15H10O4	180.16	4	2	70.67	1.77	2.47	2.87	1.08	3.02	2.24
Choline	Molecule 16	OCC[N+](C)(C)C	C5H14NO+	104.17	1	1	20.23	-2.14	-0.4	-0.32	-3.46	-3	-1.86
Kojic Acid	Molecule 17	OCc1occ(c(=O)c1)O	C6H6O4	142.11	4	2	70.67	1.12	-0.64	-0.31	-1.69	0.74	-0.16

Molecule name	ESOL Log S	ESOL Solubi	ESOL Solubi	ESOL Class	Ali Class	Silicos-IT Lo	Silicos-IT So	Silicos-IT So	Silicos-IT cl	GI absorpti	BBB perme	Pgp substra	CYP1A2 inh	CYP2C19 in	CYP2C9 inh	CYP2D6 inh	CYP3A4 inh	log Kp (cm/s)	Lipinski #viola	Ghose #viol	Veber #viol	Egan #viola	Muegge #vi	Bioavailabil	PAINS #aler	Brenk #aler	Leadlikeness #	Justification
Quinic acid	0.53	6.48E+02	3.37E+00	Highly solut	Highly solut	2.08	2.30E+04	1.20E+02	Soluble	Low	No	No	No	No	No	No	No	-9.15	0	1	0	0	2	0.56	0	0	1	Low GI + no neuroactive precedent
Epicatechin	-2.22	1.74E+00	5.98E-03	Soluble	Soluble	-2.14	2.09E+00	7.19E-03	Soluble	High	No	Yes	No	No	No	No	No	-7.82	0	0	0	0	0	0.55	1	1	0	High abundance + AChE inhibition evidence
Procyanidin B2	-5.14	4.15E-03	7.17E-06	Moderately	Poorly solut	-3.91	7.05E-02	1.22E-04	Soluble	Low	No	No	No	No	No	No	Yes	-8.15	3	2	1	1	3	0.17	1	1	1	Low GI + 3 Lipinski violations + MW 578 Da
Galactaric acid	0.77	1.23E+03	5.87E+00	Highly solut	Very solubl	3.47	6.22E+05	2.96E+03	Soluble	Low	No	No	No	No	No	No	No	-9.36	1	2	1	1	3	0.11	0	0	1	Low GI + no neuroactive target relevance
Sucrose	0.7	1.71E+03	5.00E+00	Highly solut	Highly solut	3.67	1.62E+06	4.73E+03	Soluble	Low	No	Yes	No	No	No	No	No	-11.02	2	1	1	1	4	0.17	0	0	0	Low GI + 2 Lipinski violations
(±)-Catechin	-2.22	1.74E+00	5.98E-03	Soluble	Soluble	-2.14	2.09E+00	7.19E-03	Soluble	High	No	Yes	No	No	No	No	No	-7.82	0	0	0	0	0	0.55	1	1	0	High abundance + Nrf2/BDNF neuroactive evidence
α-Mangostin	-6.35	1.83E-04	4.46E-07	Poorly solul	Poorly solut	-6.14	2.97E-04	7.23E-07	Poorly solul	High	No	No	No	No	Yes	No	No	-4.35	0	0	0	0	1	0.55	0	2	2	Established neuroprotective reference compound
Citric acid	0.38	4.63E+02	2.41E+00	Highly solut	Very solubl	1.71	9.90E+03	5.15E+01	Soluble	Low	No	No	No	No	No	No	No	-8.69	0	2	0	1	1	0.56	0	0	1	Low GI + no direct neuroactive relevance
Marmesin	-2.92	2.99E-01	1.22E-03	Soluble	Soluble	-3.93	2.92E-02	1.19E-04	Soluble	High	Yes	No	Yes	No	No	No	No	-6.45	0	0	0	0	0	0.55	0	1	1	Primary CNS Leads
Chlorogenic Acid	-1.62	8.50E+00	2.40E-02	Very solubl	Soluble	0.4	8.94E+02	2.52E+00	Soluble	Low	No	No	No	No	No	No	No	-8.76	1	1	1	1	2	0.11	1	2	1	Low GI + 1 Lipinski violation
Palmitic amide	-4.61	6.31E-03	2.47E-05	Moderately	Poorly solut	-5.53	7.57E-04	2.96E-06	Moderately	High	Yes	No	Yes	No	No	No	No	-3.23	0	0	1	0	1	0.55	0	0	2	Primary CNS Leads
6-(alpha-D-Glucosaminyl)-1D-myo-inositol	1.99	3.34E+04	9.78E+01	Highly solut	Highly solut	4.71	1.73E+07	5.08E+04	Soluble	Low	No	Yes	No	No	No	No	No	-12.61	2	1	1	1	4	0.17	0	0	0	Low GI + 2 Lipinski violations
D-Galactopyranosyl-(1->3)-D-galactopyranosyl-(1->3)-L-arabinose	1.21	7.73E+03	1.63E+01	Highly solut	Highly solut	5.57	1.76E+08	3.72E+05	Soluble	Low	No	Yes	No	No	No	No	No	-13.25	2	1	1	1	4	0.17	0	0	1	Low GI + MW 474 Da + 2 violations
Trigonelline	-1.39	5.59E+00	4.08E-02	Very solubl	Very solubl	-0.94	1.59E+01	1.16E-01	Soluble	High	No	No	No	No	No	No	No	-6.77	0	4	0	0	1	0.55	0	1	1	MAOB inhibition + Aβ neuroprotection evidence
α-D-Glucose	-3.53	7.51E-02	2.95E-04	Soluble	Soluble	-4.98	2.64E-03	1.04E-05	Moderately	High	Yes	No	Yes	No	No	Yes	Yes	-6.1	0	0	0	0	0	0.55	0	0	0	Antioxidant + dopaminergic pathway relevance
Choline	-0.1	8.24E+01	7.91E-01	Very solubl	Highly solut	-1.26	5.74E+00	5.51E-02	Soluble	Low	No	No	No	No	No	No	No	-7.22	0	2	0	0	1	0.55	0	1	1	Ionized zwitterion + Low GI
Kojic Acid	-0.7	2.86E+01	2.01E-01	Very solubl	Very solubl	-1.17	9.72E+00	6.84E-02	Soluble	High	No	No	No	No	No	No	No	-7.62	0	3	0	0	1	0.55	0	0	1	Toxicity class (III)

All 17 GMPE metabolites identified by UHPLC-QTOF-MS
↓
TIER 1: BBB-Permeant Leads
(BBB = Yes + High GI + Lipinski = 0)
→ Palmitic amide (Molecule 11)
→ Marmesin (Molecule 9)
↓
TIER 2: Mechanistically Relevant Secondary Panel
(High GI + Lipinski = 0 + established
neuroactive literature evidence)
→ α-Mangostin (Molecule 7)
→ Epicatechin (Molecule 2)
→ (±)-Catechin (Molecule 6)
→ Trigonelline (Molecule 14)
→ Kojic Acid (Molecule 17)
↓
TIER 3: Benchmark/Comparative Reference
(endogenous CNS-relevant metabolite)
→ α-D-Glucose (Molecule 15)
↓
EXCLUDED (Molecules 1,3,4,5,8,10,12,13,16)
Low GI + BBB = No + Lipinski violations
