# Supplementary material for: Mapping the Polar Neuro-Interactome of Garcinia mangostana Against the AD-PD-ALS Nexus
Source: Life (Basel). 2026 Apr 1;16(4):580. doi: 10.3390/life16040580 (PMC13117457; doi:10.3390/life16040580)
Supplement: Supplementary file 1 [file life-16-00580-s001.zip › Supplementary file S4_Targets of AD,PD,ALS 2.pdf]

S4: AD, PD, and ALS, targets from the GeneCards, DisGeNET, and OMIM databases

	AD Genes	AD Genes (After remove duplicate)	PD Genes	PD Genes (After remove duplicate)	ALS Genes	ALS Genes (After remove duplicate)	976 (AD, PD, ALS)	Compounds and Diseases (AD, PD, ALS) (121)
GeneCards (GIFtS) ≥ 60	MTOR	MTOR	MTOR	MTOR	MTOR	MTOR	MTOR	CA2
	ERBB2	ERBB2	ERBB2	ERBB2	IGF1R	IGF1R	ERBB2	CA1
	IGF1R	IGF1R	IGF1R	IGF1R	AKT3	AKT3	IGF1R	HDAC1
	AKT3	AKT3	AKT3	AKT3	ERBB2	ERBB2	AKT3	HDAC3
	RET	RET	RET	RET	ERBB4	ERBB4	RET	HDAC6
	EGFR	EGFR	EGFR	EGFR	CREBBP	CREBBP	EGFR	HDAC2
	BRAF	BRAF	BRAF	BRAF	EGFR	EGFR	BRAF	FAAH
	ERBB4	ERBB4	ERBB4	ERBB4	FGFR1	FGFR1	ERBB4	CHRNA4
	ESR1	ESR1	PTPN11	PTPN11	BRAF	BRAF	ESR1	PLAU
	FGFR3	FGFR3	ESR1	ESR1	FGFR3	FGFR3	FGFR3	ASAH1
	PTPN11	PTPN11	CREBBP	CREBBP	MAP2K2	MAP2K2	PTPN11	TEK
	FGFR1	FGFR1	FGFR3	FGFR3	FGFR2	FGFR2	FGFR1	CA9
	MAP2K1	MAP2K1	FGFR1	FGFR1	ESR1	ESR1	MAP2K1	AR
	CREBBP	CREBBP	RAF1	RAF1	PTPN11	PTPN11	CREBBP	HMGCR
	RAF1	RAF1	MAP2K1	MAP2K1	MAP2K1	MAP2K1	RAF1	JAK1
	FGFR2	FGFR2	KDR	KDR	RAF1	RAF1	FGFR2	JAK2
	MAP2K2	MAP2K2	FGFR2	FGFR2	MET	MET	MAP2K2	JAK3
	KDR	KDR	CHEK2	CHEK2	CHEK2	CHEK2	KDR	CSNK1D
	CHEK2	CHEK2	MAP2K2	MAP2K2	RET	RET	CHEK2	TYK2
	ERBB3	ERBB3	MET	MET	KDR	KDR	ERBB3	CSNK1E
	MET	MET	ERBB3	ERBB3	ERBB3	ERBB3	MET	MAPK14
	PSEN1	PSEN1	PSEN1	PSEN1	SOD1	SOD1	PSEN1	PIM1
	NOTCH1	NOTCH1	SOD1	SOD1	PSEN1	PSEN1	NOTCH1	MAPKAPK2
	TP53	TP53	TP53	TP53	TP53	TP53	TP53	MPO
	SOD1	SOD1	STAT3	STAT3	DNMT1	DNMT1	SOD1	TRPV1
	STAT3	STAT3	NOTCH1	NOTCH1	NTRK2	NTRK2	STAT3	PARP1
	CTNNB1	CTNNB1	ATM	ATM	PDGFRB	PDGFRB	CTNNB1	RPS6KB1
	PDGFRB	PDGFRB	CFTR	CFTR	CTNNB1	CTNNB1	PDGFRB	AURKA
	ADAM10	ADAM10	PDGFRB	PDGFRB	MMP9	MMP9	ADAM10	LRRK2
	CFTR	CFTR	DNMT1	DNMT1	STAT3	STAT3	CFTR	HIF1A
	MMP9	MMP9	CTNNB1	CTNNB1	AKT1	AKT1	MMP9	PIK3CA
	SLC2A1	SLC2A1	MMP9	MMP9	CDK5	CDK5	SLC2A1	GSK3B
	AKT1	AKT1	AKT1	AKT1	MAPK1	MAPK1	AKT1	FKBP1A
	KRAS	KRAS	SLC2A1	SLC2A1	ATM	ATM	KRAS	COMT
	CDK5	CDK5	KIT	KIT	INSR	INSR	CDK5	TLR7
	NFKB1	NFKB1	NFKB1	NFKB1	HDAC6	HDAC6	NFKB1	NR3C1
	SMAD4	SMAD4	KRAS	KRAS	CFTR	CFTR	SMAD4	PSEN2
	ATM	ATM	JAK2	JAK2	ADAM10	ADAM10	ATM	KIF11
	SMAD3	SMAD3	SMAD4	SMAD4	NFKB1	NFKB1	SMAD3	CDK1
	KIT	KIT	SMAD3	SMAD3	NOTCH1	NOTCH1	KIT	BRD4
	MAPK1	MAPK1	MMP2	MMP2	SLC2A1	SLC2A1	MAPK1	MAP3K5
	INSR	INSR	CDK5	CDK5	SMAD3	SMAD3	INSR	CREBBP
	DNMT1	DNMT1	NTRK2	NTRK2	MMP2	MMP2	DNMT1	IGF1R
	JAK2	JAK2	MAPK1	MAPK1	IKBKB	IKBKB	JAK2	KDR
	MMP2	MMP2	EZH2	EZH2	ABL1	ABL1	MMP2	FGFR1
	NTRK2	NTRK2	ADAM10	ADAM10	KRAS	KRAS	NTRK2	ERBB2
	PIK3CD	PIK3CD	ABL1	ABL1	PIK3CD	PIK3CD	PIK3CD	EGFR
	IKBKB	IKBKB	BTK	BTK	HDAC2	HDAC2	IKBKB	ERBB4
	EZH2	EZH2	INSR	INSR	TGFBR1	TGFBR1	EZH2	IMPDH2
	ABL1	ABL1	PIK3CD	PIK3CD	SMAD4	SMAD4	ABL1	MAP2K1
	HDAC6	HDAC6	IKBKB	IKBKB	EZH2	EZH2	HDAC6	CSNK2A1
	TGFBR1	TGFBR1	HDAC6	HDAC6	ATR	ATR	BTK	ADA
	BTK	BTK	JAK1	JAK1	BTK	BTK	AKT2	EIF2AK3
	AKT2	AKT2	PDGFRA	PDGFRA	AKT2	AKT2	JAK1	AHCY
	JAK1	JAK1	MDM2	MDM2	PDGFRA	PDGFRA	FLT3	PRKCE
	FLT3	FLT3	CDK4	CDK4	CDK4	CDK4	CDK4	PRKCQ
	CDK4	CDK4	FLT3	FLT3	JAK2	JAK2	PDGFRA	PNP
	PDGFRA	PDGFRA	TYK2	TYK2	JAK1	JAK1	NTRK3	MAOA
	NTRK3	NTRK3	ATR	ATR	KIT	KIT	TYK2	CXCR2
	TYK2	TYK2	NTRK3	NTRK3	TYK2	TYK2	MDM2	GABRB3
	MDM2	MDM2	AKT2	AKT2	CHEK1	CHEK1	ATR	ALDH2
	ATR	ATR	EPHB4	EPHB4	MDM2	MDM2	EPHB4	ESR1
	EPHB4	EPHB4	HDAC2	HDAC2	NTRK3	NTRK3	HDAC2	ESR2
	HDAC2	HDAC2	CHEK1	CHEK1	EPHB4	EPHB4	CHEK1	TBXAS1
	CHEK1	CHEK1	PTEN	PTEN	FLT3	FLT3	PTEN	HTR2A
	PTEN	PTEN	UCHL1	UCHL1	PTEN	PTEN	MPO	ADORA2A
	MPO	MPO	TGFB1	TGFB1	UCHL1	UCHL1	TGFB1	ESRRB
	TGFB1	TGFB1	NOTCH2	NOTCH2	MPO	MPO	UCHL1	ABCG2
	UCHL1	UCHL1	MPO	MPO	TGFB1	TGFB1	CTSD	CYP19A1
	CTSD	CTSD	AR	AR	CTSD	CTSD	NOTCH2	TYR
	NOTCH2	NOTCH2	CTSD	CTSD	CXCR4	CXCR4	AR	MIF
	AR	AR	IDH1	IDH1	STAT1	STAT1	PPARG	XDH
	PPARG	PPARG	STAT1	STAT1	DNMT3A	DNMT3A	STAT1	ABCB1
	STAT1	STAT1	CXCR4	CXCR4	PPARG	PPARG	CXCR4	PTGS1
	CXCR4	CXCR4	PPARG	PPARG	EP300	EP300	AXL	SLC6A2
	AXL	AXL	MYC	MYC	MYC	MYC	TGFBR2	MAOB
	TGFBR2	TGFBR2	DNMT3A	DNMT3A	GRIN2B	GRIN2B	GRIN2B	ACHE
	GRIN2B	GRIN2B	TGFBR2	TGFBR2	AR	AR	DNMT3A	ALOX5
	DNMT3A	DNMT3A	CCND1	CCND1	CAMK2A	CAMK2A	MYC	PTPN1
	MYC	MYC	IDH2	IDH2	PRKACA	PRKACA	EP300	PLAT
	EP300	EP300	GRIN2B	GRIN2B	TGFBR2	TGFBR2	CCND1	MCL1
	CCND1	CCND1	EP300	EP300	NOTCH2	NOTCH2	PRKACA	PPARG
	PRKACA	PRKACA	PRKACA	PRKACA	IDH2	IDH2	IDH2	IDH1
	IDH2	IDH2	PRKCD	PRKCD	IDH1	IDH1	IDH1	FASN
	IDH1	IDH1	AXL	AXL	RPS6KA3	RPS6KA3	CAMK2A	NFKB1
	PRKCD	PRKCD	NT5E	NT5E	CCND1	CCND1	RPS6KA3	PTGS2
	MYLK	MYLK	NFKB2	NFKB2	AXL	AXL	NFKB2	RELA
	CAMK2A	CAMK2A	CAMK2A	CAMK2A	NT5E	NT5E	NT5E	HSP90B1
	RPS6KA3	RPS6KA3	RPS6KA3	RPS6KA3	AURKB	AURKB	AURKB	HSP90AA1
	NFKB2	NFKB2	AURKB	AURKB	NFKB2	NFKB2	SNCA	SRC
	NT5E	NT5E	SNCA	SNCA	TSC2	TSC2	ACE	HSP90AB1
	AURKB	AURKB	TERT	TERT	NOTCH3	NOTCH3	TERT	CTNNB1
	SNCA	SNCA	ACE	ACE	SNCA	SNCA	PLAU	SIRT1
	ACE	ACE	HSPB1	HSPB1	CSF1R	CSF1R	NOTCH3	AGTR1
	TERT	TERT	PIK3CA	PIK3CA	ACE	ACE	PIK3CA	TNF
	PLAU	PLAU	NOTCH3	NOTCH3	PLAU	PLAU	CSF1R	BCL2
	NOTCH3	NOTCH3	CSF1R	CSF1R	PRKCG	PRKCG	HSPB1	KIT
	PIK3CA	PIK3CA	TSC2	TSC2	HSPB1	HSPB1	TSC2	MET
	CSF1R	CSF1R	HRAS	HRAS	NGF	NGF	GSK3B	MMP2
	HSPB1	HSPB1	HMOX1	HMOX1	NFE2L2	NFE2L2	LDLR	MMP9
	TSC2	TSC2	LDLR	LDLR	GRIA2	GRIA2	HMOX1	MAPT
	GSK3B	GSK3B	NFE2L2	NFE2L2	BCL2	BCL2	HRAS	DNMT1
	LDLR	LDLR	TLR2	TLR2	PIK3CA	PIK3CA	NGF	DYRK1A
	HMOX1	HMOX1	GSK3B	GSK3B	CASP8	CASP8	TLR2	KCNH2
	HRAS	HRAS	PLAU	PLAU	MAPK14	MAPK14	NFE2L2	APP
	NGF	NGF	NGF	NGF	HDAC4	HDAC4	BCL2	TERT
	TLR2	TLR2	PRKCG	PRKCG	PTPRC	PTPRC	CASP8	STAT1
	NFE2L2	NFE2L2	ABCB1	ABCB1	ADA	ADA	ABCB1	GABRA1
	BCL2	BCL2	BCL2	BCL2	GSK3B	GSK3B	ADA	CYP1B1
	CASP8	CASP8	ADA	ADA	TLR2	TLR2	PIK3R1	FLT1
	ABCB1	ABCB1	CASP8	CASP8	EGF	EGF	AGTR1	MMP14
	ADA	ADA	AGTR1	AGTR1	SMAD2	SMAD2	TF	MMP1
	PIK3R1	PIK3R1	PTPRC	PTPRC	GRIN2A	GRIN2A	SMAD2	MMP7
	AGTR1	AGTR1	SMAD2	SMAD2	HRAS	HRAS	GAPDH	ABCC1
	TF	TF	TF	TF	HMOX1	HMOX1	RELA	PLA2G7
	SMAD2	SMAD2	SYK	SYK	DBH	DBH	SYK	CDK5
	GAPDH	GAPDH	JAK3	JAK3	HSP90AA1	HSP90AA1	PTPRC	NCSTN
	RELA	RELA	RELA	RELA	GAPDH	GAPDH	JAK3	PSEN1
	SYK	SYK	PIK3R1	PIK3R1	PLCG2	PLCG2	PLCG2	GABRG2
	PTPRC	PTPRC	EGF	EGF	FOXO1	FOXO1	ALK	TNNT2
	JAK3	JAK3	PARP1	PARP1	HDAC1	HDAC1	EGF	TNNI3
	PLCG2	PLCG2	ALK	ALK	FOS	FOS	GRIN2A	
	ALK	ALK	DBH	DBH	PARP1	PARP1	SOS1	
	EGF	EGF	GAPDH	GAPDH	SYK	SYK	PTCH1	
	GRIN2A	GRIN2A	HDAC4	HDAC4	TERT	TERT	DBH	
	SOS1	SOS1	SOS1	SOS1	CALR	CALR	CALR	
	PTCH1	PTCH1	ITK	ITK	PTCH1	PTCH1	PARP1	
	DBH	DBH	PTCH1	PTCH1	CDK6	CDK6	PRKCG	
	CALR	CALR	PLCG2	PLCG2	CBL	CBL	CBL	
	PARP1	PARP1	HGF	HGF	ABCB1	ABCB1	HDAC4	
	PRKCG	PRKCG	CALR	CALR	SOS1	SOS1	IFNGR1	
	CBL	CBL	IFNGR1	IFNGR1	HGF	HGF	BCR	
	ITGB3	ITGB3	FOXO1	FOXO1	ALK	ALK	HGF	
	HDAC4	HDAC4	CBL	CBL	PIK3R1	PIK3R1	MAPK14	
	IFNGR1	IFNGR1	STAT6	STAT6	RAD51	RAD51	HSP90AA1	
	BCR	BCR	MAPK14	MAPK14	FLT1	FLT1	HDAC1	
	HGF	HGF	GRIN2A	GRIN2A	PRKCA	PRKCA	ITK	
	MAPK14	MAPK14	HSP90AA1	HSP90AA1	BCR	BCR	FOXO1	
	HSP90AA1	HSP90AA1	RAD51	RAD51	JAK3	JAK3	CDK6	
	HDAC1	HDAC1	HDAC1	HDAC1	LDLR	LDLR	FLT1	
	ITK	ITK	BMPR1A	BMPR1A	AGTR1	AGTR1	CDH2	
	FOXO1	FOXO1	DPYD	DPYD	TF	TF	STAT6	
	CDK6	CDK6	CDK6	CDK6	BMPR1A	BMPR1A	RAD51	

FLT1	FLT1	FLT1	FLT1	TEK	TEK	BMPR1A
CDH2	CDH2	BCR	BCR	SMO	SMO	PRKCA
STAT6	STAT6	TEK	TEK	ITK	ITK	LCK
RAD51	RAD51	FOS	FOS	ITGB3	ITGB3	GRIA2
BMPR1A	BMPR1A	SMO	SMO	IFNGR1	IFNGR1	TEK
PRKCA	PRKCA	CDH2	CDH2	RELA	RELA	FOS
FLT4	FLT4	PRKCA	PRKCA	FLT4	FLT4	SMO
DPYD	DPYD	GRIA2	GRIA2	STAT6	STAT6	AURKA
LCK	LCK	PROC	PROC	CDH2	CDH2	EPHA2
GRIA2	GRIA2	AURKA	AURKA	LCK	LCK	EPHB2
TEK	TEK	EPHA2	EPHA2	PLK1	PLK1	PLK1
FOS	FOS	EPHB2	EPHB2	EPHB2	EPHB2	APP
SMO	SMO	PLK1	PLK1	AURKA	AURKA	PSEN2
PROC	PROC	GAA	GAA	EPHA2	EPHA2	GAA
FGFR4	FGFR4	APP	APP	APP	APP	LMNA
AURKA	AURKA	LMNA	LMNA	PSEN2	PSEN2	TNF
EPHA2	EPHA2	IL6	IL6	GNAS	GNAS	IL6
EPHB2	EPHB2	PSEN2	PSEN2	CBS	CBS	DNM2
PLK1	PLK1	SLC6A3	SLC6A3	PRKAR1A	PRKAR1A	SLC6A3
APP	APP	DNM2	DNM2	COMT	COMT	ADAM17
PSEN2	PSEN2	TH	TH	IFNG	IFNG	TLR4
GAA	GAA	TNF	TNF	TNF	TNF	TH
LMNA	LMNA	COMT	COMT	IL6	IL6	ATP2A2
TNF	TNF	IFNG	IFNG	LMNA	LMNA	CBS
IL6	IL6	TLR4	TLR4	GJA1	GJA1	IFNG
DNM2	DNM2	F2	F2	TH	TH	FAS
SLC6A3	SLC6A3	CBS	CBS	BAX	BAX	GATA3
ADAM17	ADAM17	ADAM17	ADAM17	NOS1	NOS1	PTGS2
TLR4	TLR4	ATP2A2	ATP2A2	SHH	SHH	COMT
TH	TH	GATA3	GATA3	TUBB3	TUBB3	JAG1
ATP2A2	ATP2A2	ALPL	ALPL	ACTB	ACTB	XIAP
CBS	CBS	JAG1	JAG1	TLR4	TLR4	PDGFB
IFNG	IFNG	SCN5A	SCN5A	GRIA3	GRIA3	AIFM1
FAS	FAS	XIAP	XIAP	SLC6A3	SLC6A3	LPL
GATA3	GATA3	BMPR2	BMPR2	GLUD1	GLUD1	BRCA1
PTGS2	PTGS2	PDGFB	PDGFB	IL2RA	IL2RA	SCN5A
COMT	COMT	FAS	FAS	EIF2AK3	EIF2AK3	NRAS
JAG1	JAG1	HK1	HK1	GRM1	GRM1	BCHE
XIAP	XIAP	GNAS	GNAS	PTGS2	PTGS2	EIF2AK3
PDGFB	PDGFB	BRCA1	BRCA1	BRCA1	BRCA1	F2
AIFM1	AIFM1	AIFM1	AIFM1	FN1	FN1	SERPINE1
EDNRB	EDNRB	PTGS2	PTGS2	DNM2	DNM2	ALPL
LPL	LPL	MMP1	MMP1	GRIN1	GRIN1	MMP1
BRCA1	BRCA1	DRD2	DRD2	CAT	CAT	CDKN2A
SCN5A	SCN5A	KCNH2	KCNH2	PRKDC	PRKDC	SLC2A2
NRAS	NRAS	PRKAR1A	PRKAR1A	CD4	CD4	IL2RA
BCHE	BCHE	SERPINE1	SERPINE1	AIFM1	AIFM1	GNAS
EIF2AK3	EIF2AK3	CDKN2A	CDKN2A	TLR3	TLR3	G6PD
F2	F2	SLC2A2	SLC2A2	CTSB	CTSB	CD40
SERPINE1	SERPINE1	LPL	LPL	DNM1	DNM1	FN1
ALPL	ALPL	GRM1	GRM1	RIPK1	RIPK1	GJA1
MMP1	MMP1	GJA1	GJA1	HMGB1	HMGB1	PRKAR1A
CDKN2A	CDKN2A	G6PD	G6PD	FAS	FAS	LDHA
SLC2A2	SLC2A2	IL2RA	IL2RA	NFKBIA	NFKBIA	CD36
ELANE	ELANE	LDHA	LDHA	DYRK1A	DYRK1A	NFKBIA
IL2RA	IL2RA	CD36	CD36	RHEB	RHEB	ACTB
GNAS	GNAS	FN1	FN1	NPM1	NPM1	BAX
G6PD	G6PD	ELANE	ELANE	CREB1	CREB1	RIPK1
CD40	CD40	EIF2AK3	EIF2AK3	MERTK	MERTK	TUBB3
FN1	FN1	RIPK1	RIPK1	CD36	CD36	CTSB
GJA1	GJA1	NRAS	NRAS	EPHA4	EPHA4	HK1
CASR	CASR	TGFB2	TGFB2	GAA	GAA	IL6R
PRKAR1A	PRKAR1A	NFKBIA	NFKBIA	HSP90AB1	HSP90AB1	TGFB2
LDHA	LDHA	SHH	SHH	CD40	CD40	KCNH2
CD36	CD36	GSTP1	GSTP1	RPS6KB1	RPS6KB1	DYRK1A
NFKBIA	NFKBIA	BAX	BAX	JUN	JUN	CAT
ENPP1	ENPP1	ACTB	ACTB	TFRC	TFRC	GSTP1
ACTB	ACTB	CASR	CASR	PIKFYVE	PIKFYVE	WNT5A
BAX	BAX	CD40	CD40	DRD2	DRD2	SHH
RIPK1	RIPK1	CAT	CAT	SMARCA4	SMARCA4	TLR3
TUBB3	TUBB3	SLC9A1	SLC9A1	MAPK8	MAPK8	GRIN1
CTSB	CTSB	CD4	CD4	CA2	CA2	CD4
ACVRL1	ACVRL1	BCHE	BCHE	TGFB2	TGFB2	ALDH2
HK1	HK1	TLR3	TLR3	PPP3CA	PPP3CA	HMGB1
IL6R	IL6R	TFRC	TFRC	SRC	SRC	SMARCA4
TGFB2	TGFB2	CTSB	CTSB	RRM1	RRM1	TFRC
KCNH2	KCNH2	ALDH2	ALDH2	NRAS	NRAS	NOS1
DYRK1A	DYRK1A	ZAP70	ZAP70	HK1	HK1	CREB1
CAT	CAT	TUBB3	TUBB3	LDHA	LDHA	MAPK8
GSTP1	GSTP1	HMGB1	HMGB1	BCHE	BCHE	SRC
WNT5A	WNT5A	ITGB2	ITGB2	GATA3	GATA3	GLUD1
SHH	SHH	WNT5A	WNT5A	ADAM17	ADAM17	DRD2
TLR3	TLR3	DNMT3B	DNMT3B	PDGFB	PDGFB	YWHAE
GRIN1	GRIN1	CD19	CD19	HDAC9	HDAC9	NPM1
CD4	CD4	SRC	SRC	GSK3A	GSK3A	GRM1
ALDH2	ALDH2	PRKG1	PRKG1	JAG1	JAG1	JUN
F10	F10	PCNA	PCNA	VIM	VIM	PPP3CA
HMGB1	HMGB1	IL6R	IL6R	WNT5A	WNT5A	GSK3A
SMARCA4	SMARCA4	FTH1	FTH1	ENPP1	ENPP1	CSNK2A1
ZAP70	ZAP70	CA2	CA2	XIAP	XIAP	PCNA
TFRC	TFRC	GRIN1	GRIN1	SCN5A	SCN5A	IL6ST
NOS1	NOS1	MERTK	MERTK	CDKN2A	CDKN2A	CD19
CREB1	CREB1	NOS1	NOS1	SERPINE1	SERPINE1	DNMT3B
MAPK8	MAPK8	DYRK1A	DYRK1A	F2	F2	PRKDC
SRC	SRC	CREB1	CREB1	KAT2A	KAT2A	CHUK
GLUD1	GLUD1	NPM1	NPM1	SMARCA2	SMARCA2	ACVR1
DRD2	DRD2	SMARCA4	SMARCA4	GSTP1	GSTP1	HDAC9
YWHAE	YWHAE	MAPK8	MAPK8	LPL	LPL	CDKN1A
NPM1	NPM1	JUN	JUN	IL6ST	IL6ST	CA2
ITGB2	ITGB2	ACVR1	ACVR1	YWHAE	YWHAE	FTH1
GRM1	GRM1	GLUD1	GLUD1	MMP1	MMP1	CXCR2
JUN	JUN	YWHAE	YWHAE	G6PD	G6PD	VIM
PPP3CA	PPP3CA	PLA2G7	PLA2G7	PCNA	PCNA	MERTK
GSK3A	GSK3A	PRKDC	PRKDC	ATP2A2	ATP2A2	GRIA3
CSNK2A1	CSNK2A1	DPP4	DPP4	RPS6KA1	RPS6KA1	DNM1
PCNA	PCNA	VIM	VIM	ACVR1	ACVR1	PLA2G7
PRKG1	PRKG1	RAC2	RAC2	CAMK2B	CAMK2B	PTPN1
IL6ST	IL6ST	ITGA2B	ITGA2B	MMP14	MMP14	MMP14
CD19	CD19	CDKN1A	CDKN1A	EDNRB	EDNRB	LEPR
DNMT3B	DNMT3B	HDAC9	HDAC9	CXCR2	CXCR2	DPP4
PRKDC	PRKDC	LYN	LYN	CD19	CD19	ACAT1
CHUK	CHUK	IL6ST	IL6ST	ACVRL1	ACVRL1	ITGB1
ACVR1	ACVR1	CHUK	CHUK	IL6R	IL6R	SLC9A1
HDAC9	HDAC9	MMP14	MMP14	SLC9A1	SLC9A1	CAMK2B
CDKN1A	CDKN1A	GRIA3	GRIA3	CSNK2A1	CSNK2A1	CDK2
CA2	CA2	ITGB1	ITGB1	CDKN1A	CDKN1A	KAT5
FTH1	FTH1	GSK3A	GSK3A	CDK2	CDK2	DDR2
CXCR2	CXCR2	DDR2	DDR2	LEPR	LEPR	PLD1
VIM	VIM	CAMK2B	CAMK2B	ITGB1	ITGB1	PLCG1
UGT1A1	UGT1A1	HSP90AB1	HSP90AB1	SLC2A2	SLC2A2	HSD11B1
MERTK	MERTK	LEPR	LEPR	DNMT3B	DNMT3B	SMARCA2
GRIA3	GRIA3	PTPN1	PTPN1	KCNH2	KCNH2	STAT5A
ITGA2B	ITGA2B	DNM1	DNM1	PKM	PKM	LYN
DNM1	DNM1	STAT5A	STAT5A	LYN	LYN	PRKCB
PLA2G7	PLA2G7	PPP3CA	PPP3CA	DPP4	DPP4	RPS6KB1
PTPN1	PTPN1	CXCR2	CXCR2	TOP2A	TOP2A	HSP90AB1
MMP14	MMP14	CSNK2A1	CSNK2A1	ACAT1	ACAT1	PTPN6
LEPR	LEPR	PLD1	PLD1	FTH1	FTH1	PKM
DPP4	DPP4	PKM	PKM	PRKCB	PRKCB	PIKFYVE
RAC2	RAC2	PIKFYVE	PIKFYVE	PTPN1	PTPN1	RRM1
ACAT1	ACAT1	CDK2	CDK2	EPAS1	EPAS1	MAP3K1
ITGB1	ITGB1	EPAS1	EPAS1	KAT5	KAT5	EPHA4
SLC9A1	SLC9A1	ACAT1	ACAT1	PLCG1	PLCG1	HDAC3
CAMK2B	CAMK2B	PLCG1	PLCG1	RARA	RARA	PAK1
CDK2	CDK2	MAP3K1	MAP3K1	HDAC3	HDAC3	RHEB
KAT5	KAT5	RRM1	RRM1	PTPN6	PTPN6	EGLN1
DDR2	DDR2	RPS6KB1	RPS6KB1	UGT1A1	UGT1A1	RPS6KA1
PLD1	PLD1	SMARCA2	SMARCA2	ALDH2	ALDH2	KAT2A
PLCG1	PLCG1	PTPN6	PTPN6	PLA2G7	PLA2G7	APOE
HSD11B1	HSD11B1	KAT5	KAT5	CHUK	CHUK	MAPT
SMARCA2	SMARCA2	HSD11B1	HSD11B1	HSD11B1	HSD11B1	GBA1
STAT5A	STAT5A	ADK	ADK	STAT5A	STAT5A	NOS3
LYN	LYN	NEK2	NEK2	PLD1	PLD1	COL1A1
PRKCB	PRKCB	HDAC3	HDAC3	PAK1	PAK1	LRP5
RARA	RARA	KAT2A	KAT2A	MAP3K1	MAP3K1	MME
RPS6KB1	RPS6KB1	EPHA4	EPHA4	TYMS	TYMS	BDNF

HSP90AB1	HSP90AB1	PRKCB	PRKCB	EGLN1	EGLN1	CP
PTPN6	PTPN6	RHEB	RHEB	MAPT	MAPT	APOA1
ADK	ADK	RPS6KA1	RPS6KA1	APOE	APOE	GDNF
PKM	PKM	EGLN1	EGLN1	CHRNA4	CHRNA4	IL1RN
TYMS	TYMS	PAK1	PAK1	GBA1	GBA1	SERPINA1
PIKFYVE	PIKFYVE	GBA1	GBA1	CP	CP	CASP3
RRM1	RRM1	MAPT	MAPT	NOS3	NOS3	NTRK1
TOP2A	TOP2A	APOE	APOE	SLC1A2	SLC1A2	COL2A1
MAP3K1	MAP3K1	COL1A1	COL1A1	ASAH1	ASAH1	FLNA
EPHA4	EPHA4	LRP5	LRP5	BDNF	BDNF	PCSK9
HDAC3	HDAC3	GDNF	GDNF	GDNF	GDNF	ASAH1
TBXA2R	TBXA2R	NOS3	NOS3	CASP3	CASP3	AGT
PAK1	PAK1	MME	MME	PHGDH	PHGDH	DLD
RHEB	RHEB	CP	CP	COL1A1	COL1A1	ICAM1
EGLN1	EGLN1	DDC	DDC	BCL2L1	BCL2L1	CD40LG
RPS6KA1	RPS6KA1	BDNF	BDNF	ICAM1	ICAM1	SIRT1
KAT2A	KAT2A	APOA1	APOA1	SIRT1	SIRT1	SPR
APOE	APOE	SERPINA1	SERPINA1	CDC42	CDC42	B2M
MAPT	MAPT	SPR	SPR	LRP5	LRP5	HIF1A
GBA1	GBA1	IL1RN	IL1RN	APOA1	APOA1	MMP3
NOS3	NOS3	COL2A1	COL2A1	SCN8A	SCN8A	CDC42
COL1A1	COL1A1	PSMB8	PSMB8	NTRK1	NTRK1	EIF2AK2
LRP5	LRP5	STIM1	STIM1	HSPA8	HSPA8	DSP
MME	MME	ASAH1	ASAH1	MME	MME	CAPN1
BDNF	BDNF	CASP3	CASP3	LAMB1	LAMB1	STIM1
CP	CP	FGA	FGA	MAP3K5	MAP3K5	CPT2
APOA1	APOA1	NTRK1	NTRK1	GAD1	GAD1	HNF4A
GDNF	GDNF	DSP	DSP	HIF1A	HIF1A	RHOA
IL1RN	IL1RN	CAPN1	CAPN1	P4HB	P4HB	KCNQ1
SERPINA1	SERPINA1	HIF1A	HIF1A	DDC	DDC	PIK3CG
CASP3	CASP3	PCSK9	PCSK9	FLNA	FLNA	FGA
NTRK1	NTRK1	AGT	AGT	EIF2AK2	EIF2AK2	SLC1A2
COL2A1	COL2A1	KCNQ1	KCNQ1	GSR	GSR	DDC
FLNA	FLNA	FLNA	FLNA	GRIA1	GRIA1	SERPINC1
PCSK9	PCSK9	SIRT1	SIRT1	B2M	B2M	TRPC6
ASAH1	ASAH1	ICAM1	ICAM1	SERPINA1	SERPINA1	CHRNA4
AGT	AGT	DLD	DLD	RHOA	RHOA	STAT5B
DLD	DLD	CD40LG	CD40LG	IL1RN	IL1RN	PLAT
ICAM1	ICAM1	MMP3	MMP3	PIK3CG	PIK3CG	PRDX1
CD40LG	CD40LG	B2M	B2M	CPT2	CPT2	STK11
SIRT1	SIRT1	HNF4A	HNF4A	LIMK1	LIMK1	GAD1
SPR	SPR	CHRNA4	CHRNA4	STIM1	STIM1	GSR
B2M	B2M	CPT2	CPT2	KAT2B	KAT2B	PLG
HIF1A	HIF1A	CDC42	CDC42	CAPN1	CAPN1	SPARC
MMP3	MMP3	TACR3	TACR3	PIK3C3	PIK3C3	P4HB
CDC42	CDC42	RHOA	RHOA	CD40LG	CD40LG	SCN8A
EIF2AK2	EIF2AK2	PIK3CG	PIK3CG	ITGA4	ITGA4	BLM
DSP	DSP	STK11	STK11	TRPC6	TRPC6	HSPA8
CAPN1	CAPN1	SCN8A	SCN8A	COL2A1	COL2A1	PHGDH
STIM1	STIM1	EIF2AK2	EIF2AK2	SPR	SPR	GGT1
CPT2	CPT2	GLI3	GLI3	DLD	DLD	PNP
GLI3	GLI3	HSPA8	HSPA8	ALDH1A2	ALDH1A2	CYP3A4
HNF4A	HNF4A	PLG	PLG	ABCG2	ABCG2	MAP3K5
RHOA	RHOA	STAT5B	STAT5B	SERPINC1	SERPINC1	BCL2L1
KCNQ1	KCNQ1	CYP3A4	CYP3A4	SPARC	SPARC	ENTPD1
PIK3CG	PIK3CG	TRPC6	TRPC6	TOP1	TOP1	RAD50
FGA	FGA	SLC1A2	SLC1A2	PTK2B	PTK2B	LAMB1
SLC1A2	SLC1A2	GAD1	GAD1	PRDX1	PRDX1	ABCG2
DDC	DDC	SERPINC1	SERPINC1	STK11	STK11	MAP3K7
SERPINC1	SERPINC1	PHGDH	PHGDH	MMP3	MMP3	PIK3C3
TRPC6	TRPC6	MMP13	MMP13	MAP3K7	MAP3K7	CSNK1A1
PSMB8	PSMB8	BCL2L1	BCL2L1	AGT	AGT	KRT18
CHRNA4	CHRNA4	ENTPD1	ENTPD1	DSP	DSP	GRIA1
STAT5B	STAT5B	BLM	BLM	ACVR2B	ACVR2B	PTK2B
PLAT	PLAT	FZD4	FZD4	BUB1B	BUB1B	RARB
MMP13	MMP13	P4HB	P4HB	BLM	BLM	BUB1B
PRDX1	PRDX1	GGT1	GGT1	STAT5B	STAT5B	MGMT
STK11	STK11	GSR	GSR	CACNA1B	CACNA1B	ACACA
AXIN2	AXIN2	PLAT	PLAT	PLAT	PLAT	CACNA1B
GAD1	GAD1	SPARC	SPARC	HNF4A	HNF4A	NRP1
GSR	GSR	PNP	PNP	RAD50	RAD50	ITGA4
PLG	PLG	PRDX1	PRDX1	KCNQ1	KCNQ1	MCL1
SPARC	SPARC	AXIN2	AXIN2	FGA	FGA	ROCK1
P4HB	P4HB	IL2RB	IL2RB	MGMT	MGMT	ALDH1A2
SCN8A	SCN8A	ABCG2	ABCG2	PPP2CA	PPP2CA	SGK1
BLM	BLM	RAD50	RAD50	PCSK9	PCSK9	KAT2B
HSPA8	HSPA8	LAMB1	LAMB1	PGR	PGR	LIMK1
PHGDH	PHGDH	CACNA1B	CACNA1B	PLG	PLG	PIM1
GGT1	GGT1	MAP3K5	MAP3K5	MCL1	MCL1	TOP1
FZD4	FZD4	MAP3K7	MAP3K7	MYB	MYB	SCD
PNP	PNP	MCL1	MCL1	ENTPD1	ENTPD1	PPP2CA
CYP3A4	CYP3A4	RARB	RARB	KRT18	KRT18	PDPK1
MAP3K5	MAP3K5	NRP1	NRP1	ROCK1	ROCK1	ANPEP
BCL2L1	BCL2L1	ACACA	ACACA	PNP	PNP	NPC1
ENTPD1	ENTPD1	KRT18	KRT18	NRP1	NRP1	LRRK2
RAD50	RAD50	SGK1	SGK1	ACACA	ACACA	ATP7B
LAMB1	LAMB1	ADRB1	ADRB1	CYP3A4	CYP3A4	SQSTM1
GRIN2D	GRIN2D	PTK2B	PTK2B	GGT1	GGT1	SMPD1
ABCG2	ABCG2	MAP2K4	MAP2K4	ANPEP	ANPEP	PRNP
MAP3K7	MAP3K7	KAT2B	KAT2B	SGK1	SGK1	VCP
PIK3C3	PIK3C3	GRIA1	GRIA1	PDPK1	PDPK1	GLA
TACR3	TACR3	BUB1B	BUB1B	RARB	RARB	PRKN
CSNK1A1	CSNK1A1	LIMK1	LIMK1	PIM1	PIM1	CYBB
KRT18	KRT18	ITGA4	ITGA4	CSNK1A1	CSNK1A1	ABCA1
GRIA1	GRIA1	PIK3C3	PIK3C3	SCD	SCD	POLG
PTK2B	PTK2B	GRIN2D	GRIN2D	SQSTM1	SQSTM1	MTHFR
RARB	RARB	ROCK1	ROCK1	VCP	VCP	TRPV4
BUB1B	BUB1B	MGMT	MGMT	TBK1	TBK1	PRKAG2
MGMT	MGMT	ACVR2B	ACVR2B	TSC1	TSC1	WNK1
ACACA	ACACA	ALDH1A2	ALDH1A2	PRNP	PRNP	LIPA
MAP2K4	MAP2K4	HCK	HCK	NPC1	NPC1	NLRP3
HCK	HCK	SCD	SCD	VEGFA	VEGFA	PFKM
ADRB1	ADRB1	PIM1	PIM1	POLG	POLG	GYS1
CACNA1B	CACNA1B	ANPEP	ANPEP	HSPD1	HSPD1	TBK1
NRP1	NRP1	CSNK1A1	CSNK1A1	MEF2C	MEF2C	RYR1
ITGA4	ITGA4	PAK3	PAK3	BMP4	BMP4	GPHN
MCL1	MCL1	TOP1	TOP1	CTSF	CTSF	TNFRSF1A
ROCK1	ROCK1	PPP2CA	PPP2CA	TNFRSF1A	TNFRSF1A	RAB7A
PGR	PGR	PDPK1	PDPK1	TBP	TBP	VEGFA
ALDH1A2	ALDH1A2	LRRK2	LRRK2	ATP7B	ATP7B	LRP1
SGK1	SGK1	PRKN	PRKN	KDM1A	KDM1A	ATP2A1
PAK3	PAK3	NPC1	NPC1	FCGR2A	FCGR2A	CHAT
KAT2B	KAT2B	ATP7B	ATP7B	USP8	USP8	CTSF
MYB	MYB	SQSTM1	SQSTM1	MAOA	MAOA	MUC1
LIMK1	LIMK1	SMPD1	SMPD1	LRRK2	LRRK2	APC
PIM1	PIM1	GLA	GLA	ITPR1	ITPR1	TSC1
TOP1	TOP1	VCP	VCP	CAV1	CAV1	GLB1
SCD	SCD	PRNP	PRNP	PRKN	PRKN	VDR
PPP2CA	PPP2CA	POLG	POLG	NLRP3	NLRP3	PGK1
PDPK1	PDPK1	PRKAG2	PRKAG2	SLC1A3	SLC1A3	ALB
ANPEP	ANPEP	ABCA1	ABCA1	CHAT	CHAT	HSPD1
MST1R	MST1R	CYBB	CYBB	TRPV4	TRPV4	C3
NPC1	NPC1	TRPV4	TRPV4	PGK1	PGK1	TBP
LRRK2	LRRK2	MTHFR	MTHFR	GLB1	GLB1	CACNA1G
ATP7B	ATP7B	RYR1	RYR1	CASP1	CASP1	REN
SQSTM1	SQSTM1	TBP	TBP	CASP9	CASP9	NCSTN
SMPD1	SMPD1	RAB7A	RAB7A	RAB7A	RAB7A	ITPR1
PRNP	PRNP	VEGFA	VEGFA	SMPD1	SMPD1	NOS2
VCP	VCP	NLRP3	NLRP3	MTHFR	MTHFR	SNAP25
GLA	GLA	GYS1	GYS1	SNAP25	SNAP25	TNFSF11
PRKN	PRKN	PFKM	PFKM	NOS2	NOS2	SDHB
CYBB	CYBB	LIPA	LIPA	GLA	GLA	ACE2
ABCA1	ABCA1	WNK1	WNK1	KEAP1	KEAP1	ALDOA
POLG	POLG	TBK1	TBK1	HSPA5	HSPA5	MAOA
MTHFR	MTHFR	PGK1	PGK1	STXBP1	STXBP1	ECE1
TRPV4	TRPV4	GLB1	GLB1	SLC1A1	SLC1A1	RUNX1
PRKAG2	PRKAG2	HSPD1	HSPD1	RYR1	RYR1	EDN1
WNK1	WNK1	RRM2B	RRM2B	GPHN	GPHN	NR3C1
LIPA	LIPA	SDHB	SDHB	SPTAN1	SPTAN1	BMP4
NLRP3	NLRP3	TNFRSF1A	TNFRSF1A	LRP1	LRP1	CAV1
PFKM	PFKM	MUC1	MUC1	ABCA1	ABCA1	RRM2B
GYS1	GYS1	ATP1A1	ATP1A1	ALB	ALB	VLDLR
TBK1	TBK1	ITPR1	ITPR1	DDX3X	DDX3X	ERCC2
RYR1	RYR1	LRP1	LRP1	CYBB	CYBB	GUSB
GPHN	GPHN	CTSF	CTSF	ARG1	ARG1	MAPK3
TNFRSF1A	TNFRSF1A	ATP2A1	ATP2A1	TNFSF11	TNFSF11	HSPA5

RAB7A	RAB7A	VDR	VDR	MAPK3	MAPK3	FCGR2A
VEGFA	VEGFA	KCNJ2	KCNJ2	GRIA4	GRIA4	SLC1A3
LRP1	LRP1	REN	REN	DLG4	DLG4	ASS1
ATP2A1	ATP2A1	GPHN	GPHN	UBA1	UBA1	ATP1A1
CHAT	CHAT	APC	APC	HSPA9	HSPA9	LRP2
CTSF	CTSF	BMP4	BMP4	CTSK	CTSK	TUBA1A
MUC1	MUC1	ASS1	ASS1	CACNA1C	CACNA1C	ZEB2
APC	APC	ATP1A2	ATP1A2	VDR	VDR	CACNA1C
TSC1	TSC1	ALB	ALB	EIF4E	EIF4E	CACNA1S
GLB1	GLB1	CACNA1S	CACNA1S	C3	C3	TNNI3
ABCA3	ABCA3	C3	C3	RAC1	RAC1	ATF6
VDR	VDR	NF2	NF2	DICER1	DICER1	ITGAM
PGK1	PGK1	SLC1A3	SLC1A3	GRIK2	GRIK2	SREBF1
ALB	ALB	MRE11	MRE11	CD44	CD44	MSH6
HSPD1	HSPD1	FCGR2A	FCGR2A	EEF2	EEF2	MLH1
C3	C3	TSC1	TSC1	ATF6	ATF6	MYD88
TBP	TBP	CACNA1C	CACNA1C	TUBA1A	TUBA1A	TNFAIP3
CACNA1G	CACNA1G	CACNA1G	CACNA1G	MLH1	MLH1	CTSK
REN	REN	MAOA	MAOA	FOXO3	FOXO3	KCNJ2
NCSTN	NCSTN	CHAT	CHAT	PRKACB	PRKACB	POMC
ITPR1	ITPR1	NOS2	NOS2	PRMT1	PRMT1	STXBP1
NOS2	NOS2	TNFSF11	TNFSF11	PTH1R	PTH1R	TNFRSF1B
SNAP25	SNAP25	TNNI3	TNNI3	MSH6	MSH6	TUBB
TNFSF11	TNFSF11	ALDOA	ALDOA	MYD88	MYD88	GNAQ
SDHB	SDHB	EEF2	EEF2	ITGAM	ITGAM	IGF2
ACE2	ACE2	ACE2	ACE2	MSH2	MSH2	CDH1
ALDOA	ALDOA	GATA4	GATA4	GUSB	GUSB	RAC1
IRAK4	IRAK4	CACNA1H	CACNA1H	IGF2	IGF2	DLG4
MAOA	MAOA	GUSB	GUSB	NQO1	NQO1	PIK3R2
ECE1	ECE1	EDN1	EDN1	KCNJ2	KCNJ2	MSH2
RUNX1	RUNX1	CAV1	CAV1	PIK3CB	PIK3CB	WAS
EDN1	EDN1	MYD88	MYD88	CACNA1H	CACNA1H	ADRB2
NR3C1	NR3C1	USP8	USP8	RRM2B	RRM2B	NQO1
BMP4	BMP4	WAS	WAS	CASP2	CASP2	PTGS1
CAV1	CAV1	HSPA5	HSPA5	MTAP	MTAP	ESR2
RRM2B	RRM2B	CTSK	CTSK	TUBB	TUBB	GATA2
VLDLR	VLDLR	MEF2C	MEF2C	ASS1	ASS1	CD55
ERCC2	ERCC2	MYL2	MYL2	ZEB2	ZEB2	TPO
GATA4	GATA4	ERCC2	ERCC2	NF2	NF2	NF2
GUSB	GUSB	RUNX1	RUNX1	CASK	CASK	FOXO3
MAPK3	MAPK3	NR3C1	NR3C1	SDHB	SDHB	CD44
HSPA5	HSPA5	SREBF1	SREBF1	ATP1A2	ATP1A2	CASP1
FCGR2A	FCGR2A	SNAP25	SNAP25	NCSTN	NCSTN	ATP1A2
SLC1A3	SLC1A3	IGF2	IGF2	CDH1	CDH1	CASP9
ASS1	ASS1	TNFAIP3	TNFAIP3	EDN1	EDN1	YAP1
ATP1A1	ATP1A1	CDH1	CDH1	RUNX1	RUNX1	ALOX5
LRP2	LRP2	ATF6	ATF6	ATP1A1	ATP1A1	SPTAN1
TUBA1A	TUBA1A	MSH6	MSH6	ERCC2	ERCC2	MEF2C
ZEB2	ZEB2	MLH1	MLH1	CPT1A	CPT1A	PCSK1
CACNA1C	CACNA1C	NBN	NBN	VLDLR	VLDLR	ARG1
CACNA1S	CACNA1S	ARG1	ARG1	MAPK9	MAPK9	RB1
TNNI3	TNNI3	HSPA9	HSPA9	LRP2	LRP2	ENO1
ATF6	ATF6	ZEB2	ZEB2	REN	REN	MRE11
ITGAM	ITGAM	ITGAM	ITGAM	CSNK1D	CSNK1D	KEAP1
SREBF1	SREBF1	GATA2	GATA2	MUC1	MUC1	UBA1
MSH6	MSH6	MSH2	MSH2	LIPA	LIPA	MYL2
MLH1	MLH1	DICER1	DICER1	CACNA1S	CACNA1S	NCAM1
MYD88	MYD88	UBA1	UBA1	CASP6	CASP6	CDKN1B
TNFAIP3	TNFAIP3	ADRB2	ADRB2	TNFAIP3	TNFAIP3	SLC2A3
CTSK	CTSK	TUBA1A	TUBA1A	CACNA1G	CACNA1G	CPT1A
KCNJ2	KCNJ2	POMC	POMC	PI4KA	PI4KA	EEF2
POMC	POMC	SPTAN1	SPTAN1	ALDOA	ALDOA	DICER1
GCK	GCK	NQO1	NQO1	IFNAR2	IFNAR2	PTK2
STXBP1	STXBP1	MAPK3	MAPK3	ACTG1	ACTG1	HSPA9
TNFRSF1B	TNFRSF1B	CYP17A1	CYP17A1	GYS1	GYS1	SLC1A1
TUBB	TUBB	STXBP1	STXBP1	NCAM1	NCAM1	ACTG1
GNAQ	GNAQ	NCSTN	NCSTN	GCK	GCK	PLCB1
IGF2	IGF2	CASP9	CASP9	NR3C1	NR3C1	BRIP1
F12	F12	KEAP1	KEAP1	YAP1	YAP1	USP8
CDH1	CDH1	TNFRSF1B	TNFRSF1B	FOLR1	FOLR1	FURIN
RAC1	RAC1	CD55	CD55	IFNAR1	IFNAR1	KDM1A
DLG4	DLG4	KDM1A	KDM1A	SLC12A2	SLC12A2	VKORC1
PIK3R2	PIK3R2	ECE1	ECE1	POMC	POMC	PIK3CB
MSH2	MSH2	CASP1	CASP1	GABRG2	GABRG2	CASP7
WAS	WAS	RB1	RB1	WNK1	WNK1	TAP1
ADRB2	ADRB2	SLC40A1	SLC40A1	CDKN1B	CDKN1B	BLK
CYP17A1	CYP17A1	CD44	CD44	GABBR1	GABBR1	STAT2
NQO1	NQO1	PTK2	PTK2	EDNRA	EDNRA	CASK
TSHR	TSHR	ESR2	ESR2	CD28	CD28	CASP2
PTGS1	PTGS1	VLDLR	VLDLR	FASN	FASN	GLS
ESR2	ESR2	BRIP1	BRIP1	USP7	USP7	FANCA
GATA2	GATA2	PCSK1	PCSK1	ECE1	ECE1	CACNA1H
PDE4D	PDE4D	DLG4	DLG4	GNAQ	GNAQ	PRKACB
CD55	CD55	LRP2	LRP2	PLCB1	PLCB1	CD28
TPO	TPO	FOXO3	FOXO3	SREBF1	SREBF1	GABRG2
NF2	NF2	PRKACB	PRKACB	PRKAG2	PRKAG2	FOLR1
FOXO3	FOXO3	PMS2	PMS2	GLS	GLS	EIF4E
CD44	CD44	TPO	TPO	TNFRSF1B	TNFRSF1B	CASP6
CASP1	CASP1	ENO1	ENO1	EPCAM	EPCAM	CHRM3
ATP1A2	ATP1A2	CDKN1B	CDKN1B	ADRB2	ADRB2	FBP1
CASP9	CASP9	TRPV1	TRPV1	FBP1	FBP1	PLA2G4A
YAP1	YAP1	GNAQ	GNAQ	APC	APC	PRKCQ
ALOX5	ALOX5	PDE4D	PDE4D	ENO1	ENO1	IRAK1
F7	F7	RAC1	RAC1	CD55	CD55	SLC12A2
SPTAN1	SPTAN1	PTGS1	PTGS1	PIK3R2	PIK3R2	YWHAZ
MEF2C	MEF2C	NCAM1	NCAM1	YWHAZ	YWHAZ	DDX3X
PCSK1	PCSK1	TUBB	TUBB	TSHR	TSHR	CTH
CDC25A	CDC25A	YAP1	YAP1	ACE2	ACE2	FASN
ARG1	ARG1	TAP1	TAP1	CAD	CAD	TRPV1
RB1	RB1	CHRM3	CHRM3	MRE11	MRE11	MAPK9
ENO1	ENO1	BLK	BLK	GATA2	GATA2	CAD
EPCAM	EPCAM	SLC2A3	SLC2A3	PFKM	PFKM	OPRM1
MRE11	MRE11	ALOX5	ALOX5	TNNI3	TNNI3	GABBR1
KEAP1	KEAP1	PIK3R2	PIK3R2	WAS	WAS	CAMK2G
BMP1	BMP1	FANCA	FANCA	RB1	RB1	PI4KA
UBA1	UBA1	STAT2	STAT2	BUB1	BUB1	USP7
PTH1R	PTH1R	CDC25A	CDC25A	ATP2A1	ATP2A1	GRIK2
MYL2	MYL2	CPT1A	CPT1A	TRPV1	TRPV1	ZEB1
NCAM1	NCAM1	CCNE1	CCNE1	MYL2	MYL2	NFATC1
EDNRA	EDNRA	SLC1A1	SLC1A1	BLK	BLK	GRIA4
CDKN1B	CDKN1B	IRAK1	IRAK1	BRIP1	BRIP1	CSNK1D
SLC2A3	SLC2A3	CASK	CASK	TNFRSF10B	TNFRSF10B	DAPK1
CPT1A	CPT1A	FOLR1	FOLR1	NBN	NBN	ADAM9
EEF2	EEF2	ACTG1	ACTG1	IRAK1	IRAK1	CDC25C
DICER1	DICER1	PIK3CB	PIK3CB	PLA2G4A	PLA2G4A	FKBP5
PTK2	PTK2	CD28	CD28	SCNN1A	SCNN1A	PRKCE
HSPA9	HSPA9	CASP7	CASP7	PTK2	PTK2	RXRA
PMS2	PMS2	VKORC1	VKORC1	CAMK2G	CAMK2G	BUB1
SLC1A1	SLC1A1	SLC12A2	SLC12A2	RDX	RDX	ATP1B1
ACTG1	ACTG1	FURIN	FURIN	CD247	CD247	IFNAR1
PLCB1	PLCB1	CASP2	CASP2	PRKAA2	PRKAA2	RDX
BRIP1	BRIP1	CASP6	CASP6	ZEB1	ZEB1	FAAH
USP8	USP8	OPRM1	OPRM1	FANCA	FANCA	SLC29A1
FURIN	FURIN	GABRG2	GABRG2	ABCC1	ABCC1	CES1
KDM1A	KDM1A	USP7	USP7	PTGS1	PTGS1	ACTN1
VKORC1	VKORC1	PRKD1	PRKD1	LEF1	LEF1	REL
PIK3CB	PIK3CB	GABBR1	GABBR1	FAAH	FAAH	ABCC1
CASP7	CASP7	GRK2	GRK2	PTPRF	PTPRF	TNFRSF10B
TAP1	TAP1	REL	REL	STAT2	STAT2	PTGER2
BLK	BLK	GRIK2	GRIK2	CASP7	CASP7	ITGA6
CYP11A1	CYP11A1	FBP1	FBP1	FURIN	FURIN	ITGAV
GRK2	GRK2	YWHAZ	YWHAZ	PTGER2	PTGER2	PRMT1
STAT2	STAT2	DDX3X	DDX3X	SLC2A3	SLC2A3	PRKCZ
CASK	CASK	FKBP5	FKBP5	OPRM1	OPRM1	FKBP1A
SLC40A1	SLC40A1	PRKCQ	PRKCQ	DAPK1	DAPK1	NR1D1
CASP2	CASP2	PLA2G4A	PLA2G4A	F7	F7	LEF1
GLS	GLS	FASN	FASN	TAP1	TAP1	PPP1CA
RORA	RORA	RORA	RORA	PPP1CA	PPP1CA	TYRO3
FANCA	FANCA	EIF4E	EIF4E	ACTN1	ACTN1	PRKAA2
CACNA1H	CACNA1H	PLCB1	PLCB1	REL	REL	CAMK2D
PRKACB	PRKACB	GRIA4	GRIA4	FST	FST	MAP2K3
PLCB3	PLCB3	MAPK9	MAPK9	CCNE1	CCNE1	ITGA5
CD28	CD28	CAD	CAD	PCSK1	PCSK1	BIRC2
GABRG2	GABRG2	TPH2	TPH2	NR1D1	NR1D1	PAK4
FOLR1	FOLR1	CTH	CTH	ESR2	ESR2	PKN1
EIF4E	EIF4E	ZEB1	ZEB1	TYRO3	TYRO3	PTPRF

CD247	CD247	TNC	TNC	BIRC2	BIRC2	NOD2
CASP6	CASP6	GLS	GLS	TPO	TPO	VWF
CHRM3	CHRM3	DAPK1	DAPK1	FKBP1A	FKBP1A	MFN2
FBP1	FBP1	CAMK2G	CAMK2G	ATP1B1	ATP1B1	PSAP
PLA2G4A	PLA2G4A	IFNAR1	IFNAR1	ABCA3	ABCA3	PARK7
PRKCQ	PRKCQ	ADAM9	ADAM9	ALOX5	ALOX5	GFAP
IRAK1	IRAK1	ETS1	ETS1	WEE1	WEE1	HEXB
PRKD1	PRKD1	PLCB3	PLCB3	FKBP5	FKBP5	GRN
SLC12A2	SLC12A2	PI4KA	PI4KA	VKORC1	VKORC1	PLA2G6
YWHAZ	YWHAZ	NR1D1	NR1D1	CHRM3	CHRM3	GJB1
DDX3X	DDX3X	NFATC1	NFATC1	CAMK2D	CAMK2D	SCN9A
CTH	CTH	BUB1	BUB1	NFATC1	NFATC1	ENG
EPOR	EPOR	RXRA	RXRA	ADAM9	ADAM9	CTLA4
FASN	FASN	FKBP1A	FKBP1A	FECH	FECH	TTR
TRPV1	TRPV1	ACTN1	ACTN1	ITGAV	ITGAV	IFIH1
MAPK9	MAPK9	TNFRSF10B	TNFRSF10B	ITGA5	ITGA5	INS
CAD	CAD	FAAH	FAAH	PRKCZ	PRKCZ	VHL
OPRM1	OPRM1	SLC29A1	SLC29A1	F12	F12	IDS
GABBR1	GABBR1	MAPK7	MAPK7	PRKCE	PRKCE	ACHE
IFNAR2	IFNAR2	RDX	RDX	SLC29A1	SLC29A1	ITCH
CAMK2G	CAMK2G	ABCC1	ABCC1	CTH	CTH	TUBA4A
PI4KA	PI4KA	PRKAA2	PRKAA2	ITGA6	ITGA6	CLU
TNC	TNC	ITGA6	ITGA6	CDC25C	CDC25C	SGSH
USP7	USP7	PRMT1	PRMT1	HDAC5	HDAC5	CCL2
GRIK2	GRIK2	ATP1B1	ATP1B1	PAK4	PAK4	JUP
ZEB1	ZEB1	CSNK1D	CSNK1D	PKN1	PKN1	IGF1
NFATC1	NFATC1	ITGAV	ITGAV	MAP2K3	MAP2K3	FBN1
FECH	FECH	LEF1	LEF1	CES1	CES1	CTSA
CHRM2	CHRM2	MAP2K3	MAP2K3	PRKCQ	PRKCQ	ADAR
GRIA4	GRIA4	PTGER2	PTGER2	GRN	GRN	GCH1
TLR8	TLR8	CHRM2	CHRM2	TUBA4A	TUBA4A	FOXP3
CSNK1D	CSNK1D	PRKCE	PRKCE	HNRNPA1	HNRNPA1	ATP1A3
DAPK1	DAPK1	CDC25C	CDC25C	CYLD	CYLD	DNM1L
ETS1	ETS1	PRKCZ	PRKCZ	PARK7	PARK7	PRF1
ADAM9	ADAM9	TYRO3	TYRO3	CTLA4	CTLA4	ACTA2
CDC25C	CDC25C	PPP1CA	PPP1CA	VRK1	VRK1	NF1
FKBP5	FKBP5	PAK4	PAK4	PPARGC1A	PPARGC1A	UBE3A
PRKCE	PRKCE	CES1	CES1	TTR	TTR	CETP
RXRA	RXRA	BIRC2	BIRC2	UBE3A	UBE3A	PCCA
BUB1	BUB1	ITGA5	ITGA5	PRF1	PRF1	TTN
ATP1B1	ATP1B1	CAMK2D	CAMK2D	ADAMTS13	ADAMTS13	CYLD
IFNAR1	IFNAR1	PKN1	PKN1	FCGR2B	FCGR2B	PROM1
RDX	RDX	PARK7	PARK7	GFAP	GFAP	SLC6A4
FAAH	FAAH	MFN2	MFN2	HEXB	HEXB	PRKAR1B
MTAP	MTAP	VWF	VWF	PRKAR1B	PRKAR1B	HSPG2
SLC29A1	SLC29A1	NOD2	NOD2	CACNA1A	CACNA1A	FASLG
CES1	CES1	PLA2G6	PLA2G6	MFN2	MFN2	BRCA2
ACTN1	ACTN1	HEXB	HEXB	IGF1	IGF1	ENO3
REL	REL	PKD2	PKD2	CCL2	CCL2	HNRNPA1
ABCC1	ABCC1	PSAP	PSAP	CYP27B1	CYP27B1	RUNX2
TNFRSF10B	TNFRSF10B	GJB1	GJB1	PSAP	PSAP	PDCD1
PTGER2	PTGER2	GFAP	GFAP	YWHAQ	YWHAQ	CACNA1A
TPH2	TPH2	GRN	GRN	PLA2G6	PLA2G6	SDHA
ITGA6	ITGA6	TTR	TTR	DNM1L	DNM1L	MYH7
ITGAV	ITGAV	SCN9A	SCN9A	INS	INS	MVK
PRMT1	PRMT1	GCH1	GCH1	ADAR	ADAR	MYH9
WEE1	WEE1	CTLA4	CTLA4	PAX6	PAX6	IDE
PRKCZ	PRKCZ	ENG	ENG	HPRT1	HPRT1	SLC25A4
FKBP1A	FKBP1A	NCF2	NCF2	GLUL	GLUL	FLNB
NR1D1	NR1D1	ATP1A3	ATP1A3	APAF1	APAF1	IKBKG
LIPE	LIPE	TTN	TTN	CAPN3	CAPN3	SOD2
LEF1	LEF1	TAF1	TAF1	PDCD1	PDCD1	L1CAM
PPP1CA	PPP1CA	SLC18A2	SLC18A2	DES	DES	HMGCR
TYRO3	TYRO3	ACTA2	ACTA2	FGF8	FGF8	ADAMTS13
HDAC5	HDAC5	DNM1L	DNM1L	TTN	TTN	NRG1
PRKAA2	PRKAA2	VHL	VHL	SLC6A4	SLC6A4	PPARGC1A
CAMK2D	CAMK2D	MYH7	MYH7	NRXN1	NRXN1	KL
MAP2K3	MAP2K3	PRF1	PRF1	NRG1	NRG1	HPRT1
ITGA5	ITGA5	JUP	JUP	FOXP3	FOXP3	ACTA1
FST	FST	CACNA1A	CACNA1A	SCN1A	SCN1A	DES
MAPK7	MAPK7	ITCH	ITCH	L1CAM	L1CAM	MIF
BIRC2	BIRC2	HNRNPA1	HNRNPA1	SOD2	SOD2	TNNT2
PAK4	PAK4	SDHA	SDHA	MUSK	MUSK	RYR2
NCOA3	NCOA3	F8	F8	IKBKG	IKBKG	LPAR1
PKN1	PKN1	FBN1	FBN1	GSN	GSN	FUCA1
PTPRF	PTPRF	DES	DES	KLF4	KLF4	RAB27A
NOD2	NOD2	INS	INS	SLC18A2	SLC18A2	GALNS
VWF	VWF	COL3A1	COL3A1	WT1	WT1	SCN1A
MFN2	MFN2	NCF4	NCF4	KCNQ2	KCNQ2	TYR
PSAP	PSAP	FOXP3	FOXP3	IFIH1	IFIH1	TYMP
PARK7	PARK7	CCL2	CCL2	ENO2	ENO2	PAX6
GFAP	GFAP	SLC12A3	SLC12A3	PC	PC	CYP2D6
HEXB	HEXB	F9	F9	CLU	CLU	SLC18A2
GRN	GRN	CTSA	CTSA	LOX	LOX	GSN
PLA2G6	PLA2G6	TUBA4A	TUBA4A	NF1	NF1	IRS1
GJB1	GJB1	IGF1	IGF1	GRM5	GRM5	HTR2A
SCN9A	SCN9A	IFIH1	IFIH1	NGFR	NGFR	CYP27B1
ENG	ENG	TYMP	TYMP	COL1A2	COL1A2	VRK1
COL1A2	COL1A2	PPARGC1A	PPARGC1A	FGF10	FGF10	CYP2C9
CTLA4	CTLA4	SLC4A1	SLC4A1	RYR2	RYR2	ATF4
NCF2	NCF2	VRK1	VRK1	P2RX7	P2RX7	GLUL
TTR	TTR	NF1	NF1	RIGI	RIGI	LOX
IFIH1	IFIH1	PROM1	PROM1	SMC1A	SMC1A	NMNAT1
INS	INS	CAPN3	CAPN3	ADORA2A	ADORA2A	C1S
VHL	VHL	P2RY12	P2RY12	SPTLC2	SPTLC2	SPTLC2
IDS	IDS	ACHE	ACHE	CD8A	CD8A	PDHA1
ACHE	ACHE	FASLG	FASLG	CDK1	CDK1	CYP19A1
ITCH	ITCH	PDHA1	PDHA1	IRS1	IRS1	SLC19A1
TUBA4A	TUBA4A	ADCY5	ADCY5	IDS	IDS	ENO2
CLU	CLU	RYR2	RYR2	FBN1	FBN1	FCGR3A
SGSH	SGSH	RUNX2	RUNX2	TBXAS1	TBXAS1	PGM1
F8	F8	IKBKG	IKBKG	MYH7	MYH7	SCARB1
CCL2	CCL2	ATF4	ATF4	GJB1	GJB1	LIG4
JUP	JUP	ENO3	ENO3	TYMP	TYMP	AHR
IGF1	IGF1	PRKAR1B	PRKAR1B	ACHE	ACHE	KIF11
FBN1	FBN1	BRCA2	BRCA2	SIRT3	SIRT3	RIGI
CTSA	CTSA	IDS	IDS	ATP1A3	ATP1A3	F13A1
ADAR	ADAR	SGSH	SGSH	XDH	XDH	PIN1
GCH1	GCH1	ADAMTS13	ADAMTS13	HTR2A	HTR2A	P2RY12
NCF4	NCF4	TNNT2	TNNT2	P2RY12	P2RY12	CAPN3
FOXP3	FOXP3	MVK	MVK	NPHS1	NPHS1	CD8A
ATP1A3	ATP1A3	SLC6A4	SLC6A4	SIRT2	SIRT2	CTNNA1
DNM1L	DNM1L	PDCD1	PDCD1	CTSA	CTSA	PC
PRF1	PRF1	SCN2A	SCN2A	FASLG	FASLG	IL2RG
ACTA2	ACTA2	CLU	CLU	FUCA1	FUCA1	CD46
NF1	NF1	TBXAS1	TBXAS1	CTSL	CTSL	FYN
UBE3A	UBE3A	SLC25A4	SLC25A4	CUL3	CUL3	HSPA1A
CETP	CETP	GLDC	GLDC	SMC3	SMC3	PDP1
PCCA	PCCA	MYH9	MYH9	ERN1	ERN1	FGF8
TTN	TTN	ADORA1	ADORA1	GCH1	GCH1	GNAO1
CYLD	CYLD	HSPG2	HSPG2	ENG	ENG	C1R
PROM1	PROM1	SOD2	SOD2	KMT2A	KMT2A	SCN2A
SLC12A1	SLC12A1	L1CAM	L1CAM	HSP90B1	HSP90B1	P2RX7
SLC6A4	SLC6A4	ADAR	ADAR	SGSH	SGSH	WNT3
PRKAR1B	PRKAR1B	TNK2	TNK2	SLC25A4	SLC25A4	FCGR2B
HSPG2	HSPG2	CETP	CETP	DMPK	DMPK	PPIB
FASLG	FASLG	UBE3A	UBE3A	KIF5B	KIF5B	DHFR
BRCA2	BRCA2	PAX6	PAX6	SCN9A	SCN9A	MITF
ENO3	ENO3	HPRT1	HPRT1	FGF1	FGF1	NGFR
HNRNPA1	HNRNPA1	CYP2D6	CYP2D6	RASA1	RASA1	KCNMA1
RUNX2	RUNX2	TYR	TYR	RRM2	RRM2	SMC1A
PDCD1	PDCD1	RAB27A	RAB27A	ATF4	ATF4	IL1R1
CACNA1A	CACNA1A	GALNS	GALNS	SDHA	SDHA	TAF1
SDHA	SDHA	SCN1A	SCN1A	TFAP2A	TFAP2A	WNT7A
MYH7	MYH7	ATP2B2	ATP2B2	EIF4A1	EIF4A1	CYP1A1
MVK	MVK	FUCA1	FUCA1	SCN2A	SCN2A	FGF10
WT1	WT1	TPM1	TPM1	C1QA	C1QA	TBXAS1
MYH9	MYH9	KL	KL	PDHA1	PDHA1	VCL
IDE	IDE	FCGR2B	FCGR2B	XPO1	XPO1	STX1A
SLC25A4	SLC25A4	KCNJ5	KCNJ5	BRCA2	BRCA2	BMP7
FLNB	FLNB	CD8A	CD8A	IDE	IDE	TGM2
IKBKG	IKBKG	HMGCR	HMGCR	LIG4	LIG4	GPI
SOD2	SOD2	PDE2A	PDE2A	KDM6A	KDM6A	TLR7
L1CAM	L1CAM	ACTA1	ACTA1	TGM2	TGM2	KIF5B
HMGCR	HMGCR	NRG1	NRG1	SLC11A2	SLC11A2	CSNK2B
ADAMTS13	ADAMTS13	ABCC6	ABCC6	MYH9	MYH9	OXTR

SLC12A3	SLC12A3	NMNAT1	NMNAT1	PROM1	PROM1	KLF4
NRG1	NRG1	FCGR3A	FCGR3A	PPM1D	PPM1D	RASA1
PPARGC1A	PPARGC1A	FLNB	FLNB	KCNMA1	KCNMA1	CYP1B1
KCNJ1	KCNJ1	GNAO1	GNAO1	PIN1	PIN1	TPM1
KL	KL	CYP27B1	CYP27B1	ERCC3	ERCC3	ALDH7A1
HPRT1	HPRT1	FGF8	FGF8	SCARB1	SCARB1	NRXN1
ACTA1	ACTA1	RIGI	RIGI	HSPG2	HSPG2	ERN1
DES	DES	HTR2A	HTR2A	GABRA1	GABRA1	ADORA2A
MIF	MIF	CFI	CFI	ACTA2	ACTA2	TAB2
TNNT2	TNNT2	NKX2-1	NKX2-1	PGM1	PGM1	GLDC
RYR2	RYR2	FGF10	FGF10	DHCR7	DHCR7	XDH
LPAR1	LPAR1	MIF	MIF	MVK	MVK	CUL3
FUCA1	FUCA1	PC	PC	CTSC	CTSC	SMC3
RAB27A	RAB27A	LOX	LOX	ALDH7A1	ALDH7A1	DCN
GALNS	GALNS	GATA6	GATA6	CAPN2	CAPN2	OAT
SCN1A	SCN1A	AHR	AHR	FYN	FYN	TCF7L2
TYR	TYR	CYP19A1	CYP19A1	RUNX2	RUNX2	AXIN1
TYMP	TYMP	VCL	VCL	NMNAT1	NMNAT1	BMPR1B
PAX6	PAX6	IL2RG	IL2RG	HDAC8	HDAC8	GNA11
CYP2D6	CYP2D6	KLF4	KLF4	GALNS	GALNS	CDK1
SLC18A2	SLC18A2	CYP2C9	CYP2C9	FLNB	FLNB	MECOM
GSN	GSN	PCCA	PCCA	GLDC	GLDC	WNT3A
NPHS1	NPHS1	RASA1	RASA1	ACTA1	ACTA1	SP1
IRS1	IRS1	BMP7	BMP7	VHL	VHL	DMPK
HTR2A	HTR2A	ADORA2A	ADORA2A	CAMK4	CAMK4	CCND2
GATA6	GATA6	IL1R1	IL1R1	SYNGAP1	SYNGAP1	YWHAQ
ABCC6	ABCC6	TLR7	TLR7	TAF1	TAF1	SLC11A2
CYP27B1	CYP27B1	SPTLC2	SPTLC2	EPHA1	EPHA1	BRD4
VRK1	VRK1	CYP1A1	CYP1A1	PCCA	PCCA	KCNQ2
CYP2C9	CYP2C9	MITF	MITF	AXIN1	AXIN1	CTSL
ATF4	ATF4	GSN	GSN	STX1A	STX1A	GPX4
KMT2A	KMT2A	LIG4	LIG4	COL3A1	COL3A1	ABCC2
SLC4A1	SLC4A1	IRS1	IRS1	YY1	YY1	CD38
GLUL	GLUL	WNT3	WNT3	MITF	MITF	FOLH1
LOX	LOX	XDH	XDH	SLC12A5	SLC12A5	GRB2
NMNAT1	NMNAT1	MSN	MSN	ATP2B2	ATP2B2	GLI1
C1S	C1S	CYLD	CYLD	USP9X	USP9X	KCNJ5
SPTLC2	SPTLC2	ALDH7A1	ALDH7A1	VCL	VCL	EXT1
PDHA1	PDHA1	GLI1	GLI1	SLC16A1	SLC16A1	MUSK
CYP19A1	CYP19A1	CUL3	CUL3	SCN3A	SCN3A	ARAF
SLC19A1	SLC19A1	PGM1	PGM1	PKD2	PKD2	ERCC3
ENO2	ENO2	ENO2	ENO2	MIF	MIF	CTSC
FCGR3A	FCGR3A	HSPA1A	HSPA1A	TNNT2	TNNT2	ALDH5A1
PGM1	PGM1	CYP1B1	CYP1B1	OAT	OAT	DHCR7
SCARB1	SCARB1	CD46	CD46	ANGPT2	ANGPT2	PIK3C2A
LIG4	LIG4	PPIB	PPIB	GNAO1	GNAO1	YY1
AHR	AHR	SLC19A1	SLC19A1	VWF	VWF	POR
KIF11	KIF11	SLC11A2	SLC11A2	CPS1	CPS1	PROS1
RIGI	RIGI	GPX4	GPX4	IL2RG	IL2RG	CAPN2
F13A1	F13A1	NR1H4	NR1H4	EXT1	EXT1	APAF1
PIN1	PIN1	GFRA1	GFRA1	ALDH5A1	ALDH5A1	KITLG
P2RY12	P2RY12	TGM2	TGM2	YWHAG	YWHAG	KNG1
CAPN3	CAPN3	CTNNA1	CTNNA1	RPL5	RPL5	CTBP1
CD8A	CD8A	TUBG1	TUBG1	AHR	AHR	KLK3
CTNNA1	CTNNA1	OAT	OAT	HMGCR	HMGCR	ANXA2
NKX2-1	NKX2-1	TAB2	TAB2	GLI1	GLI1	TMPRSS2
PC	PC	GLUL	GLUL	HSPA1A	HSPA1A	FGF1
IL2RG	IL2RG	IMPDH1	IMPDH1	MDH2	MDH2	CYP2C8
CD46	CD46	DHFR	DHFR	RAB27A	RAB27A	FGG
FYN	FYN	IDE	IDE	KCNJ5	KCNJ5	RPL5
HSPA1A	HSPA1A	MECOM	MECOM	PPIB	PPIB	GNAI1
CPS1	CPS1	FYN	FYN	GABRB3	GABRB3	GFRA1
PDP1	PDP1	SCARB1	SCARB1	CYP2C9	CYP2C9	ANXA1
PCK1	PCK1	C1S	C1S	POLE	POLE	SLC6A2
FGF8	FGF8	AVPR2	AVPR2	EIF4EBP1	EIF4EBP1	SIRT3
GNAO1	GNAO1	SMC1A	SMC1A	GPX4	GPX4	MSN
C1R	C1R	TMPRSS2	TMPRSS2	BMP7	BMP7	SLC16A1
SCN2A	SCN2A	P2RX7	P2RX7	ITGA3	ITGA3	INPPL1
AQP2	AQP2	CYP24A1	CYP24A1	INPPL1	INPPL1	YWHAG
P2RX7	P2RX7	NRXN1	NRXN1	PDP1	PDP1	AHCY
WNT3	WNT3	BMPR1B	BMPR1B	TLR7	TLR7	SYNGAP1
FCGR2B	FCGR2B	YY1	YY1	MAP2K6	MAP2K6	TUBG1
AVPR2	AVPR2	GNA11	GNA11	TUBG1	TUBG1	TFAP2A
PPIB	PPIB	SP1	SP1	KL	KL	PDE2A
DHFR	DHFR	CTSC	CTSC	CTNNA1	CTNNA1	MDH2
MITF	MITF	SIRT3	SIRT3	SORD	SORD	USP9X
NGFR	NGFR	MUSK	MUSK	TMPRSS2	TMPRSS2	RPL11
KCNMA1	KCNMA1	KIF11	KIF11	DHFR	DHFR	MAT2A
SMC1A	SMC1A	AQP1	AQP1	GRB2	GRB2	C1QA
IL1R1	IL1R1	F13A1	F13A1	IL1R1	IL1R1	SLC12A5
TAF1	TAF1	CD38	CD38	CCND2	CCND2	TNK2
WNT7A	WNT7A	KITLG	KITLG	BMPR1B	BMPR1B	ANGPT1
GLI2	GLI2	KNG1	KNG1	TYR	TYR	CASP4
ADCY5	ADCY5	FANCC	FANCC	DUSP6	DUSP6	PAX5
CYP1A1	CYP1A1	OXTR	OXTR	BRD4	BRD4	ATP2B2
FGF10	FGF10	EXT1	EXT1	KIF11	KIF11	CCNB1
TBXAS1	TBXAS1	KCNQ2	KCNQ2	MMP7	MMP7	GABRB3
VCL	VCL	STX1A	STX1A	ATP6V1B2	ATP6V1B2	CACNA1E
STX1A	STX1A	NGFR	NGFR	FOLH1	FOLH1	MMP7
PLA2G2A	PLA2G2A	KCNMA1	KCNMA1	WRN	WRN	ATP6V1B2
BMP7	BMP7	YWHAQ	YWHAQ	CD79A	CD79A	STK4
TGM2	TGM2	C1QA	C1QA	KITLG	KITLG	SIRT2
GPI	GPI	PLA2G2A	PLA2G2A	YWHAB	YWHAB	SLC2A4
TLR7	TLR7	APAF1	APAF1	NOD2	NOD2	SORD
KIF5B	KIF5B	SLC6A2	SLC6A2	SLC6A9	SLC6A9	CSNK1E
CSNK2B	CSNK2B	DMPK	DMPK	GNA11	GNA11	GALK1
OXTR	OXTR	PIK3C2A	PIK3C2A	CFI	CFI	GRM5
KLF4	KLF4	ADRA2A	ADRA2A	C1R	C1R	XPO1
RASA1	RASA1	WNT3A	WNT3A	CSNK2B	CSNK2B	EIF4EBP1
CYP1B1	CYP1B1	TCF7L2	TCF7L2	C1S	C1S	GABRA1
TPM1	TPM1	ERN1	ERN1	ANGPT1	ANGPT1	RRAS2
ALDH7A1	ALDH7A1	DLL4	DLL4	ENO3	ENO3	NR2F2
NRXN1	NRXN1	ANGPT1	ANGPT1	SLC2A4	SLC2A4	MAPK12
MPL	MPL	AXIN1	AXIN1	DCN	DCN	SLC6A9
ERN1	ERN1	ABCC2	ABCC2	TCF7L2	TCF7L2	FOXA2
ADORA2A	ADORA2A	TLR1	TLR1	CETP	CETP	ATF2
TAB2	TAB2	C1R	C1R	CTBP1	CTBP1	CPE
GLDC	GLDC	PROS1	PROS1	WNT3A	WNT3A	SCN3A
AQP1	AQP1	CTSL	CTSL	CACNA1E	CACNA1E	ODC1
XDH	XDH	WNT7A	WNT7A	ANXA2	ANXA2	HSP90B1
CUL3	CUL3	GPI	GPI	MECOM	MECOM	NAMPT
SMC3	SMC3	SMC3	SMC3	CASP4	CASP4	PBX1
DCN	DCN	ERCC3	ERCC3	PAX5	PAX5	MAP2K6
OAT	OAT	SIRT2	SIRT2	MAT2A	MAT2A	CD22
TCF7L2	TCF7L2	POLE	POLE	CD38	CD38	ITGB6
CYP24A1	CYP24A1	POR	POR	TNK2	TNK2	NME1
AXIN1	AXIN1	CACNA1E	CACNA1E	FOXA2	FOXA2	EPHA3
KDM6A	KDM6A	RPL5	RPL5	CYP2D6	CYP2D6	AKR1B1
BMPR1B	BMPR1B	DHCR7	DHCR7	ITCH	ITCH	ESRRB
GNA11	GNA11	GRM5	GRM5	RPL11	RPL11	YWHAB
CDK1	CDK1	KYNU	KYNU	ABCC2	ABCC2	NEDD4
MECOM	MECOM	BRD4	BRD4	JUP	JUP	EEF2K
WNT3A	WNT3A	FGG	FGG	TPM1	TPM1	CA9
HDAC8	HDAC8	CDK1	CDK1	GNAI1	GNAI1	GOT2
SP1	SP1	YWHAG	YWHAG	CASP10	CASP10	ALDH1A1
ADRA2A	ADRA2A	CTBP1	CTBP1	TAB2	TAB2	IMPDH2
DMPK	DMPK	MC1R	MC1R	AHCY	AHCY	ADCY3
CCND2	CCND2	FOXA2	FOXA2	GPI	GPI	EIF4A1
YWHAQ	YWHAQ	CCND2	CCND2	CYP1B1	CYP1B1	EPHA1
SLC11A2	SLC11A2	DCN	DCN	RPA1	RPA1	CAMK4
BRD4	BRD4	CYP2C8	CYP2C8	RRAS2	RRAS2	CAMKK2
KCNQ2	KCNQ2	PLK4	PLK4	ODC1	ODC1	CDH5
CTSL	CTSL	ALDH5A1	ALDH5A1	EPHA3	EPHA3	SPRY2
ADORA1	ADORA1	KIF5B	KIF5B	WNT3	WNT3	UBE2I
FANCC	FANCC	ANXA1	ANXA1	GFRA1	GFRA1	MAPK11
GPX4	GPX4	CEBPA	CEBPA	PBX1	PBX1	PTPN12
DLL4	DLL4	PDP1	PDP1	ANXA1	ANXA1	SSTR2
NR1H4	NR1H4	PCK1	PCK1	F13A1	F13A1	MCM2
KRT1	KRT1	SLC12A5	SLC12A5	POR	POR	CA1
ABCC2	ABCC2	XPO1	XPO1	MSN	MSN	MAPKAPK2
CD38	CD38	IL4R	IL4R	FCGR3A	FCGR3A	FGR
FOLH1	FOLH1	SLC2A4	SLC2A4	CD22	CD22	PLD2
WRN	WRN	INPPL1	INPPL1	CDH5	CDH5	CARM1
GRB2	GRB2	FGF1	FGF1	GOT2	GOT2	NECTIN2
GLI1	GLI1	KCNN4	KCNN4	CYP1A1	CYP1A1	IL1B
KCNJ5	KCNJ5	MMP8	MMP8	PIK3C2A	PIK3C2A	HFE

EXT1	EXT1	RPL11	RPL11	KNG1	KNG1	A2M
MUSK	MUSK	HSP90B1	HSP90B1	CCNB1	CCNB1	MAOB
ARAF	ARAF	NEDD4	NEDD4	IMPDH1	IMPDH1	TFAM
ERCC3	ERCC3	GRB2	GRB2	GALK1	GALK1	HLA-DRB1
ANGPT2	ANGPT2	ANXA2	ANXA2	UBE2I	UBE2I	KLK6
CTSC	CTSC	NR4A1	NR4A1	OXTR	OXTR	EPO
ALDH5A1	ALDH5A1	GALK1	GALK1	CAMKK2	CAMKK2	GSTO1
DHCR7	DHCR7	ALDH1A1	ALDH1A1	CSPG4	CSPG4	GSTT1
PIK3C2A	PIK3C2A	EPHA3	EPHA3	ACVR1B	ACVR1B	ND2
YY1	YY1	KRT1	KRT1	EEF2K	EEF2K	ND1
POR	POR	PAX5	PAX5	SLC19A1	SLC19A1	IGF2R
PROS1	PROS1	NAMPT	NAMPT	SPRY2	SPRY2	GSTO2
CAPN2	CAPN2	ROR2	ROR2	PDE3A	PDE3A	MS4
APAF1	APAF1	MAT2A	MAT2A	NR2F2	NR2F2	DVL1
KITLG	KITLG	CAPN2	CAPN2	NME1	NME1	SKI
KNG1	KNG1	STK4	STK4	KLK3	KLK3	PRDM16
CTBP1	CTBP1	IMPDH2	IMPDH2	VEGFC	VEGFC	PLEKHG5
VEGFC	VEGFC	ESRRB	ESRRB	NAMPT	NAMPT	DJ1
KLK3	KLK3	MMP7	MMP7	NEDD4	NEDD4	H6PD
BIRC3	BIRC3	RRAS2	RRAS2	PDE2A	PDE2A	KIF1B
ANXA2	ANXA2	AKR1B1	AKR1B1	ATF2	ATF2	TARDBP
SCNN1B	SCNN1B	NR2F2	NR2F2	CA9	CA9	CLCN6
TMPRSS2	TMPRSS2	AHCY	AHCY	SP1	SP1	PLEKHM2
FGF1	FGF1	GABBR2	GABBR2	ARAF	ARAF	ATP13A2
CYP2C8	CYP2C8	PIN1	PIN1	IMPDH2	IMPDH2	PINK1
C5	C5	MAPK12	MAPK12	PROS1	PROS1	STMN1
CEBPA	CEBPA	SORD	SORD	FGG	FGG	KDF1
FGG	FGG	GGCX	GGCX	LPAR1	LPAR1	CCDC28B
PPM1D	PPM1D	USP9X	USP9X	DGAT1	DGAT1	YRDC
RPL5	RPL5	ARAF	ARAF	STK4	STK4	TRIT1
IL4R	IL4R	THRB	THRB	CPE	CPE	ZMPSTE24
GNAI1	GNAI1	SLC16A1	SLC16A1	GGCX	GGCX	KCNQ4
GFRA1	GFRA1	KLK3	KLK3	PTPRS	PTPRS	TMEM53
ANXA1	ANXA1	TFAP2A	TFAP2A	ROS1	ROS1	EIF2B3
SLC6A2	SLC6A2	RPA1	RPA1	CDK8	CDK8	FOXE3
PLK4	PLK4	MDH2	MDH2	MCM2	MCM2	DRS2
SIRT3	SIRT3	LPAR1	LPAR1	CYP19A1	CYP19A1	SGS
MSN	MSN	BIRC3	BIRC3	CYP2C8	CYP2C8	MEL1
SLC16A1	SLC16A1	CSNK2B	CSNK2B	TGFBR3	TGFBR3	GDH
INPPL1	INPPL1	EIF4EBP1	EIF4EBP1	MAPK12	MAPK12	CMT2A
KCNN4	KCNN4	YWHAB	YWHAB	TNFRSF17	TNFRSF17	TDP43
YWHAG	YWHAG	ITGAL	ITGAL	CA1	CA1	FRAP1
AHCY	AHCY	SYNGAP1	SYNGAP1	MAPK11	MAPK11	KIAA0214
SYNGAP1	SYNGAP1	FOLH1	FOLH1	SSTR2	SSTR2	TNFR2
TUBG1	TUBG1	SCN3A	SCN3A	ESRRB	ESRRB	CMT2A2B
TFAP2A	TFAP2A	MAPK11	MAPK11	AKR1B1	AKR1B1	SKIP
PDE2A	PDE2A	GOT2	GOT2	SLC6A2	SLC6A2	HMSN6A
TLR1	TLR1	PBX1	PBX1	ADCY3	ADCY3	ECK
MDH2	MDH2	CAMKK2	CAMKK2	YES1	YES1	CTRCT6
USP9X	USP9X	TRPV6	TRPV6	MAPKAPK2	MAPKAPK2	HOPS
RPL11	RPL11	GABRA1	GABRA1	CARM1	CARM1	TKS
DUSP6	DUSP6	GABRB3	GABRB3	PDK1	PDK1	LSD1
MAT2A	MAT2A	CDH5	CDH5	LIG1	LIG1	HPPC
C1QA	C1QA	GABRB2	GABRB2	PLD2	PLD2	AIMAH3
SLC12A5	SLC12A5	EIF4A1	EIF4A1	CSNK1E	CSNK1E	LAP18
TNK2	TNK2	NME1	NME1	FGR	FGR	C1orf172
ANGPT1	ANGPT1	CD22	CD22	AURKC	AURKC	SRC2
CASP4	CASP4	GNAI1	GNAI1	ALDH1A1	ALDH1A1	MGC1203
PAX5	PAX5	CAMK4	CAMK4	PTPRJ	PTPRJ	IRIP
ITGAL	ITGAL	ATP6V1B2	ATP6V1B2	PTPN12	PTPN12	IPT
ATP2B2	ATP2B2	ADCY3	ADCY3	SOD1	FUS	FACE1
CCNB1	CCNB1	ODC1	ODC1	FUS	SETX	DFNA2A
GABRB2	GABRB2	UBE2I	UBE2I	SNCA	DCTN1	GLYT1
GABRB3	GABRB3	SLC6A9	SLC6A9	SOD1	VAPB	NET4
CACNA1E	CACNA1E	MAP2K6	MAP2K6	LRRK2	NEFH	VWM3
MMP8	MMP8	CASP4	CASP4	SETX	VPS35	FKHL12
MMP7	MMP7	ITGB6	ITGB6	GBA1	PINK1	AAT11
TGFBR3	TGFBR3	CCNB1	CCNB1	DCTN1	PRPH	CORTRD1
NR4A1	NR4A1	CHRNA2	CHRNA2	VAPB	TARDBP	NBLST1
ROR2	ROR2	ADCY1	ADCY1	PRKN	DAO	GAMOS10
KYNU	KYNU	PLD2	PLD2	NEFH	CFAP410	CMD1LL
ATP6V1B2	ATP6V1B2	EPHA1	EPHA1	VPS35	C9orf72	LVNC8
STK4	STK4	CSNK1E	CSNK1E	FUS	CHMP2B	G6PDH
SIRT2	SIRT2	ATF2	ATF2	PINK1	PON1	CMT2A1
KCNK3	KCNK3	CA9	CA9	PARK7	UNC13A	ALS10
SLC2A4	SLC2A4	CPE	CPE	PRPH	OPTN	SKS
SORD	SORD	GABRA5	GABRA5	TARDBP	ATXN2	MSL
CSNK1E	CSNK1E	RRM2	RRM2	DAO	ANG	CMT2A2A
DGAT1	DGAT1	SPRY2	SPRY2	CFAP410	UBQLN2	TNFBR
GALK1	GALK1	CDC25B	CDC25B	SQSTM1	MAOB	CTPA
GRM5	GRM5	MCM2	MCM2	C9orf72	NR4A2	KIAA0842
XPO1	XPO1	ACVR1B	ACVR1B	CHMP2B	ATP13A2	ARCC2
EIF4EBP1	EIF4EBP1	PTPN12	PTPN12	PON1	NEFL	CTPP1
GABRA1	GABRA1	FGR	FGR	UNC13A	CHCHD10	HPPI
CHRNA2	CHRNA2	SSTR2	SSTR2	OPTN	GLT8D1	HPPA
RRAS2	RRAS2	EEF2K	EEF2K	ATXN2	ANXA11	TNSALP
GABBR2	GABBR2	CA12	CA12	ANG	FIG4	HPPO
THRB	THRB	MAPKAPK2	MAPKAPK2	VCP	PFN1	KIAA0601
CDC25B	CDC25B	PTK6	PTK6	MAPT	NEK1	AOF2
NR2F2	NR2F2	CA1	CA1	GDNF	CCNF	ECTD12
MAPK12	MAPK12	CARM1	CARM1	PPARGC1A	MATR3	CPRF
SLC6A9	SLC6A9	SNCA	VPS35	VAPB	KIF5A	SMN
MC1R	MC1R	LRRK2	PINK1	TH	ALS2	BHC110
FOXA2	FOXA2	GBA1	MAOB	UBQLN2	SPG11	COXPD35
ATF2	ATF2	PRKN	NR4A2	MAOB	SYNJ1	STE24
CPE	CPE	VPS35	ATP13A2	BDNF	SNCB	MADB
SCN3A	SCN3A	PINK1	TARDBP	NR4A2	IL1B	RSDM1
ODC1	ODC1	PARK7	SYNJ1	PRKN	TFAM	GCENSG
HSP90B1	HSP90B1	MAPT	SNCB	SLC6A3	EWSR1	CTDI
ADCY1	ADCY1	GDNF	IL1B	ERBB4	TAF15	ASMD
NAMPT	NAMPT	TH	TFAM	TBK1	SS18L1	IS6
PBX1	PBX1	MAOB	GSTM1	DDC	GLE1	CTRCT34
MAP2K6	MAP2K6	BDNF	DNAJC6	SLC18A2	SCFD1	ASGD2
CD22	CD22	NR4A2	FBXO7	ATP13A2	GSTM1	
PDK1	PDK1	PRKN	VPS13C	PPARGC1A	DPP6	
ITGB6	ITGB6	SLC6A3	TMEM230	UCHL1	DNAJC6	
GABRA5	GABRA5	DDC	BST1	NEFL	FBXO7	
NME1	NME1	SLC18A2	DRD1	CHCHD10	DNAJC7	
EPHA3	EPHA3	ATP13A2	HLA-DRA	GLT8D1	ARHGEF28	
AKR1B1	AKR1B1	PPARGC1A	HNMT	ANXA11	VPS13C	
ESRRB	ESRRB	UCHL1	NAT2	FIG4	PON2	
YWHAB	YWHAB	SOD1	ANG	PFN1	SARM1	
NEDD4	NEDD4	DRD2	DNAJC13	NEK1	TMEM230	
THRA	THRA	TARDBP	HTRA2	CCNF	BST1	
EEF2K	EEF2K	SYNJ1	HTT	MATR3	DRD1	
CA9	CA9	SNCB	KLK6	KIF5A	SIGMAR1	
GOT2	GOT2	IL1B	CYP1A2	ALS2	HLA-DRA	
ALDH1A1	ALDH1A1	TFAM	GAK	SPG11	TREM2	
IMPDH2	IMPDH2	GSTP1	HFE	SOD1	HNMT	
ADCY3	ADCY3	CYP2D6	RAB32	SETX	NAT2	
CDK8	CDK8	GSTM1	CYP2E1	NFE2L2	DNAJC13	
EIF4A1	EIF4A1	HSPA9	SNCAIP	DRD2	HTRA2	
EPHA1	EPHA1	INS	EPO	TARDBP	HTT	
CAMK4	CAMK4	PARK7	MAPT-AS1	SYNJ1	KLK6	
LIG1	LIG1	IL6	GIGYF2	SNCB	HNRNPA2B1	
CAMKK2	CAMKK2	TNF	SNCA-AS1	IL1B	CYP1A2	
CDH5	CDH5	NOS1	GSTT1	TFAM	SPTLC1	
CALCR	CALCR	NQO1	HLA-DRB1	EWSR1	MIR206	
SPRY2	SPRY2	GFAP	TRPM2	TAF15	GAK	
UBE2I	UBE2I	MAOA	GDF5	SS18L1	HFE	
CSPG4	CSPG4	NGF	EPHX1	GLE1	RAB32	
PTPRJ	PTPRJ	ABCB1	GRK6	SCFD1	CYP2E1	
MAPK11	MAPK11	DNAJC6	GSTO2	GSTP1	UBB	
PRKCH	PRKCH	FBXO7	EN1	CYP2D6	SNCAIP	
PTPN12	PTPN12	PLA2G6	NQO2	ALS2	EPO	
SSTR2	SSTR2	VPS13C	CAST	GSTM1	MAPT-AS1	
MCM2	MCM2	SOD2	GSTO1	HSPA9	GIGYF2	
CA12	CA12	TMEM230	BAG5	DPP6	SNCA-AS1	
PTPRS	PTPRS	IGF2	SLC30A10	INS	GOT1	
CA1	CA1	PINK1	DDIT4	HNRNPA1	GSTT1	
ST14	ST14	BST1	GSTA4	PARK7	HLA-DRB1	
MAPKAPK2	MAPKAPK2	DRD1	AIF1	IL6	CAV2	
FGR	FGR	HLA-DRA	NDUFV2	TNF	MOBP	
PLD2	PLD2	PARP1	ND3	NOS1	TNIP1	
ADORA2B	ADORA2B	HNMT	ND4	NQO1	TRPM2	
YES1	YES1	NAT2	ATP6	GFAP	GDF5	

	PTK6	PTK6	ANG	ND1	GFAP	EPHX1
	CARM1	CARM1	HSPA8	ND2	MAOA	GRK6
**DisGeNET**	APP	TOMM40	POLG	A2M	NGF	AOX1
	PSEN1	NECTIN2	CYP1A1	RFC1	ABCB1	GSTO2
	APOE	ABCA7	AKT1	BSN	DNAJC6	EN1
	MAPT	TREM2	DNAJC13	TGM6	FBXO7	NQO2
	PSEN2	BIN1	HTRA2	TNR	GRN	ITPR2
	GRN	PICALM	HTT	FCER2	FIG4	CAST
	ACE	CD33	SLC11A2	HBG1	DNAJC7	GSTO1
	TOMM40	APOC1	KLK6	IGF2R	ARHGEF28	ARPP21
	PPARG	IL1B	MAPK1	MAP2	FUS	ELP3
	CLU	BACE1	DBH	MIR181C	PLA2G6	DYNC1H1
	NECTIN2	AGER	CYP1A2	MAG	VPS13C	BAG5
	ABCA7	SORL1	CP	MTA1	SOD2	MIR23A
	GSK3B	HFE	GAK	RIC3	TUBA4A	SLC30A10
	ACHE	A2M	HFE	PACRG	PON2	DDIT4
	TREM2	MAOB	ADORA2A	NECTIN2	PTGS2	MFN1
	BIN1	CYP46A1	HSPA1A	SEPTIN14	SARM1	GSTA4
	PICALM	TFAM	MTHFR	PHACTR2	TMEM230	AIF1
	CD33	KLC1	HMOX1	MTRR	IGF2	NDUFV2
	APOC1	HLA-DRB1	RAB32	CHRNB3	PINK1	CCS
	BDNF	NTF3	CYP2E1	CNTNAP2	BST1	ND3
	IGF1	BACE2	SNCAIP	NCAPG2	DRD1	ND4
	IL1B	CHRNA7	ALDH2	ADARB2	SLC1A2	ATP6
	ADAM10	PILRA	DNM1L	DRAXIN	SIGMAR1	ND1
	BACE1	LEP	SNCA	DEFA4	HLA-DRA	ND2
	BCHE	MS4A4A	EPO	RPL6	TREM2	APEX1
	TNF	APH1B	ESR2	PODXL	PARP1	A2M
	AGER	CRH	MAPT-AS1	COL19A1	HNMT	RFC1
	SNCA	ABI3	PRKN	TALDO1	NAT2	BSN
	SORL1	LRP8	GIGYF2	CEACAM6	VDR	TGM6
	TTR	IGFBP3	CASP3	TCN2	ANG	TNR
	HFE	S100B	SNCA-AS1	LOC106627981	HSPA8	RNASE4
	NOS3	IL4	VDR	KCNK18	POLG	PON3
	A2M	HTR6	GSTT1	KCNJ4	CYP1A1	NIPA1
	MAOB	IL10	HLA-DRB1	RPL14	GSK3B	FCER2
	CYP46A1	EIF2S1	LRRK2	FGB	AKT1	HBG1
	TFAM	IL2	SYNJ1	RPS8	DNAJC13	IGF2R
	VEGFA	CD2AP	VPS13C	SLC38A2	HTRA2	MAP2
	KLC1	CST3	TRPM2	RPL23A	HTT	MIR181C
	MME	CASS4	GDF5	THAP1	SLC11A2	MAG
	ADAM17	INPP5D	ATM	CATIP-AS2	KLK6	MIR214
	HLA-DRB1	NPY	EPHX1	PNKD	HNRNPA2B1	TNFRSF21
	NTF3	RELN	GRK6	BSCL2	MAPK1	MTA1
	BACE2	WWOX	GSTO2	PINK1-AS	DBH	ERLIN1
	INS	APBB1	EN1	HNRNPUL2-BSCL2	CAV1	RIC3
	CHRNA7	KLK6	NQO2	PTGIS	CYP1A2	PACRG
	PILRA	IL1A	MAPK3	BCC1	SPTLC1	NECTIN2
	IDE	APOA4	CASP9	CMM	CP	NUP50
	LEP	MT3	CAST	CTRCT8	MIR206	DBR1
	MS4A4A	PPARA	GSTO1	DEL1p36	GAK	SLC6A1
	APH1B	ADRA1A	CREB1	IBD7	HFE	PCDHA9
	CRH	RCAN1	TNK2	MS4	ADORA2A	SEPTIN14
	ABI3	PON1	INSR	PSORS7	HSPA1A	PHACTR2
	ESR1	CLOCK	BAG5	ISG15	MTHFR	MTRR
	PLCG2	DLST	ENO2	AGRN	SOD2	CHRNB3
	ICAM1	GCG	SLC30A10	TNFRSF4	HMOX1	TXNRD1
	IRS1	SERPINE2	HGF	B3GALT6	RAB32	SOD1-DT
	NFE2L2	MIR146A	MAP3K5	SCNN1D	CYP2E1	KANK1
	LRP8	ARC	DDIT4	INTS11	UBB	BICD2
	PLAU	TSPAN14	DNAJC6	DVL1	SNCAIP	ABCA7
	IGFBP3	DPYSL2	GSTA4	ATAD3A	ALDH2	PNPLA6
	CHAT	VSNL1	AIF1	MIB2	DNM1L	CNTNAP2
	S100B	DHCR24	NDUFV2	GNB1	TP53	NCAPG2
	MAOA	NCK2	ND3	SKI	SNCA	ADARB2
	EIF2AK2	MIR124-3	ND4	RER1	EPO	ERCC6L2
	CDK5	LRPAP1	ATP6	PEX10	ESR2	NRF1
	TLR4	EPO	ND1	PANK4	APOE	MIR424
	IL4	GSTO1	ND2	TNFRSF14	MAPT-AS1	CAMK1G
	LRP1	GSTT1	HMGCR	PRDM16	PRKN	PPARGC1B
	UCHL1	CALM1	EIF2AK2	TP73	GIGYF2	DRAXIN
	HTR6	ND2	A2M	SMIM1	CASP3	DEFA4
	IL10	PON3	RFC1	NPHP4	SNCA-AS1	RPL6
	CSF1R	UQCRC1	BSN	CHD5	GOT1	PODXL
	MPO	ND1	TGM6	ICMT	VDR	COL19A1
	SOD2	ECE2	TNR	ESPN	GSTT1	ESRRA
	ENO1	TPP1	FCER2	TNFRSF25	HLA-DRB1	TALDO1
	NCSTN	PCDH11X	HBG1	PLEKHG5	NRG1	CEACAM6
	PRNP	ATP5F1A	IGF2R	PHF13	DCTN1	CLEC4C
	EIF2S1	IGF2R	MAP2	CAMTA1	ANG	LGALSL
	CYP2D6	ADAMTS1	MIR181C	SCZD12	SPG11	TCN2
	APOA1	IQCK	MAG	TNFRSF9	PRPH	LOC106627981
	LDLR	GAPDHS	MTA1	DJ1	LRRK2	EGILA
	IL2	CHRNB2	RIC3	MIG6	CAV2	SCAF4
	ECE1	GSTM3	GBA1	MIR34A	MOBP	KCNK18
	ABCA1	ABAT	PACRG	H6PD	SYNJ1	LOC124629354
	ESR2	MBL2	IGF1R	KIF1B	TNIP1	TROAP-AS1
	CD2AP	NEFM	NECTIN2	CORT	VPS13C	DDX20
	EPHA1	PPP2R2B	FBP1	CASZ1	TRPM2	CHRNB4
	CST3	PPP3R1	SEPTIN14	CIROZ	GDF5	LOC130008791
	CASS4	IREB2	PHACTR2	MASP2	ATM	LOC105371050
	IGF2	LIPC	MTRR	UBIAD1	EPHX1	LOC108903148
	INPP5D	PON2	CHRNB3	CLCN6	GRK6	LOC130056709
	BAX	TPH1	CNTNAP2	NPPA	GSK3A	LOC126807526
	NPY	CDK5R1	NCAPG2	NPPB	AOX1	LOC126860782
	INSR	PGRMC1	ADARB2	PLOD1	GSTO2	CAPN14
	RELN	TPI1	DRAXIN	TNFRSF8	EN1	LOC101929270
	BCL2	SLC30A4	DEFA4	GBD2	NQO2	CHRNA3
	PTK2B	SLC30A6	RPL6	EFHD2	MAPK3	LOC130055269
	HMOX1	MIR375	PODXL	CTRC	CASP9	UBQLN4
	IGF1R	PYY	COL19A1	CELA2A	HMGB1	RXRA
	WWOX	HSPA1B	EDN1	PLEKHM2	ITPR2	SELPLG
	CASP3	APBB2	TALDO1	ANIB3	CAST	BSG
	GRIN2B	PNMT	CEACAM6	CLCNKA	GSTO1	TLE3
	HMGCR	GRIN2C	SNCA	CLCNKB	CREB1	WNT7A
	APBB1	GSTO2	SNCA	PADI4	ARPP21	INA
	HSPA1A	UNC13C	VPS13C	PADI6	TNK2	KCNJ4
	KLK6	SPRED2	VPS13C	PAX7	SPG11	RPL14
	STAT3	AMFR	TCN2	ALDH4A1	SIGMAR1	FGF6
	NTRK1	C2	DNAJC6	UBR4	ELP3	GBX2
	NTRK2	GSS	DNAJC6	EMC1	DYNC1H1	TIAM1
	IL1A	TAP2	LOC106627	PLA2G5	INSR	CD7
	VLDLR	CCDC6	KCNK18	DDOST	BAG5	LOC126860783
	CTSD	MIR100	KCNJ4	WNT4	MIR23A	LOC130056971
	AGTR1	APP-DT	RPL14	C1QC	MFN2	FGB
	FYN	XRN1	FGB	C1QB	ENO2	RPS8
	GLUL	ADRB3	RPS8	HNRPR	SLC30A10	SIX2
	APOA4	LOC126653330	SLC38A2	ID3	HGF	DBX1
	MT3	HFE-AS1	RPL23A	LYPLA2	MAP3K5	CNTF
	NOS1	MIR296	PACRG	GALE	DDIT4	GDI1
	PPARA	MIR708	THAP1	HMGCL	DNAJC6	LAT
	GAPDH	DNS	CATIP-AS2	CNR2	MFN1	KIF3C
	ADRA1A	BCC1	PNKD	MYOM3	GSTA4	PDGFA
	RCAN1	CMM	BSCL2	IFNLR1	AIF1	SHC1
	NGFR	CTRCT8	PINK1-AS	GRHL3	NDUFV2	SERPINA3
	PON1	DEL1p36	PARK7	RUNX3	CCS	CST3
	CLOCK	IBD7	HNRNPUL2-	RHD	ND3	HSF1
	PARP1	MS4	PTGIS	RHCE	ND4	CALB2
	CYP19A1	PSORS7	BCC1	LDLRAP1	ATP6	SLC38A2
	DLST	ISG15	CMM	SELENON	ND1	RPL23A
	GCG	AGRN	CTRCT8	STMN1	ND2	LOC130056973
	SERPINE2	TNFRSF4	DEL1p36	TRIM63	CYLD	LOC130066253
	C3	B3GALT6	IBD7	CD52	APEX1	GSX2
	IL6R	SCNN1D	MS4	DHDDS	HMGCR	FMO1
	LPL	INTS11	PSORS7	PIGV	EIF2AK2	OTOG
	VCP	DVL1	ISG15	GPATCH3	A2M	JUND
	MIR146A	ATAD3A	AGRN	NR0B2	MTHFR	MT1A
	TF	MIB2	TNFRSF4	KDF1	RFC1	PENK
	ARC	GNB1	B3GALT6	FCN3	BSN	SNAI1
	TSPAN14	SKI	SCNN1D	GPR3	SPTLC2	TMSB4X
	DPYSL2	RER1	INTS11	RPA2	TGM6	CD68
	VSNL1	PEX10	DVL1	SMPDL3B	TNR	ACTL6B
	DHCR24	PANK4	ATAD3A	XKR8	RNASE4	MIR455
	F2	TNFRSF14	MIB2	PTAFR	PON3	THAP1
	MTHFR	PRDM16	GNB1	SNHG12	NIPA1	SYNE1
	NCK2	TP73	SKI	MECR	TARDBP	CATIP-AS2

	MIR124-3	SMIM1	RER1	MATN1	FCER2	PNKD
	FAS	NPHP4	PEX10	SDC3	HBG1	BSCL2
	LRPAP1	CHD5	PANK4	PUM1	IGF2R	PINK1-AS
	EPO	ICMT	TNFRSF14	CCDC28B	MAP2	PLEKHG5
	GSTO1	ESPN	PRDM16	LCK	MIR181C	TRPM7
	ALDH2	TNFRSF25	TP73	SYNC1	MAG	HNRNPUL2-BSCL2
	GSTT1	PLEKHG5	SMIM1	YARS1	MIR214	PTGIS
	GSTP1	PHF13	NPHP4	FNDC5	PLA2G4A	LOC129935420
	CALM1	CAMTA1	CHD5	HPCA	TNFRSF21	RAPGEF2
	ND2	SCZD12	ICMT	AK2	MTA1	MS4
	PON3	TNFRSF9	ESPN	TRIM62	SPTLC1	PERM1
	UQCRC1	DJ1	TNFRSF25	GBD3	GSTP1	DVL1
	ND1	MIG6	PLEKHG5	GJB4	CD40LG	SKI
	ECE2	MIR34A	PHF13	GJB3	GSR	PRDM16
	TPP1	H6PD	CAMTA1	GJA4	ERLIN1	DJ1
	PCDH11X	KIF1B	SCZD12	COL8A2	RIC3	H6PD
	ATP5F1A	CORT	TNFRSF9	ZC3H12A	GBA1	KIF1B
	IGF2R	CASZ1	DJ1	SNIP1	PACRG	CLCN6
	ADAMTS1	CIROZ	MIG6	DNALI1	IGF1R	PLEKHM2
	IQCK	TARDBP	ENO1	RSPO1	NECTIN2	ALPL
	GAPDHS	MASP2	MIR34A	EPHA10	NUP50	STMN1
	CHRNB2	UBIAD1	H6PD	YRDC	DBR1	ARID1A
	GSTM3	CLCN6	PIK3CD	UTP11L	SLC6A1	KDF1
	ABAT	NPPA	NMNAT1	RRAGC	FBP1	TRNP1
	MBL2	NPPB	KIF1B	TRIT1	PCDHA9	CCDC28B
	NEFM	PLOD1	CORT	CAP1	SEPTIN14	MACST
	PCK1	TNFRSF8	CASZ1	PPT1	PHACTR2	DLGAP3
	PIK3R1	GBD2	CIROZ	ZMPSTE24	MTRR	SFPQ
	PPP2R2B	EFHD2	TARDBP	COL9A2	CHRNB3	NCDN
	CASP7	CTRC	MASP2	KCNQ4	TFAM	YRDC
	PPP3R1	CELA2A	MTOR	CTPS1	TXNRD1	TRIT1
	IREB2	PLEKHM2	UBIAD1	EDN2	NEFH	MFSD2A
	LIPC	ANIB3	MTHFR	GUCA2B	SOD1-DT	ZMPSTE24
	MCM2	CLCNKA	CLCN6	PPCS	KANK1	KCNQ4
	PON2	CLCNKB	NPPA	CLDN19	CHRNA4	SZT2
	TPH1	ATP13A2	NPPB	P3H1	BICD2	RNF220
	WT1	PADI4	PLOD1	SVBP	CACNA1A	TMEM53
	CDK5R1	PADI6	MFN2	ERMAP	ABCA7	EIF2B3
	PGRMC1	PAX7	TNFRSF8	CFAP57	PNPLA6	STIL
	TPI1	ALDH4A1	TNFRSF1B	TIE	CNTNAP2	FOXE3
	SLC2A4	UBR4	GBD2	MPL	NCAPG2	DMRTA2
	SLC30A4	EMC1	EFHD2	CDC20	ADARB2	ORC1
	SLC30A6	PLA2G5	CTRC	ELOVL1	ERCC6L2	FGGY
	MIR375	PINK1	CELA2A	PTPRF	NRF1	NFIA
	PYY	DDOST	PLEKHM2	PTOS1	MIR424	RPE65
	HSPA1B	WNT4	ANIB3	ST3GAL3	CAMK1G	PIGK
	APBB2	C1QC	CLCNKA	DPH2	PPARGC1B	TMED5
	PNMT	C1QB	CLCNKB	TMEM53	DRAXIN	ARHGAP29
	GRIN2C	HNRPR	EPHA2	PTCH2	SMARCA4	WS2B
	GSTO2	ID3	ATP13A2	EIF2B3	DEFA4	FRRS1
	UNC13C	LYPLA2	SDHB	UROD	RPL6	SASS6
	SPRED2	GALE	PADI4	HPDL	PODXL	S1PR1
	AMFR	HMGCL	PADI6	MUTYH	COL19A1	COL11A1
	C2	CNR2	PAX7	MMACHC	EDN1	SLC25A24
	GSS	MYOM3	ALDH4A1	POMGNT1	ESRRA	GPSM2
	TAP2	IFNLR1	UBR4	RAD54L	TALDO1	SARS1
	CCDC6	GRHL3	EMC1	CYP4A11	CEACAM6	GNAI3
	MIR100	RUNX3	PLA2G2A	CYP4A22	SNCA	ALX3
	KCNMA1	RHD	PLA2G5	TAL1	SLC11A2	KCNA3
	APP-DT	RHCE	PINK1	CMPK1	SNCA	RAP1A
	NCF2	LDLRAP1	DDOST	FOXE3	CLEC4C	WNT2B
	XRN1	SELENON	ECE1	ELAVL4	LGALSL	CAPZA1
	ADRB3	STMN1	ALPL	PARK10	VPS13C	MAGI3
	LOC126653	TRIM63	HSPG2	FAF1	VPS13C	PTPN22
	HFE-AS1	CD52	CDC42	RNF11	TCN2	VANGL1
	MIR296	DHDDS	WNT4	LRP8	DNAJC6	NHLH2
	MIR708	PIGV	C1QA	NDC1	DNAJC6	CD58
**OMIM**	DNS	GPATCH3	C1QC	DIO1	SOD1-DT	IGSF3
	BCC1	NR0B2	C1QB	TMEM59	LOC106627981	PDZK1
	CMM	KDF1	EPHB2	CDCP2	EGILA	RBM8A
	CTRCT8	FCN3	KDM1A	PARS2	RNASE4	POLR3GL
	DEL1p36	GPR3	HNRPR	SJA	SCAF4	ECM1
	IBD7	RPA2	ID3	MLM	KCNK18	SCNM1
	MS4	SMPDL3B	RPL11	CCV	UNC13A	RORC
	PSORS7	XKR8	LYPLA2	C1DELp36	LOC124629354	EFNA3
	ISG15	PTAFR	GALE	DNS	TROAP-AS1	EFNA1
	AGRN	SNHG12	HMGCL	IMD16	DDX20	DPM3
	TNFRSF4	MECR	FUCA1	IMD38	CHRNB4	GON4L
	B3GALT6	MATN1	CNR2	G1P2	LOC130008791	SPTA1
	SCNN1D	SDC3	MYOM3	CMS8	LOC105371050	KCNJ10
	INTS11	PUM1	IFNLR1	TXGP1L	LOC108903148	VANGL2
	DVL1	CCDC28B	GRHL3	SEMDJL1	EGILA	SLAMF1
	ATAD3A	SYNC1	RUNX3	IFI15	LOC130056709	CD244
	MIB2	YARS1	RHD	PSF3L	VCP	F11R
	GNB1	FNDC5	RHCE	DRS2	TROAP-AS1	MPZ
	SKI	HPCA	LDLRAP1	HAYOS	LOC126807526	NOS1AP
	RER1	AK2	SELENON	NEDMLOB	LOC126860782	DDR2
	PEX10	TRIM62	STMN1	MRD42	CAPN14	LMX1A
	PANK4	GBD3	TRIM63	SGS	LOC101929270	TMCO1
	TNFRSF14	GJB4	CD52	PBD6B	CHRNA3	GPR161
	PRDM16	GJB3	DHDDS	NALD	LOC130055269	F5
	TP73	GJA4	PIGV	CTRCT49	CTSD	KIFAP3
	SMIM1	COL8A2	GPATCH3	HVEM	FOS	GORAB
	NPHP4	ZC3H12A	NR0B2	MEL1	GRIA3	PRRX1
	CHD5	SNIP1	KDF1	CILD47	PON1	DARS2
	ICMT	DNALI1	SLC9A1	VEL	UBQLN4	PDCN
	ESPN	RSPO1	FCN3	SLSN4	RXRA	ACBD6
	TNFRSF25	EPHA10	GPR3	PMNDS	SELPLG	ASPM
	PLEKHG5	YRDC	FGR	IMD109	BSG	CRB1
	PHF13	UTP11L	RPA2	USH1M	TLE3	DDX59
	CAMTA1	RRAGC	SMPDL3B	TNFRSF12	WNT7A	CNTN2
	SCZD12	TRIT1	XKR8	KIAA0720	INA	IL10
	TNFRSF9	CAP1	PTAFR	SPOC1	KCNJ4	MCP
	DJ1	PPT1	SNHG12	KIAA0833	RPL14	HHAT
	MIG6	ZMPSTE24	MECR	HTX14	CLU	DEL1q41q42
	ENO1	COL9A2	MATN1	ILA	FGF6	USH2A
	MIR34A	KCNQ4	SDC3	PARK7	GABRA1	TLR5
	H6PD	CTPS1	PUM1	RALT	GBX2	DEGS1
	PIK3CD	EDN2	CCDC28B	PPH	JAK3	WDR26
	NMNAT1	GUCA2B	LCK	MIRN34A	BCL2L1	LBR
	KIF1B	PPCS	HDAC1	GDH	TIAM1	TMEM63A
	CORT	CLDN19	SYNC1	APDS	CD7	LEFTB
	CASZ1	P3H1	YARS1	NMNAT	LOC126860783	CCSAP
	CIROZ	SVBP	FNDC5	CMT2A	LOC130056971	DISC1
	TARDBP	ERMAP	HPCA	SHILCA	FGB	TBCE
	MASP2	CFAP57	AK2	SRG	RPS8	EDARADD
	MTOR	TIE	TRIM62	C1orf127	SIX2	MTR
	UBIAD1	CDC20	GBD3	TDP43	DBX1	GREM2
	MTHFR	ELOVL1	GJB4	ROCHIS	CNTF	COLEC11
	CLCN6	PTOS1	GJB3	FRAP1	GDI1	SOX11
	NPPA	ST3GAL3	GJA4	TERE1	LAT	KIDINS220
	NPPB	DPH2	COL8A2	ATFB6	KIF3C	TYHAQ
	PLOD1	TMEM53	ZC3H12A	CLCN6	PDGFA	PDIA6
	MFN2	PTCH2	SNIP1	PND	SHC1	NBAS
	TNFRSF8	EIF2B3	DNALI1	BNP	TNF	MSGN1
	TNFRSF1B	UROD	RSPO1	LH1	CASP1	MATN3
	GBD2	HPDL	EPHA10	KIAA0214	SERPINA3	EIF2B4
	EFHD2	MUTYH	YRDC	CD30	CST3	FOSL2
	CTRC	MMACHC	UTP11L	TNFR2	GFAP	SPAST
	CELA2A	POMGNT1	RRAGC	MSL	HSF1	PKDCC
	PLEKHM2	RAD54L	TRIT1	SWS1	XIAP	DYNC2LI1
	ANIB3	CYP4A11	CAP1	CLCR	LDLR	SIX3
	CLCNKA	CYP4A22	PPT1	ELA2A	CALB2	CRIPT
	CLCNKB	TAL1	ZMPSTE24	SKIP	LOC126860782	RTN4
	EPHA2	CMPK1	COL9A2	ATRST2	SLC38A2	EFEMP1
	ATP13A2	FOXE3	KCNQ4	CMT2A2B	RPL23A	WDPCP
	SDHB	ELAVL4	CTPS1	CTRCT6	LOC130056973	SPRED2
	PADI4	PARK10	EDN2	ECK	LOC130066253	ANTXR1
	PADI6	FAF1	GUCA2B	PARK9	CREBBP	TIA1
	PAX7	RNF11	PPCS	SDH2	GSX2	ACTG2
	ALDH4A1	NDC1	CLDN19	PADI5	FMO1	DGUOK
	UBR4	DIO1	P3H1	OZEMA16	OTOG	SLC4A5
	EMC1	TMEM59	SVBP	RMS2	JUND	SUCLG1
	PLA2G2A	CDCP2	ERMAP	ALDH4	MT1A	RPIA
	PLA2G5	PARS2	SLC2A1	ZUBR1	PENK	IGKC
	PINK1	NEDMLOB	CFAP57	KIAA0090	SNAI1	CNGA3

DDOST	OX40	TIE	PLA2B	TMSB4X	RANBP2
ECE1	EDSSPD2	MPL	FRFB	VIM	POLR1B
ALPL	ACT35	CDC20	PARK6	CD68	CKAP2L
HSPG2	ALGAZ	ELOVL1	OST	ACTL6B	IL1A
CDC42	MLM	PTPRF	ARCC2	MIR455	DPP10
WNT4	CCV	PTOS1	HOPS	PACRG	CFAP221
C1QA	C1DELp36	ST3GAL3	PLC	THAP1	EPB41L5
C1QC	G1P2	DPH2	TKS	SYNE1	RNU4ATAC
C1QB	CMS8	SLC6A9	SERKAL	CATIP-AS2	CFC1
EPHB2	TXGP1L	TMEM53	C1QD1	PNKD	DARS1
KDM1A	SEMDJL1	PTCH2	C1QG	BSCL2	LRP1B
HNRPR	IMD38	EIF2B3	C1QD2	PINK1-AS	GEFSP7
ID3	PSF3L	UROD	EPHT3	PLEKHG5	MBD5
RPL11	DRS2	HPDL	LSD1	TRPM7	MMADHC
LYPLA2	HAYOS	MUTYH	HNRNPR	VRK1	ITGB6
GALE	IMD16	MMACHC	HPPA	PARK7	SLC4A10
HMGCL	MRD42	PRDX1	DBA7	HNRNPUL2-BS	GALNT3
FUCA1	SGS	POMGNT1	APT2	ERLIN1	BBS5
CNR2	IFI15	RAD54L	THC13	PTGIS	SP9
MYOM3	NALD	CYP4A11	VWS2	LOC129935420	GPR155
IFNLR1	CTRCT49	CYP4A22	AML2	LOC130066253	CHN1
GRHL3	HVEM	TAL1	CB2	RAPGEF2	HOXD13
RUNX3	MEL1	CMPK1	CMH31	MS4	PDE11A
RHD	CILD47	FOXE3	IL28RA	PERM1	FRZB
RHCE	VEL	ELAVL4	SOM	DVL1	STAT4
LDLRAP1	SLSN4	PARK10	CBFA3	SKI	STK17B
SELENON	PMNDS	FAF1	HDFNRH	PRDM16	SATB2
STMN1	RC68	RNF11	RHNA	DJ1	NIF3L1
TRIM63	USH1M	CPT2	ARH	H6PD	HPPC
CD52	TNFRSF12	LRP8	SEPN1	KIF1B	C1orf170
DHDDS	KIAA0720	NDC1	LAP18	TARDBP	DRS2
PIGV	SPOC1	DIO1	RNF28	MTOR	SGS
GPATCH3	KIAA0833	TMEM59	CDW52	CLCN6	MEL1
NR0B2	PHRINL	CDCP2	HDS	MFN2	PARK7
KDF1	ILA	PARS2	HPMRS1	TNFRSF1B	GDH
SLC9A1	PARK7	SJA	THMA2	PLEKHM2	CMT2A
FCN3	RALT	MLM	SHP	EPHA2	TDP43
GPR3	PPH	CCV	C1orf172	ALPL	FRAP1
FGR	MIRN34A	C1DELp36	NHE1	CDC42	CLCN6
RPA2	GDH	DNS	HAKA1	KDM1A	KIAA0214
SMPDL3B	APDS	IMD16	MURF1	STMN1	TNFR2
XKR8	NMNAT	IMD38	SRC2	ARID1A	SKIP
PTAFR	CMT2A	G1P2	DEDSM	KDF1	ECK
SNHG12	MDS	CMS8	ASML3B	TRNP1	HOPS
MECR	SRG	TXGP1L	LIKNS	FGR	TKS
MATN1	C1orf127	SEMDJL1	OPA16	CCDC28B	LSD1
SDC3	TDP43	IFI15	LINC001000	MACST	LAP18
PUM1	PBD6A	PSF3L	NRBF1	DLGAP3	C1orf4
CCDC28B	FRAP1	DRS2	CRTM	SFPQ	C1orf172
LCK	TERE1	HAYOS	SYND3	NCDN	PGR22
HDAC1	PBD6B	NEDMLOB	KIAA0099	YRDC	CHN
SYNC1	CLCN6	MRD42	MGC1203	TRIT1	HOX4I
YARS1	PND	SGS	IMD22	MFSD2A	PDE11A1
FNDC5	BNP	PBD6B	RPD3L1	ZMPSTE24	CD49D
HPCA	LH1	NALD	YTS	KCNQ4	FRZB1
AK2	KIAA0214	CTRCT49	YARS	SZT2	EDSVASC
TRIM62	CD30	HVEM	FRCP2	SLC6A9	SLEB11
GBD3	TNFR2	MEL1	DYT2	RNF220	DRAK2
GJB4	CMT2A2B	CILD47	IMNEPD2	TMEM53	KIAA1034
GJB3	SWS1	VEL	DEAR1	EIF2B3	CMD1LL
GJA4	CLCR	SLSN4	YRS	STIL	CORTRD1
COL8A2	ELA2A	PMNDS	CX30.3	FOXE3	NBLST1
ZC3H12A	SKIP	IMD109	CX31	DMRTA2	LVNC8
SNIP1	ATFB6	USH1M	CX37	ORC1	AIMAH3
DNALI1	EDSKCL1	TNFRSF12	FECD1	FGGY	G6PDH
RSPO1	HMSN6A	KIAA0720	MCPIP	NFIA	CMT2A1
EPHA10	ECK	SPOC1	NEDHCS	RPE65	ALS10
YRDC	PARK9	KIAA0833	P28	PIGK	SKS
UTP11L	SDH2	HTX14	FLJ40906	PRKACB	MSL
RRAGC	PADI5	ILA	DFNA88	TMED5	CMT2A2A
TRIT1	OZEMA16	PARK7	IRIP	ARHGAP29	TNFBR
CAP1	RMS2	RALT	CGI94	WS2B	KIAA0842
PPT1	ALDH4	PPH	RAGC	FRRS1	CTPP1
ZMPSTE24	ZUBR1	MIRN34A	IPT	SASS6	TNSALP
COL9A2	KIAA0090	GDH	DYT18	S1PR1	CTRCT6
KCNQ4	PLA2B	APDS	CLN1	COL11A1	AOF2
CTPS1	FRFB	NMNAT	FACE1	SLC25A24	SMN
EDN2	PARK6	CMT2A	EDM2	GPSM2	B120
GUCA2B	OST	SHILCA	DFNA2A	SARS1	ECTD12
PPCS	CTRCT6	SRG	CTPS	GNAI3	CTRCT34
CLDN19	HOPS	C1orf127	OI8	ALX3	CSS2
P3H1	PLC	TDP43	UGN	KCNA3	MRD14
SVBP	TKS	ROCHIS	CMD2C	RAP1A	SMARCF1
ERMAP	SERKAL	FRAP1	HOMG5	WNT2B	CTPA
SLC2A1	C1QD1	TERE1	LEPRE1	CAPZA1	HPPA
CFAP57	C1QG	ATFB6	CCDC23	SLC16A1	NEDIES
TIE	C1QD2	CLCN6	SC	MAGI3	GAMOS10
MPL	EPHT3	PND	GLUT1	PTPN22	COXPD35
CDC20	LSD1	BNP	WDR65	NRAS	AAT11
ELOVL1	HNRNPR	LH1	JTK14	VANGL1	STE24
PTPRF	HPPC	KIAA0214	TPOR	NHLH2	MADB
PTOS1	DBA7	CD30	OZEMA14	CD58	DEE18
ST3GAL3	APT2	TNFR2	SSC1	IGSF3	GCENSG
DPH2	THC13	MSL	LAR	PDZK1	ASGD2
SLC6A9	BDPLT22	SWS1	MRT12	RBM8A	CTDI
TMEM53	AIMAH3	CLCR	SIAT6	POLR3GL	IS6
PTCH2	CB2	ELA2A	DPH2L2	ECM1	MCPH7
EIF2B3	CX5	SKIP	GLYT1	SCNM1	ASMD
UROD	IL28RA	ATRST2	NET4	RORC	CMT2A2B
HPDL	SOM	CMT2A2B	IS6	EFNA3	HMSN6A
MUTYH	CBFA3	CTRCT6	VWM3	EFNA1	BHC110
MMACHC	HDFNRH	ECK	DEE15	DPM3	ARCC2
PRDX1	RHNA	PARK9	NEDSWMA	MUC1	HPPO
POMGNT1	ARH	SDH2	MYH	GBA1	CPRF
RAD54L	SEPN1	PADI5	RP76	GON4L	KIAA0601
CYP4A11	LAP18	OZEMA16	PRXI	LMNA	HSAN2D
CYP4A22	RNF28	RMS2	MEB	NTRK1	DEE11
TAL1	CDW52	ALDH4	HR54	SPTA1	HMAD
CMPK1	HDS	ZUBR1	CMK	KCNJ10	FEB3A
FOXE3	HPMRS1	KIAA0090	ASGD2	VANGL2	SFNP
ELAVL4	FHCL4	PLA2B	TCL5	SLAMF1	SRC2
PARK10	SHP	FRFB	CMPK	CD244	MGC1203
FAF1	C1orf172	PARK6	FKHL12	F11R	HPPI
RNF11	NHE1	OST	HUD	MPZ	SPAPA3
CPT2	HAKA1	ARCC2	AAOPD	NOS1AP	PSF
LRP8	CMH31	HOPS	HFAF1	DDR2	KIAA0607
NDC1	SRC2	PLC	AAT11	LMX1A	IRIP
DIO1	LIKNS	TKS	IIAE4	TMCO1	IPT
TMEM59	ASML3B	SERKAL	APOER2	GPR161	NEDMISBA
CDCP2	OPA16	C1QD1	TMEM48	F5	FACE1
PARS2	RP59	C1QG	TXDI1	KIFAP3	DFNA2A
DHCR24	LINC001000	C1QD2	DCF1	GORAB	KIAA0467
NEDMLOB	NRBF1	EPHT3	LGMDR15	PRRX1	GLYT1
OX40	CRTM	LSD1	DEE75	FASLG	HLD23
EDSSPD2	SYND3	HNRNPR	OX40	DARS2	NET4
ACT35	KIAA0099	HPPA	EDSSPD2	TNR	VWM3
ALGAZ	MGC1203	DBA7	CMD1LL	PDCN	SIL
MLM	IMD22	APT2	RC68	ACBD6	FKHL12
CCV	RPD3L1	THC13	CECBA	ASPM	RSDM1
C1DELp36	IMNEPD2	VWS2	PHRINL	CRB1	ORC1L
G1P2	YARS	AML2	CD137	PTPRC	FLJ10986
CMS8	FRCP2	CB2	MDS	DDX59	BRMUTD
TXGP1L	DYT2	CMH31	ACT35	TNNT2	RP20
SEMDJL1	YRS	IL28RA	ALGAZ	REN	GPI8
IMD38	DEAR1	SOM	PBD6A	CNTN2	CAFD2
PSF3L	YTS	CBFA3	MPB1	IL10	CMCS
DRS2	CX30.3	HDFNRH	TR2	MCP	PARG1
HAYOS	CX31	RHNA	LVNC8	HHAT	FASL
IMD16	CX37	ARH	LARD	DEL1q41q42	SDR2
MRD42	FECD1	SEPN1	CMTRIC	USH2A	SAS6
SGS	MCPIP	LAP18	CORTRD1	TGFB2	EDG1
IFI15	NEDHCS	RNF28	IMD14B	TLR5	STL2
NALD	P28	CDW52	LCA9	CAPN2	SCAMC1
CTRCT49	FLJ40906	HDS	NBLST1	DEGS1	LGN
HVEM	DFNA88	HPMRS1	DR3	WDR26	SARS
MEL1	IRIP	THMA2	HMNR4	LBR	ARCND1

CILD47	CGI94	SHP	G6PDH	TMEM63A	FND1
VEL	RAGC	C1orf172	IMD14A	LEFTB	DFNB82
SLSN4	IPT	NHE1	PNAT1	CCSAP	KREV1
PMNDS	TYRRS	HAKA1	CMT2A1	ACTA1	WNT13
RC68	CLN1	MURF1	CIROZ	DISC1	CAPPA1
USH1M	FACE1	SRC2	ALS10	TBCE	MCT1
TNFRSF12	EDM2	DEDSM	SKS	EDARADD	KIAA1634
KIAA0720	DFNA2A	ASML3B	SCCD	MTR	PEP
SPOC1	CTPS	LIKNS	ANP	GREM2	ALPS4
KIAA0833	EKVP1	OPA16	LLH	NLRP3	STBM2
PHRINL	UGN	LINC0010(	CMT2A2A	COLEC11	HEN2
ILA	CMD2C	NRBF1	D1S166E	SOX11	LFA3
PARK7	HOMG5	CRTM	TNFBR	KIDINS220	LCDD
RALT	LEPRE1	SYND3	EDSKCL1	TYHAQ	C1DELq21.1
PPH	CCDC23	KIAA0099	HMSN6A	PDIA6	RBM8B
MIRN34A	SC	MGC1203	MOM1	NBAS	SOFM
GDH	GLUT1	IMD22	AOMS4	MSGN1	URBWD
APDS	WDR65	RPD3L1	KIAA0842	MATN3	MGC3180
NMNAT	JTK14	YTS	KIAA1307	EIF2B4	RORG
CMT2A	TPOR	YARS	CTPA	FOSL2	EPLG3
MDS	OZEMA14	FRCP2	SPG78	SPAST	EPLG1
SRG	SSC1	DYT2	CTPP1	PKDCC	MDDGC15
C1orf127	LAR	IMNEPD2	KRPPD	DYNC2LI1	PUM
TDP43	OI8	DEAR1	SDHIP	SIX3	HGPS
PBD6A	SIAT6	YRS	PAD	CRIPT	KIAA1606
FRAP1	DPH2L2	CX30.3	PPGL4	RTN4	LMN1
TERE1	GLYT1	CX31	CMYO19	EFEMP1	TRKA
PBD6B	NET4	CX37	P5CDH	WDPCP	EL2
CLCN6	SDCHCN	FECD1	RBAF600	SPRED2	SESAME
PND	VWM3	MCPIP	CAVIPMR	ANTXR1	LTAP
BNP	DEE15	NEDHCS	PLA2L	TIA1	SLAM
LH1	NEDSWMA	P28	MC2DN4	ACTG2	NAIL
KIAA0214	MYH	FLJ40906	HPPC	DGUOK	JAM1
CD30	LGMDR15	DFNA88	OST48	SLC4A5	CMT1B
TNFR2	PRXI	IRIP	HPPI	DCTN1	CAPON
CMT2A2B	MEB	CGI94	TNSALP	SUCLG1	NTRKR3
SWS1	HR54	RAGC	SJS	RPIA	LMX1
CLCR	RP76	IPT	BDPLT22	IGKC	CFSMR1
ELA2A	MDDGC3	DYT18	AIMAH3	CNGA3	ALPS1B
SKIP	TCL5	CLN1	FHCL4	RANBP2	THPH2
ATFB6	CMPK	FACE1	C1QD3	POLR1B	SMAP
EDSKCL1	FKHL12	EDM2	CDG1R	CKAP2L	SCYL1BP1
HMSN6A	HUD	DFNA2A	DRT	IL1A	PMX1
ECK	AAOPD	CTPS	AOF2	IL1B	TNFSF6
PARK9	HFAF1	OI8	NEDDFSB	IL1RN	ASPRS. LBSL
SDH2	AAT11	UGN	CAPB	DPP10	NEDSTO
PADI5	IIAE4	CMD2C	CPRF	CFAP221	NPHS2
OZEMA16	APOER2	HOMG5	PCBC	EPB41L5	NEDPM
RMS2	TMEM48	LEPRE1	KIAA0601	RNU4ATAC	MCPH5
ALDH4	TXDI1	CCDC23	ERK	CFC1	RP12
ZUBR1	DCF1	SC	BHC110	DARS1	CD45
KIAA0090	CMK	GLUT1	CX5	LRP1B	OFD5
PLA2B	DEE75	WDR65	HPPO	ZEB2	CMH2
FRFB	KIAA0018	JTK14	SJS1	GEFSP7	RTD
PARK6	CORTRD1	TPOR	TFCP2L4	MBD5	TAX
OST	IMD14B	OZEMA14	PEBP2A3	MMADHC	CSIF
CTRCT6	LCA9	SSC1	FHCB1	ITGB6	CD46
HOPS	NBLST1	LAR	CMYO3	SLC4A10	MART2
PLC	CMTDIC	MRT12	FHCB2	SCN2A	C1DELq41q42
TKS	GAMOS10	SIAT6	SELN	GALNT3	RP39
SERKAL	DR3	DPH2L2	SMN	SCN1A	LDS4
C1QD1	HMNR4	GLYT1	SMRZ	SCN9A	TIL3
C1QG	CMD1LL	NET4	ECTD12	BBS5	CMYO2C
C1QD2	CECBA	IS6	RP59	SP9	DES1
EPHT3	IMD109	VWM3	DYTOABG	GPR155	SKDEAS
LSD1	CD137	DEE15	CMP	CHN1	PHA
HNRNPR	TR2	NEDSWMA	SDCN	HOXD13	KIAA0792
HPPC	LVNC8	MYH	NEDMSF	PDE11A	LEFTY1
DBA7	MPB1	RP76	EKVP2	ITGA4	CSAP
APT2	LARD	PRXI	DFNA2B	FRZB	ASMA
THC13	G6PDH	MEB	TYRRS	COL3A1	SCZD9
BDPLT22	IMD14A	HR54	PPCD2	STAT4	KCS
AIMAH3	PNAT1	CMK	MCPIP1	STK17B	ED3
CB2	CMT2A1	ASGD2	APNH	SATB2	HMAG
CX5	CMTRIC	TCL5	SPGF83	AOX1	PRDC
IL28RA	HTX14	CMPK	CMTDIC	NIF3L1	CIAS1
SOM	CIROZ	FKHL12	GAMOS10	HPPC	CLK1
CBFA3	ALS10	HUD	LNGODS	C1orf170	MRD27
HDFNRH	ATRST2	AAOPD	COXPD35	DRS2	ARMS
RHNA	SKS	HFAF1	MADB	SGS	OS2
ARH	SCCD	AAT11	EKVP1	MEL1	ERP5
SEPN1	ROCHIS	IIAE4	STE24	PARK7	NAG
LAP18	SHILCA	APOER2	STL5	GDH	RLSDF
RNF28	ANP	TMEM48	RSDM1	CMT2A	EDM5
CDW52	MSL	TXDI1	IMD24	TDP43	VWM4
HDS	LLH	DCF1	LMPHM11	FRAP1	ACED
HPMRS1	CMT2A2A	LGMDR15	THCYT2	CLCN6	SPG4
FHCL4	D1S166E	DEE75	GROS1	KIAA0214	VLK
SHP	TNFBR	OX40	NEDAHM	TNFR2	D2LIC
C1orf172	CTPA	EDSSPD2	RD	SKIP	HPE2
NHE1	SPG78	CMD1LL	HTLVR	ECK	RTS3
HAKA1	PPGL4	RC68	SPGF95	HOPS	NOGO
CMH31	AOMS4	CECBA	TIE1	TKS	FBNL
SRC2	KIAA0842	PHRINL	MPLV	LSD1	C2orf86
LIKNS	ARCC2	CD137	PED	LAP18	NS14
ASML3B	MC2DN4	MDS	IKSHD	C1orf4	TEM8
OPA16	KIAA1307	ACT35	BNAH2	C1orf172	WDM
RP59	CTPP1	ALGAZ	GLUT1DS	PGR22	ACTA3
LINC0010(	KRPPD	PBD6A	ST3GALII	CHN	DGK
NRBF1	SDHIP	MPB1	DEDSSH2	HOX4I	NBC4
CRTM	PAD	TR2	GCENSG	PDE11A1	HMND14
SYND3	MOM1	LVNC8	CTDI	CD49D	SUCLA1
KIAA0099	CMYO19	LARD	SDCHCN	FRZB1	RPI
MGC1203	P5CDH	CMTRIC	DYT9	EDSVASC	IGKCD
IMD22	RBAF600	CORTRD1	EIG12	SLEB11	CNG3
RPD3L1	CAVIPMR	IMD14B	SPG83	DRAK2	NUP358
IMNEPD2	PLA2L	LCA9	NKEFA	KIAA1034	TCS4
YARS	HPPI	NBLST1	MDDGB3	CMD1LL	RADMIS
FRCP2	CDG1R	DR3	PAGA	CORTRD1	NCPH1
DYT2	OST48	HMNR4	MDDGA3	NBLST1	ARCL1D
YRS	HPPA	G6PDH	HRAD54	LVNC8	MVCD4
DEAR1	TNSALP	IMD14A	UMK	AIMAH3	DPRP3
YTS	SJS	PNAT1	CTRCT34	G6PDH	PCDP1
CX30.3	HPPO	CMT2A1	SCL	CMT2A1	KIAA1548
CX31	SJS1	CIROZ	UMPK	ALS10	U4ATAC
CX37	SJA	ALS10	ASMD	SKS	CRYPTIC
FECD1	C1QD3	SKS	PNEM	MSL	DARS
MCPIP	KIAA0601	SCCD	MDDGC3	CMT2A2A	TALS
NEDHCS	DRT	ANP	MCI1	TNFBR	ZFHX1B
P28	AOF2	LLH	NEDAPA	KIAA0842	FEB3B
FLJ40906	NEDDFSB	CMT2A2A		CTPP1	KIAA1461
DFNA88	ECTD12	D1S166E		TNSALP	C2orf25
IRIP	APNH	TNFBR		CTRCT6	AI1H
CGI94	VWS2	EDSKCL1		AOF2	GLSS
RAGC	AML2	HMSN6A		SMN	SCN2A1
IPT	CAPB	MOM1		B120	HHS
TYRRS	CPRF	AOMS4		ECTD12	GEFSP2
CLN1	FHCB1	KIAA0842		CTRCT34	NENA
FACE1	CMYO3	KIAA1307		CSS2	LYCHOS
EDM2	PCBC	CTPA		MRD14	ARHGAP2
DFNA2A	TFCP2L4	SPG78		SMARCF1	SPD1
CTPS	PEBP2A3	CTPP1		CTPA	PDE11A2
EKVP1	MURF1	KRPPD		HPPA	RHOGAP2
UGN	DEDSM	SDHIP		NEDIES	BDSD
CMD2C	FHCB2	PAD		GAMOS10	PDE11A3
HOMG5	SELN	PPGL4		COXPD35	OS1
LEPRE1	SMN	CMYO19		AAT11	MCPH14
CCDC23	SMRZ	P5CDH		STE24	S1P1
SC	ERK	RBAF600		MADB	DFNA37
GLUT1	BHC110	CAVIPMR		DEE18	NEDMAS
WDR65	DYTOABG	PLA2L		GCENSG	PINS
JTK14	CMP	MC2DN4		ASGD2	SERS
TPOR	SDCN	HPPC		CTDI	LCA2
OZEMA14	NEDMSF	OST48		IS6	NEDHCAS
SSC1	EKVP2	HPPI		MCPH7	NCMS
LAR	DFNA2B	TNSALP		ASMD	MCT1D

OI8	LNGODS	SJS	CMT2A2B	XWNT2
SIAT6	PPCD2	BDPLT22	HMSN6A	LYP
DPH2L2	MCPIP1	AIMAH3	BHC110	HHF7
GLYT1	COXPD35	FHCL4	ARCC2	CMNS
NET4	SPGF83	C1QD3	HPPO	PTPN8
SDCHCN	STE24	CDG1R	CPRF	NS6
VWM3	STL5	DRT	KIAA0601	DEL1q21.1
DEE15	IMD24	AOF2	HSAN2D	HH27
NEDSWMA	MADB	NEDDFSB	DEE11	IMD42
MYH	RSDM1	CAPB	HMAD	CMD1A
LGMDR15	DYT18	CPRF	FEB3A	HPP
PRXI	GROS1	PCBC	SFNP	TAR
MEB	NEDAHM	KIAA0601	SRC2	FPLD2
HR54	RD	ERK	MGC1203	HS3
RP76	HTLVR	BHC110	HPPI	OFD19
MDDGC3	SPGF95	CX5	SPAPA3	RZRG
TCL5	TIE1	HPPO	PSF	SPH3
CMPK	MPLV	SJS1	KIAA0607	TNFAIP4
FKHL12	DYT9	TFCP2L4	IRIP	MDDGB15
HUD	IKSHD	PEBP2A3	IPT	ADTKD2
AAOPD	BNAH2	FHCB1	NEDMISBA	IMD105
HFAF1	MRT12	CMYO3	FACE1	RCM3
AAT11	ST3GALII	FHCB2	DFNA2A	EPEO5
IIAE4	DEDSSH2	SELN	KIAA0467	CMYO2A
APOER2	GCENSG	SMN	GLYT1	NALP3
TMEM48	CTDI	SMRZ	HLD23	LTMS
TXDI1	EIG12	ECTD12	NET4	EMD2
DCF1	GLUT1DS	RP59	VWM3	CDW150
CMK	PED	DYTOABG	SIL	NKR2B4
DEE75	SPG83	CMP	FKHL12	CHN2
KIAA0018	LMPHM11	SDCN	RSDM1	CMTDID
CORTRD1	THCYT2	NEDMSF	ORC1L	KIAA0464
IMD14B	PAGA	EKVP2	FLJ10986	TKT
LCA9	MDDGA3	DFNA2B	BRMUTD	DFNA7
NBLST1	HRAD54	TYRRS	RP20	CD150
CMTDIC	NKEFA	PPCD2	GPI8	SLAMF4
GAMOS10	MDDGB3	MCPIP1	CAFD2	RPRGL1
DR3	SCL	APNH	CMCS	DSS
HMNR4	UMPK	SPGF83	PARG1	NTKLBP1
CMD1LL	ASMD	CMTDIC	FASL	PHOX1
CECBA	PNEM	GAMOS10	SDR2	APT1LG1
IMD109	IS6	LNGODS	SAS6	NPHS22
CD137	UMK	COXPD35	EDG1	WRCN
TR2	CTRCT34	MADB	STL2	SRN1
LVNC8	ASGD2	EKVP1	SCAMC1	GO
MPB1	MCI1	STE24	LGN	AGOTC
LARD	NEDAPA	STL5	SARS	LCA8
G6PDH	THMA2	RSDM1	ARCND1	LCA
IMD14A		IMD24	FND1	LVNC6
PNAT1		LMPHM11	DFNB82	CMD1D
CMT2A1		THCYT2	KREV1	ADTKD4
CMTRIC		GROS1	WNT13	TAX1
HTX14		NEDAHM	CAPPA1	GVHDS
CIROZ		RD	MCT1	AHUS2
ALS10		HTLVR	KIAA1634	SKI1
ATRST2		SPGF95	PEP	NNMS
SKS		TIE1	ALPS4	MELIOS
SCCD		MPLV	STBM2	CAEND2
ROCHIS		PED	HEN2	SLEB1
SHILCA		IKSHD	LFA3	HLD18
ANP		BNAH2	LCDD	MLD
MSL		GLUT1DS	C1DELq21.1	PYPAF1
LLH		ST3GALII	RBM8B	PHASK
CMT2A2A		DEDSSH2	SOFM	HLD19
D1S166E		GCENSG	URBWD	SHPM
TNFBR		CTDI	MGC3180	C1orf96
CTPA		SDCHCN	RORG	NEM3
SPG78		DYT9	EPLG3	CMYO2B
PPGL4		EIG12	EPLG1	KCS1
AOMS4		SPG83	MDDGC15	EDA3
KIAA0842		NKEFA	PUM	ILFS2
ARCC2		MDDGB3	HGPS	STHAG9
MC2DN4		PAGA	KIAA1606	FCU
KIAA1307		MDDGA3	LMN1	3MC2
CTPP1		HRAD54	TRKA	IDDMOH
KRPPD		UMK	EL2	SINO
SDHIP		CTRCT34	SESAME	KEFH
PAD		SCL	LTAP	DFNA34
MOM1		UMPK	SLAM	SOPH
CMYO19		ASMD	NAIL	FCAS1
P5CDH		PNEM	JAM1	HOA
RBAF600		MDDGC3	CMT1B	VENARG
CAVIPMR		MCI1	CAPON	HRD
PLA2L		NEDAPA	NTRKR3	ECTD11B
HPPI			LMX1	SGK493
CDG1R			CFSMR1	LIC3
OST48			ALPS1B	GLC1H
HPPA			THPH2	CHDTHP
TNSALP			SMAP	SEMDBCD
SJS			SCYL1BP1	DHRD
HPPO			PMX1	BBS15
SJS1			TNFSF6	GAPO
SJA			ASPRS. LBSL	ATR
C1QD3			NEDSTO	ALS26
KIAA0601			NPHS2	VSCM1
DRT			NEDPM	MTDPS3
AOF2			MCPH5	MMIHS5
NEDDFSB			RP12	PEOB4
ECTD12			CD45	MTDPS9
APNH			OFD5	RPIAD
VWS2			CMH2	SRTD15
AML2			RTD	ACHM2
CAPB			TAX	ANE1
CPRF			CSIF	PEAMO
FHCB1			CD46	ECTD11A
CMYO3			MART2	MACD
PCBC			C1DELq41q42	DEE6A
TFCP2L4			RP39	DIRA
PEBP2A3			LDS4	KIAA1492
MURF1			TIL3	CILD55
DEDSM			CMYO2C	IIAE3
FHCB2			DES1	MOPD1
SELN			SKDEAS	HTX2
SMN			PHA	HBSL
SMRZ			KIAA0792	SIP1
ERK			LEFTY1	SMADIP1
BHC110			CSAP	CRMO2
DYTOABG			ASMA	MRD1
CMP			SCZD9	MAHCD
SDCN			KCS	BFIS3
NEDMSF			ED3	RFMN
EKVP2			HMAG	BFIC3
DFNA2B			PRDC	HFTC1
LNGODS			CIAS1	SMEI
PPCD2			CLK1	PN1
MCPIP1			MRD27	BFNIS
COXPD35			ARMS	EA9
SPGF83			OS2	DRVT
STE24			ERP5	FHM3
STL5			NAG	LWS
IMD24			RLSDF	DEE6B
MADB			EDM5	PPNAD2
RSDM1			VWM4	SRFP3
DYT18			ACED	PMGEDSV
GROS1			SPG4	DPMC
NEDAHM			VLK	DURS2
RD			D2LIC	
HTLVR			HPE2	
SPGF95			RTS3	
TIE1			NOGO	
MPLV			FBNL	
DYT9			C2orf86	
IKSHD			NS14	
BNAH2			TEM8	
MRT12			WDM	
ST3GALII			ACTA3	

DEDSSH2
GCENSG
CTDI
EIG12
GLUT1DS
PED
SPG83
LMPHM11
THCYT2
PAGA
MDDGA3
HRAD54
NKEFA
MDDGB3
SCL
UMPK
ASMD
PNEM
IS6
UMK
CTRCT34
ASGD2
MCI1
NEDAPA
THMA2

DGK
NBC4
HMND14
SUCLA1
RPI
IGKCD
CNG3
NUP358
TCS4
RADMIS
NCPH1
ARCL1D
MVCD4
DPRP3
PCDP1
KIAA1548
U4ATAC
CRYPTIC
DARS
TALS
ZFHX1B
FEB3B
KIAA1461
C2orf25
AI1H
GLSS
SCN2A1
HHS
GEFSP2
NENA
LYCHOS
ARHGAP2
SPD1
PDE11A2
RHOGAP2
BDSD
PDE11A3
OS1
MCPH14
S1P1
DFNA37
NEDMAS
PINS
SERS
LCA2
NEDHCAS
NCMS
MCT1D
XWNT2
LYP
HHF7
CMNS
PTPN8
NS6
DEL1q21.1
HH27
IMD42
CMD1A
HPP
TAR
FPLD2
HS3
OFD19
RZRG
SPH3
TNFAIP4
MDDGB15
ADTKD2
IMD105
RCM3
EPEO5
CMYO2A
NALP3
LTMS
EMD2
CDW150
NKR2B4
CHN2
CMTDID
KIAA0464
TKT
DFNA7
CD150
SLAMF4
RPRGL1
DSS
NTKLBP1
PHOX1
APT1LG1
NPHS22
WRCN
SRN1
GO
AGOTC
LCA8
LCA
LVNC6
CMD1D
ADTKD4
TAX1
GVHDS
AHUS2
SKI1
NNMS
MELIOS
CAEND2
SLEB1
HLD18
MLD
PYPAF1
PHASK
HLD19
SHPM
C1orf96
NEM3
CMYO2B
KCS1
EDA3
ILFS2
STHAG9
FCU
3MC2
IDDMOH
SINO
KEFH
DFNA34
SOPH
FCAS1
HOA
VENARG
HRD
ECTD11B
SGK493
LIC3
GLC1H
CHDTHP
SEMDBCD
DHRD
BBS15
GAPO
ATR
ALS26
VSCM1
MTDPS3
MMIHS5
PEOB4

MTDPS9
RPIAD
SRTD15
ACHM2
ANE1
PEAMO
ECTD11A
MACD
DEE6A
DIRA
KIAA1492
CILD55
IIAE3
MOPD1
HTX2
HBSL
SIP1
SMADIP1
CRMO2
MRD1
MAHCD
BFIS3
RFMN
BFIC3
HFTC1
SMEI
PN1
BFNIS
EA9
DRVT
FHM3
LWS
DEE6B
PPNAD2
SRFP3
PMGEDSV
DPMC
DURS2
